# International pooled patient-level meta-analysis of ketamine infusion for depression: In search of clinical moderators

**DOI:** 10.1038/s41380-022-01757-7

**Published:** 2022-09-07

**Authors:** Rebecca B. Price, Nicholas Kissel, Andrew Baumeister, Rebecca Rohac, Mary L. Woody, Elizabeth D. Ballard, Carlos A. Zarate, William Deakin, Chadi G. Abdallah, Adriana Feder, Dennis S. Charney, Michael F. Grunebaum, J. John Mann, Sanjay J. Mathew, Bronagh Gallagher, Declan M. McLoughlin, James W. Murrough, Suresh Muthukumaraswamy, Rebecca McMillan, Rachael Sumner, George Papakostas, Maurizio Fava, Rebecca Hock, Jennifer L. Phillips, Pierre Blier, Paulo Shiroma, Peter Šóš, Tung-Ping Su, Mu-Hong Chen, Mikael Tiger, Johan Lundberg, Samuel T. Wilkinson, Meredith L. Wallace

**Affiliations:** 1 University of Pittsburgh, Pittsburgh, PA USA; 2 Carnegie Mellon University, Pittsburgh, PA USA; 3 National Institutes of Health, Bethesda, MD USA; 4 University of Manchester, Manchester, UK; 5 Baylor College of Medicine, Houston, TX USA; 6 Michael E. Debakey VA Medical Center, Houston, TX USA; 7 Icahn School of Medicine at Mount Sinai, New York, NY USA; 8 Columbia University, New York, NY USA; 9 Trinity College Dublin, Dublin, Ireland; 10 University of Auckland, Auckland, New Zealand; 11 Harvard Medical School, Boston, MA USA; 12 University of Ottawa, Ottawa, ON Canada; 13 Minneapolis VA Medical Center, Minneapolis, MN USA; 14 Psychiatrie Slaný s.r.o., Slaný, Czech Republic; 15 National Yang-Ming University, Taipei, Taiwan; 16 Karolinska Institutet, Stockholm, Sweden; 17 Yale School of Medicine, New Haven, CT USA

**Keywords:** Depression, Psychology

## Abstract

Depression is disabling and highly prevalent. Intravenous (IV) ketamine displays rapid-onset antidepressant properties, but little is known regarding which patients are most likely to benefit, limiting personalized prescriptions. We identified randomized controlled trials of IV ketamine that recruited individuals with a relevant psychiatric diagnosis (e.g., unipolar or bipolar depression; post-traumatic stress disorder), included one or more control arms, did not provide any other study-administered treatment in conjunction with ketamine (although clinically prescribed concurrent treatments were allowable), and assessed outcome using either the Montgomery-Åsberg Depression Rating Scale or the Hamilton Rating Scale for Depression (HRSD-17). Individual patient-level data for at least one outcome was obtained from 17 of 25 eligible trials [pooled *n* = 809]. Rates of participant-level data availability across 33 moderators that were solicited from these 17 studies ranged from 10.8% to 100% (median = 55.6%). After data harmonization, moderators available in at least 40% of the dataset were tested sequentially, as well as with a data-driven, combined moderator approach. Robust main effects of ketamine on acute [~24-hours; β*(95% CI) = 0.58 (0.44, 0.72); *p* < 0.0001] and post-acute [~7 days; β*(95% CI) = 0.38 (0.23, 0.54); *p* < 0.0001] depression severity were observed. Two study-level moderators emerged as significant: ketamine effects (relative to placebo) were larger in studies that required a higher degree of previous treatment resistance to federal regulatory agency-approved antidepressant medications (≥2 failed trials) for study entry; and in studies that used a crossover design. A comprehensive data-driven search for combined moderators identified statistically significant, but modest and clinically uninformative, effects (effect size *r* ≤ 0.29, a small-medium effect). Ketamine robustly reduces depressive symptoms in a heterogeneous range of patients, with benefit relative to placebo even greater in patients more resistant to prior medications. In this largest effort to date to apply precision medicine approaches to ketamine treatment, no clinical or demographic patient-level features were detected that could be used to guide ketamine treatment decisions.

**Review Registration:** PROSPERO Identifier: CRD42021235630

## Introduction

Ketamine is a glutamatergic agent used routinely for induction and maintenance of anesthesia. In randomized controlled trials (RCTs), subanesthetic (typically, 0.5 mg/kg) intravenous (IV) ketamine exhibits well-replicated, rapid, potent antidepressant effects (i.e., study-level meta-analytic Cohen’s *d*’s ≥ 1.0 [1], reflecting large effects) in difficult-to-treat conditions such as treatment-resistant depression [2] and bipolar depression [3]. Antidepressant effects are detected within approximately 2 hours post-infusion (after acute dissociative and euphoric side effects subside) and continue far beyond the drug’s elimination half-life of 2.5-3 hours. Ketamine is now administered outside of research environments, including in hospital settings and specialized “ketamine therapy” clinics. However, IV ketamine’s clinical potential has been limited by practicalities including lack of insurance coverage for this off-label prescribing practice, high out-of-pocket expense to patients in many healthcare systems, burden on patients and the healthcare system due to ketamine’s side effect profile and administration routes, and concerns for abuse liability [4–6]. Such limitations may nevertheless be offset among a subset of patients for whom a strong, rapid response to ketamine administration is highly likely. But to date, there is limited understanding of *which* patients are likely to experience robust benefit.

Because IV ketamine’s effect size at a group level is typically large, randomized controlled trials (RCTs) have routinely been conducted with small sample sizes. Although such studies are adequately powered to detect ketamine’s effects at the group level, individual RCTs are often under-powered for conducting moderator analyses—i.e., analyses of baseline characteristics that can indicate *which* patients experience more benefit from ketamine relative to a comparator. Moderator analyses may yield smaller effect sizes, necessitating larger samples, and rely on sufficient heterogeneity within study participants. Although some predictors of ketamine’s antidepressant efficacy, including clinical (e.g., family history of alcohol use disorder [7, 8]; suicide history [9]; body mass index (BMI) [9]; benzodiazepine use [10]) and mechanistic (e.g., neuroimaging [11–13]; cognitive [14]; peripheral blood markers [15, 16]; genetic [17, 18]) variables, have been reported, none have been replicated across more than one RCT [19, 20]. RCT designs are essential to separate specific from non-specific predictors of outcome, but many predictive analyses have been conducted in ketamine-treated patients alone. Study-level meta-analyses have likewise not identified reliable moderators of effect size across trials [21, 22]. A more powerful meta-analytic approach is therefore needed to guide clinical treatment decisions, ideally focusing on moderators that can be readily measured in clinical settings.

The current study therefore employed a pooled patient-level ‘mega-analytic’ approach using participant-level data from RCTs of IV ketamine, administered to individuals experiencing depressive symptoms. While preserving the advantages of conventional meta-analysis as a means of aggregating evidence across numerous studies (overcoming certain limitations of individual studies, e.g. small sample size), patient-level ‘mega-analysis’ (also known as individual participant data meta-analysis) offers unique advantages, including an order-of-magnitude increase in data points analyzed for each variable (many per study rather than one summary measure per study)—which substantially increases statistical power, particularly for testing moderators [23]—and the ability to test hypotheses not able to be adequately tested in the individual original studies. We aimed to clarify the potential role of IV ketamine in the treatment of depression by: (1) characterizing the impact of IV ketamine (vs. control groups) on continuous and dichotomous measures of depression, including clinically meaningful (response/remission) benchmarks; (2) identifying individual patient and study-level characteristics that moderate ketamine’s effect on symptoms, in the hopes of suggesting ways to maximize response rates through personalized patient prescriptions; (3) utilizing a data-driven ‘combined moderator’ approach to identify novel *combinations* of patient characteristics that together may enhance clinical prediction and decision-making accuracy for use in clinical settings.

## Methods

### Study identification and selection

The meta-analysis protocol was pre-registered at http://www.crd.york.ac.uk/PROSPERO/ (CRD42021235630). PubMed was searched over the period from inception to 01/19/2021 using the auto-expanding option encompassing all terms and synonyms related to the following search: “ketamine AND (randomized or RCT) AND depress*”. Published meta-analyses and reviews were checked for additional relevant studies. Two independent raters assessed eligibility of all records according to inclusion criteria (agreement = 87%), and a third rater (RBP) resolved all discrepant eligibility determinations (*n* = 70; 13% of abstracts reviewed). Based on a dimensional conceptualization of depression and to promote patient-level diagnostic heterogeneity, all studies retrieved through our systematic literature review (as described above) were considered eligible if they recruited individuals with a unipolar or bipolar depressive disorder or another highly comorbid disorder in which depressive symptoms are central (e.g., post-traumatic stress disorder), and in which depression scores were reported as an outcome. At least one IV ketamine administration was required. Studies giving ketamine in combination with additional study-administered treatments (e.g., ECT) were excluded to improve power for testing mechanistic hypotheses relevant to ketamine specifically; however, studies including patients on stable doses of other concomitant medications prescribed clinically were allowable. An RCT design was required to minimize bias. Allowable control conditions included inert or psychoactive placebo, wait-list, or treatment-as-usual. Finally, to maximize data points while using uniform outcome measures across studies, depression outcome measures were selected as those most frequently reported in ketamine studies. Two outcomes emerged as most prevalent: (1) the Montgomery-Åsberg Depression Rating Scale (MADRS [24]), and (2) the Hamilton Rating Scale for Depression (17-item version; HRSD [25]). Both are widely used, well-validated, clinician-rated measures of depression severity.

Authors of eligible studies were invited, via email, to contribute data. Repeated attempts were made if no response was received. The following data were requested per-participant, with authors asked to contribute all available variables: drug condition, infusion order (relevant for crossover studies), pre- and post-infusion MADRS and HRSD-17 scores, and 33 potential moderator variables (detailed below). For post-infusion scores, the target timepoints relative to the infusion date were 24-hours (“rapid”) and 7 days (“post-rapid”) following a single infusion, and this precise protocol was available in 66.7% of contributing studies; however, deviations from these designs in a subset of included studies were allowable if the “rapid” outcome was collected between 4 hours and 3 days after a single infusion (with no additional infusions given in the interim), and if the “post-rapid” outcome was collected between 6 and 14 days following a first infusion, even if subsequent infusions were also given within that interval (see Table 1 for protocol details of all included studies). Anxiety (Hamilton Anxiety Rating Scale) and suicidal ideation (Beck Scale for Suicide Ideation) at baseline and 24-hours were also solicited as potential exploratory outcomes but were provided by too few studies to be considered usable (≤33.3%).

**Table 1:** Description of included studies.

Investigator(s)	Study country	Patients^a^	Ketamine protocol	Control condition(s)	Study Design	Minimum ADM treatment failures required for eligibility	Excluded psychiatric diagnoses/conditions	Concurrent psych meds	Eligible Depression Measures Provided
Deakin, Abdallah [53, 54]	UK	MDD outpatients (*n* = 37)	Single infusion 0.5 mg/kg	Saline; Lanicemine^b^	Parallel Arm	Unspecified (no requirement)	Lifetime history of psychosis, bipolar disorder, or alcohol/substance use disorder, not right-handed, consuming more than 10 cigarettes or 8 cups of caffeinated beverages per day, positive tox screen	Unmedicated/washout period	MADRS
Feder #1 [46]	US	PTSD outpatients (*n* = 30)	Six infusions^b^ 0.5 mg/kg	Midazolam	Parallel Arm	Unspecified (no requirement)	Active suicidal/homicidal ideation, lifetime history of psychotic or bipolar disorder, current anorexia or bulimia, alcohol/substance use disorder in previous 3 months, history of recreational ketamine/PCP use >1x or any in the past 2 years, current long-acting benzodiazepine or opioid medication.	Stable meds continued	MADRS
Feder #2 [55]	US	PTSD outpatients (*n* = 41)	Single infusion 0.5 mg/kg	Midazolam	Crossover	Unspecified (no requirement)	Active suicidal/homicidal ideation, lifetime history of psychotic or bipolar disorder, current bulimia or anorexia, alcohol/substance abuse/dependence in previous 3 months.	Unmedicated/washout period	MADRS
Grunebaum, Mann [56, 57]	US	Inpatients with MDD or BPI, II, or NOS w/ current MDE and suicidal ideation (*n* = 96)	Single Infusion 0.5 mg/kg	Midazolam	Parallel Arm	Unspecified (no requirement)	ECG abnormalities, current psychosis, history of drug or alcohol dependence within 6 months, suicidality due to substance use or withdrawal	Stable meds continued	HRSD^c^
Mathew, Murrough [58]	US	MDD outpatients (*n* = 73)	Single Infusion 0.5 mg/kg	Midazolam	Parallel Arm	3	Suicide/homicide risk, history of bipolar disorder, psychotic symptoms, and substance abuse within previous 2 years of enrollment	1 week unmedicated washout (4 weeks for fluoxetine), except for stable nonbenzodiazepine hypnotics	MADRS
McLoughlin, Gallagher [59]	Ireland	MDD or BP inpatients (*n* = 25)	Four infusions^b^ 0.5 mg/kg	Midazolam	Parallel Arm	Unspecified (no requirement)	Current involuntary admission, active SI, dementia, history of Axis 1 diagnosis other than a MDE, ECT within 2 months or alcohol/substance dependence within 6 months of enrollment	Stable meds continued	HRSD^c^
Murrough [60]	US	Any non-exclusionary diagnosis Inpatient and Outpatient (*n* = 24)	Single infusion 0.5 mg/kg	Midazolam	Parallel Arm	Unspecified (no requirement)	Outpatients excluded with CSSRS score of 4 or 5, lifetime history of schizophrenia or primary psychotic disorder, current psychotic or manic symptoms, substance use disorder within 1 month of screening or positive urine tox, any lifetime abuse of ketamine or PCP	Stable meds continued	MADRS
Muthukumaraswamy, McMillan, Sumner [61, 62]	New Zealand	MDD outpatients (*n* = 30)	Single infusion as a 0.25 mg/kg bolus followed by a 0.25 mg/kg/hr infusion for 45 minutes for a total dose of 0.4375 mg/kg	Remifentanil	Crossover	2	Lifetime history of ketamine or PCP abuse, body weight <50 kg or >120 kg, any relevant psychiatric/neurological comorbidities including schizophrenia, psychosis, epilepsy, substance abuse/dependence, or acute suicidality	Stable meds continued	MADRS
Papakostas, Fava [29]	US	MDD Outpatients (*n* = 61)	Single infusion 0.1^d^, 0.2^d^, 0.5, or 1.0 mg/kg	Midazolam	Parallel Arm	2	Failure to achieve satisfactory response to >7 adequate ADM trials in the current major depressive episode, primary dx of Axis I disorder other than MDD, substance abuse/dependence within 6 months of screening, any history of ketamine or PCP use	Stable meds continued	MADRS, HRSD
Phillips [47]	Canada	MDD outpatients (*n* = 43)	Single infusion 0.5 mg/kg; followed by up to 10 open-label infusions^b^	Midazolam	Crossover	2 ADMs plus 2 augmentation strategies	History of substance abuse/dependence, BMI ≥ 35, history of mania/hypomania	Stable meds continued	MADRS
Shiroma [48]	US	MDD outpatients (*n* = 54)	One or six infusions^b^ 0.5 mg/kg	Midazolam	Parallel Arm	2	PTSD, mild to moderate TBI, psychosis-related disorder, bipolar disorder, and Axis 1 disorder other than MDD as primary presenting problem, history of alcohol/substance abuse within 6 months of screening, imminent suicide/homicide risk	Stable meds continued	MADRS
Šóš [63]	Czech Republic	MDD inpatients (*n* = 27)	Single infusion; loading dose of 0.27 mg/kg for the first 10 min, followed by a maintenance infusion of 0.27 mg/kg within 20 min	Saline	Crossover	Unspecified (no requirement)	Suicide risk, any current psychiatric comorbidity on Axis I and II, lifetime history of psychotic symptoms and psychotic disorder in first- or second-degree relatives	Stable meds continued	MADRS
Su, Chen [30]	Taiwan	MDD outpatients (*n* = 48)	Single Infusion 0.5 mg/kg or 0.2 mg/kg^d^	Saline	Parallel Arm	2	History of bipolar disorder, psychotic symptoms, and substance dependence	Stable meds continued	MADRS, HRSD
Tiger, Lundberg [13]	Sweden	MDD outpatients (*n* = 30)	Up to four infusions^b^ 0.5 mg/kg	Saline	Parallel Arm	1	No medications taken for current MDE, any antidepressant treatment response, BP disorder, psychosis, neurodevelopmental disorders, any comorbid primary diagnoses, body weight >100 kg, substance abuse, current suicidality	Unmedicated/washout period	MADRS
Zarate, Ballard #1 [39, 64]	US	Bipolar (I or II) depressed inpatients (*n* = 39)	Single infusion 0.5 mg/kg	Saline	Crossover	1	Current psychotic symptoms or lifetime psychotic disorder, active suicidal ideation (MADRS Suicide item > 4), substance abuse/dependence within 3 months of enrollment	2 weeks unmedicated washout period, except for one mood stabilizer at stable dose	MADRS, HRSD
Zarate, Ballard #2 [40]	US	MDD inpatients (*n* = 22)	Single infusion 0.5 mg/kg	Saline	Crossover	1	Current psychotic symptoms or lifetime psychotic disorder, active suicidal ideation (MADRS Suicide item > 4), substance abuse/dependence within 3 months of enrollment	2 weeks unmedicated washout period	HRSD^c^
Zarate, Ballard #3 [65]	US	MDD or bipolar (I or II) depressed inpatients (*n* = 40)	Single infusion 0.5 mg/kg	Saline	Crossover	1	Current psychotic symptoms or lifetime psychotic disorder, active suicidal ideation (MADRS Suicide item > 4), substance abuse/dependence within 3 months of enrollment	2 weeks unmedicated washout period, except for one mood stabilizer at stable dose (bipolar patients only)	MADRS, HRSD

*MADRS* Montgomery-Åsberg Depression Rating Scale, *HRSD* Hamilton Rating Scale for Depression (17-item version).

^a^*N* = number of unique patients with data provided and used in current primary analyses. Values may differ from those reported in original publications due to the eligible treatment conditions used in the pooled patient-level meta-analysis.

^b^For “rapid” timepoint, datapoint was approximately 1 day following the first infusion in the sequence of infusions. For “post-rapid” timepoint, the datapoint that was as close as possible to 7 days after the first infusion was used, even if subsequent serial infusions had been given within the ~7-day post-infusion period.

^c^To harmonize outcomes for primary analyses, MADRS scores were estimated from HRSD-17 scores according to a published conversion table.

^d^Data not included in analyses.

### Quality assessment and data extraction

Each contributing study team was asked to attest to specific methodological details (randomization, allocation concealment, blinding, and missing data). Responses were used to summarize the degree of protection against bias across 5 relevant criteria from the Cochrane Collaborations’ risk of bias tool [26]. Risk of bias based on the responses provided was uniformly low, with the exception of some risk of functional unblinding due to ketamine-specific side effects (details in Supplementary-1). Evidence for publication bias was also not found (Supplementary-1).

### Data harmonization

As shown in Table 1, 10 studies collected MADRS only, 3 studies collected HRSD-17 only, and 4 studies collected both MADRS and HRSD-17 scores. Given the higher prevalence of MADRS scores, to harmonize outcome measurement across all studies and maximize sample size for all analyses, a published score-to-score conversion algorithm [27] for depressed patients was utilized to estimate individual MADRS scores (at each timepoint) from HRSD-17 scores. Sensitivity analyses showed that studies where the MADRS was estimated did not significantly differ from other studies in terms of average MADRS scores or ketamine efficacy (Supplementary-1).

Due to high uniformity and application of consensus guidelines among ketamine clinical research [28], ketamine dosing, administration, and infusion methods were largely uniform across included studies (Table 1). Based on the strong preponderance of studies using 0.5 mg/kg ketamine dosing, and prior evidence of dose-response relationships [29, 30], primary analyses defined each patient’s treatment group as either (1) ≥0.5 mg/kg of intravenous ketamine or (2) placebo (inert or psychoactive). Patients receiving other ketamine doses (7.6% of patients), or other potentially active antidepressants (lanicemine; 2.4% of patients), were not included. In the minority of studies that utilized a crossover and/or repeated infusions design, we included only data relating to the first infusion that was given, thereby eliminating additional repeated within-subject measurements uniformly across all studies.

The 33 requested moderator variables were selected through consensus among study planners (RBP, EDB, CJZ, STW, SJM) to represent a comprehensive list based on previously reported moderation and prediction findings for ketamine and the study team’s knowledge of basic clinical (psychiatric and medical) and demographic information that is routinely collected in ketamine trials or was anticipated to be available in at least a subset of ketamine RCTs. The variables were returned in a range of formats and with highly variable data availability/compliance. For study-level characteristics used in descriptive and moderator analyses, design features were extracted by one rater (AB) and independently verified by a second rater (RBP). A single rater (RBP) then utilized a combination of automated (e.g., text string search) and hand-coding procedures to apply data harmonization techniques and create a uniform final set of dummy-coded (categorical) and continuous variables that maximized the capacity to analyze moderators uniformly across studies, as detailed in Table 2. In the final set of harmonized moderators (Table 2), availability of patient-level data ranged from 10.8% of patients to 100%, with a median of 55.6%. A second rater (MLWoody) independently verified all coded variables by cross-referencing the original source data; discrepant values were resolved by consensus.

**Table 2:** Moderators included in pooled dataset.

Domain	Sources of Information	Harmonized Variable(s)	% available	*N* available	Tier/Analysis	Summary statistics and case counts
Study design features	Study methods					
		Study Placebo Type	100.00	720	Tier 1^a^	*n* = 477 psychoactive; *n* = 243 inert
		Crossover or Parallel Arm	100.00	720	Tier 1^a^	*n* = 242 crossover; *n* = 478 parallel
Psychiatric diagnoses (principal and comorbid)	Patient-level text and ICD/DSM codes; study-level inclusions/exclusions	Dummy-Coded diagnoses:				
		Principal Diagnosis	100.00	720	Tier 1^b^	*n* = 579 MDD; *n* = 66 BD; *n* = 74 PTSD; *n* = 1 Borderline Personality Disorder
		Major Depressive Disorder (MDD)	100.00	720	Tier 1^b^	*n* = 627 yes; *n* = 93 no
		Bipolar (I or II) Disorder	99.44	716	None^c^	*n* = 66 yes; *n* = 650 no
		Post-traumatic Stress Disorder	72.50	522	Tier 2, M* 2d	*n* = 118 yes; *n* = 365 no
		Any Anxiety Disorder	72.36	521	Tier 2, M* 2d	*n* = 285 yes; *n* = 236 no
		Generalized Anxiety Disorder	64.58	465	Tier 2	*n* = 101 yes; *n* = 364 no
		Social Anxiety Disorder	58.06	418	Tier 2	*n* = 99 yes; *n* = 319 no
		Obsessive-Compulsive Disorder	57.92	417	Tier 2	*n* = 18 yes; *n* = 399 no
		Panic Disorder	57.78	416	Tier 2	*n* = 51 yes; *n* = 365 no
		Agoraphobia	57.78	416	Tier 2	*n* = 36 yes; *n* = 380 no
		Substance Use Disorder (lifetime)	42.92	309	Tier 2	*n* = 26 yes; *n* = 283 no
		Alcohol Use Disorder (lifetime)	40.28	290	Tier 2	*n* = 66 yes; *n* = 224 no
		Eating Disorder	36.11	260	None	*n* = 9 yes; *n* = 251 no
		Dysthymia	30.69	221	None	*n* = 31 yes; *n* = 190 no
		Any Pain Disorder	30.28	218	None	*n* = 48 yes; *n* = 170 no
		Attention-Deficit Disorder	27.64	199	None	*n* = 9 yes; *n* = 190 no
		Specific Phobia	26.94	194	None	*n* = 21 yes; *n* = 173 no
		Any Personality Disorder	25.42	183	None	*n* = 49 yes; *n* = 134 no
		Borderline Personality Disorder	24.31	175	None	*n* = 20 yes; *n* = 155 no
Demographics	Patient-level integers, text, and coded variables					
		Age	99.72	718	Tier 1^b^	mean = 42.4; SD = 13.3; range = 18–72
		Female or Male	99.58	717	Tier 1^b^	*n* = 386 yes; *n* = 331 no
		Years of Education	74.44	536	Tier 2, M* 2 g	mean = 14.6; SD = 2.9; range = 4–25
		Marital Status	50.83	366	Tier 2, M* 2 g	*n* = 127 married (or equivalent); *n* = 151 single/never married; *n* = 57 divorced or separated; *n* = 6 widowed; *n* = 25 not married (not further specified)
		Income Level/Range	15.83	114	None	*n* = 46 $0–25 K; *n* = 33 $25-50 K; *n* = 10 $50-70 K; *n* = 25 > $70 K
Race, Ethnicity, Culture	Patient-level text and coded variables					
		Study Performed in the US (y/n)	100.00	720	Tier 1^b^	*n* = 480 yes; *n* = 240 no
		EthnoRacial Category	89.58	645	Tier 2, M* 2a	*n* = 469 non-Hispanic white; *n* = 61 Black; *n* = 66 Asian; *n* = 26 Hispanic; *n* = 14 more than one race; *n* = 9 other non-White
		Hispanic/Latinx (y/n)	72.22	520	Tier 2	*n* = 29 yes; *n* = 491 no
Depression course and characteristics	Patient-level integers and coded variables; study-level inclusions/exclusions					
		Inpatient vs. Outpatient	100.00	720	Tier 1^b^	*n* = 461 outpatient; *n* = 259 inpatient
		Study-level TRD Threshold	100.00	720	Tier 1^b^	*n* = 280 no criterion; *n* = 71 1 failure; *n* = 253 2 failures; *n* = 16 ≥ 3 failures
		Suicidality severity at baseline (MADRS item 10)	70.42	507	Tier 2	mean = 2.01; SD = 1.5; range = 0–6
		Duration of Current MDE (months)^a^	61.11	440	Tier 2, M* 2c	mean = 52.5; SD = 95.7; range = 0.47–768
		Recurrent MDD (y/n)	55.14	397	Tier 2, M* 2c	*n* = 301 yes; *n* = 6 no
		Number of Depressive Episodes^a^	52.64	379	Tier 2, M* 2c	mean = 8.0; SD = 10.7; range = 1–78
		Age of illness onset	48.8	351	Tier 2	mean = 23.7; SD = 12.2; range = 4–60
		Number of failed ADM trials (patient-level)	42.78	308	Tier 2	mean = 4.44; SD = 2.3; range = 1–14
		Childhood trauma questionnaire (or any other available measure of childhood trauma)	20.3	146	None	mean = 56.4; SD = 22.4; range = 0–119
Concurrent Medications and Substances	Patient-level integers and coded variables; study-level inclusions/exclusions					
		Any Concurrent Psych Med (y/n)	76.25	549	Tier 2, M* 2b	*n* = 262 yes; *n* = 287 no
		Any Benzodiazepine (y/n)	65.56	472	Tier 2, M* 2b	*n* = 42 yes; *n* = 430 no
		Number of Concurrent Psych Meds	51.53	371	Tier 2	mean = 0.30; SD = 0.61; range = 0–3
		Smoker (Dummy-Coded)	53.47	385	Tier 2, M* 2 f	*n* = 109 yes; *n* = 76 no
Biological measurements	Patient-level integers					
		Ketamine dose (mg)	82.64	595	Tier 2	Ketamine arms only: mean = 42.1; SD = 15.4; range = 18.5–100.7^b^
		Dosing weight (kg)^a^	60.83	438	Tier 2	mean = 79.03; SD = 19.8; range = 35–174
		BMI^a^	55.56	400	Tier 2, M* 2 f	mean = 27.23; SD = 6.2; range = 17–60.8
		Systolic Blood Pressure	53.89	388	Tier 2, M* 2e	mean = 121.49; SD = 14.3; range = 86–166
		Diastolic Blood Pressure	53.89	388	Tier 2, M* 2e	mean = 75.24; SD = 10.2; range = 48–112
		Baseline Pulse^a^	52.22	376	Tier 2	mean = 70.89; SD = 12.7; range = 43–164
		Baseline serum BDNF level (ng/mL)	16.90	122	None	mean = 921.72; SD = 1629.1; range = 0 9419
		Baseline Pulse-Ox	17.60	127	None	mean = 97.77; SD = 1.9; range = 90–100
		Baseline Respiration Rate	10.83	78	None	mean = 15.44; SD = 3.6; range = 5–28

^a^Tier 1 variables are those available in >99% of patients in the pooled dataset. These first two Tier 1 variables, which related to study design features rather than to patient-level characteristics, were excluded from all combined moderator analyses, as the purpose was to identify combinations of patient (not study) characteristics that predicted differential improvement following ketamine (relative to placebo).

^b^Tier 1 variables are those available in >99% of patients in the pooled dataset. These 7 Tier 1 variables were related to patient-level characteristics and thus were included in the Tier 1 M*, and also retained in all M* analyses across all Tiers 2a-f, as their inclusion never reduced the number of studies/patients available for any analysis and could only increase predictive power for the data-driven approach.

^c^Bipolar I/II diagnosis was highly non-orthogonal and virtually redundant with the MDD diagnosis variable, as bipolar and unipolar depression diagnoses are mutually exclusive. To eliminate this redundancy in hypothesis tests, this variable was not tested as a moderator in any analysis.

### Statistical analysis

Analyses were conducted comparing IV ketamine doses of 0.5 mg/kg or greater vs. all placebo conditions, with inert and psychoactive placebo collapsed into one group (type of placebo condition was analyzed as a study-level moderator). Two outcomes were computed as the % improvement in MADRS score from pre-infusion to: (a) “rapid” post-infusion MADRS and (b) “post-rapid” post-infusion MADRS. MADRS response (≥50% decrease from pre-infusion) and remission (MADRS ≤ 9) rates were calculated to provide further descriptive information on the clinical main effects of ketamine vs. placebo, but were not used as outcomes in moderator analyses, given that the goal of these analyses was to explain heterogeneity of outcomes, which is maximally captured by continuous measures. Individual patient data analyses [31] were completed separately for “rapid” and “post-rapid” continuous outcomes using linear mixed effects regression models. All models included a random study effect to control for unobserved study heterogeneity; patient-level data was considered level 1 and study-level data was considered level 2. For interpretability, continuous variables were standardized and dichotomous variables were coded as 0 and 1. All analyses were performed using R version 3.6.3.

Completion rates were high in the contributing studies (≥90%) and risk-of-bias assessments (Supplementary-1) suggested low risk of bias from missing data [26]. The novel information obtainable through imputation was expected to be low due to high completion rates, the use of only two assessment points in each analysis, and the inability to impute across studies. Therefore, completer datasets were used for all analyses.

#### Main effects

We tested the main treatment effect for % improvement, response, and remission at the “rapid” and “post-rapid” time points. Standardized coefficients (β*) or odds ratios (OR) with 95% profile likelihood confidence intervals are reported for these outcomes. Number needed to treat (NNT) is also provided.

#### Sequential moderator analyses

Potential moderators were first tested sequentially. For each of the two outcome variables (% change in MADRS at rapid and post-rapid timepoints), models included the moderator variable, treatment, and their interaction term (moderator*treatment) as independent variables, with study as a random effect. A class of 9 moderator variables were non-redundant and available in ≥99.5% of patients and were therefore considered as primary (labeled “Tier 1”). Two-tailed *p*-values are reported with Bonferroni correction across these 9 variables; for completeness, unadjusted p-values are also reported. An additional set of 29 moderators were available in a minimum of 40% of patient-level datasets. These “Tier 2” variables, available in 40–82% of patients, were considered exploratory due to lower statistical power and low case counts for some patient features. Thus, Tier 2 *p-*values are unadjusted to minimize Type II error. The cut-point of ≥40% for inclusion in Tier 2 was determined based on a natural inflection point in the distribution of missingness (see Table 2), allowing for retention of 78% of all potential moderators, with a minimum of *n* = 288 patients in each individual moderator analysis. Five continuous moderator variables (Table 2) showing substantial deviations from normality per Q-Q plot inspection were log-transformed prior to analysis.

For each model, we extracted the standardized β (β*) and 95% confidence interval for the interaction term. We also computed the moderator effect size [32], *r*, with 95% bootstrap confidence intervals based on 200 samples. These effect sizes are Spearman correlations that indicate the strength with which a potential moderator distinguishes outcome differences between those receiving ketamine versus placebo. More positive *r* values indicate that higher values of an ordered moderator (or endorsing a categorical moderator) are associated with higher percentage improvement in depression scores for ketamine relative to placebo. As a benchmark to guide our interpretation of findings, for both individual and combined moderators, we considered only moderators with medium-to-large effect sizes (|*r* | ≥ 0.3) to be of sufficient explanatory power to be useful in guiding clinical decision-making.

#### Combined moderator analyses

A data-driven approach was taken to probe for combinations of moderator variables that jointly (as a weighted combination) predict efficacy of ketamine over placebo. The combined moderator is denoted M*. Its derivation has been described in detail previously [32, 33] and used successfully to identify combined moderators for randomized trials [34–36]. Briefly, the optimal combined moderator approach uses multivariable regularized regression to simultaneously estimate weights that quantify the extent to which each moderator distinguishes outcome differences between participants who received ketamine versus placebo. These weights are used to compute a new combined moderator, denoted M*. M* incorporates information across multiple potentially weak and/or contradictory moderators, thereby providing a single, stronger indication of the treatment on which an individual is likely to have a preferable outcome. Bootstrap confidence limits for M* were computed and used to determine statistical significance based on whether the CI crossed 0, as this approach to significance testing was robust to the nested study design.

As above, two separate models were run for each analysis, using (1) the rapid and (2) the post-rapid timepoints as the outcome variable. Tier 1 M* models included six Tier 1 variables that pertained to patient characteristics (M* #1). Two Tier 1 variables (crossover design; placebo type) were excluded from these analyses, because they pertained strictly to research study design features and inferences would not be generalizable to clinical treatment settings; and one additional Tier 1 variable (principal diagnosis) was omitted due to high overlap/redundancy with the Major Depressive Disorder (MDD) diagnosis dummy-coded variable already included. Next, 7 unique subsets of Tier 2 variables (M* #2a-2g) were constructed to organize moderator variables thematically (as shown in Table 2) while also maximizing the number of retained datapoints within each analysis. Given that each moderator variable in Tier 2 was available within a unique subset of studies, compiling numerous (i.e., ≥3) Tier 2 variables into a single M* analysis would necessitate reducing the total number of patients/studies available for use within that analysis. Thus, we opted to separately analyze the 7 unique moderator variable subsets (M* #2a-2g). Each of these Tier 2 M* analyses retained all six of the Tier 1 patient characteristic variables (the inclusion of these Tier 1 variables never reduced the number of studies/patients available for any analysis, due to >99% availability of each Tier 1 variable across the full dataset, and thus could only increase predictive power for the data-driven approach), while adding between 1 and 3 unique Tier 2 variables (see Table 2, “Tier/Analysis”). M* analyses in each Tier 2 level included a maximum of *n* = 632 (Tier 2a) and a minimum of *n* = 217 patients (Tier 2 f). As with the sequential analyses, for each M* we extracted the standardized beta for the interaction term and the moderator effect size *r*.

#### Non-specific predictor effects

Although our a priori focus was on moderators predicting *differential* response to ketamine vs. placebo, the non-specific effects (i.e., across ketamine and placebo arms) for each potential moderator variable were also quantified. This information is included in the full statistical output (Supplementary-1).

## Results

### Study selection

**Fig. 1:**
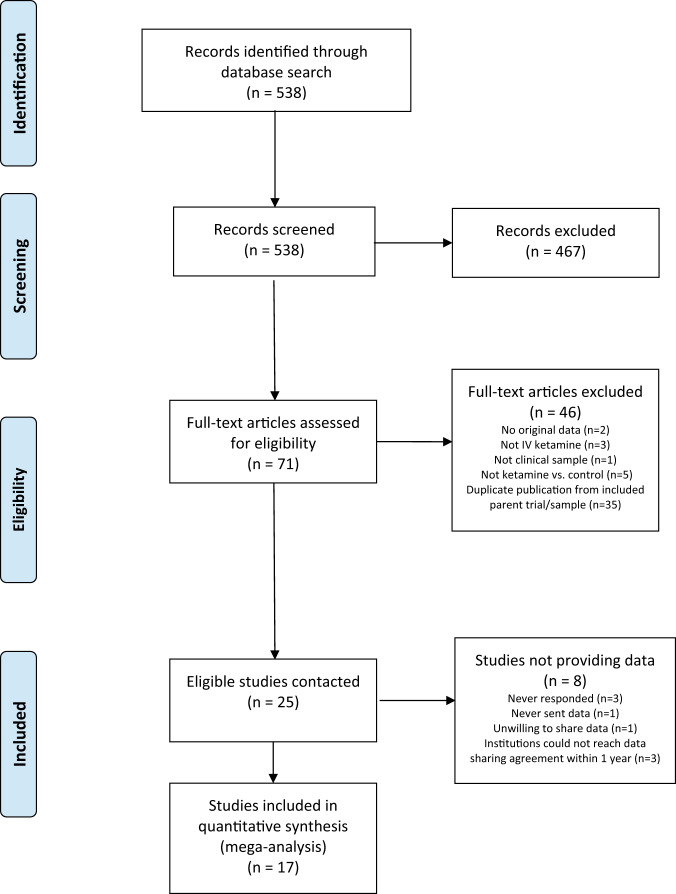
PRISMA flowchart. Number of studies identified, screened for eligibility, and included in final analyses, with tallied reasons for exclusion.

See Fig. 1 for PRISMA flowchart. At least one usable outcome variable was obtained from 68% of eligible studies (17/25; *n* = 809 patients). Of these, a total of *n* = 720 patients received one of the ketamine or control conditions specified for inclusion in meta-analyses. Table 1 presents descriptive characteristics of participating studies; Supplementary-1 presents quality assessments of included studies.

### Main effects

#### Rapid effect

Ketamine was associated with a robust rapid effect on MADRS (% improvement from baseline) approximately 1 day post-infusion [β*(95% CI) = 0.58 (0.44, 0.72); *p* < 0.0001]. This corresponded to a 3-fold increased likelihood of response for ketamine relative to placebo [ketamine—45.5% (*n* = 172/378), control—20.5% (*n* = 68/331); OR (95% CI) = 3.20 (2.27, 4.54); *p* < 0.0001; number-needed-to-treat (NNT) = 4.0] and a 2.5-fold increase in likelihood of rapid remission [ketamine—27.0% (*n* = 102/378), control—13.0% (*n* = 43/331); OR (95% CI) = 2.51 (1.68, 3.79); *p* < 0.0001; NNT = 7.0].

#### Post-rapid effect

Ketamine was associated with a robust, continued, post-rapid effect on MADRS scores approximately 7 days post-infusion [β*(95% CI) = 0.38 (0.23, 0.54); *p* < 0.0001]. This corresponded to nearly a 3-fold increased likelihood of response [ketamine—37.7% (*n* = 119/316), control—18.3% (*n* = 50/273); OR (95% CI) = 2.85 (1.89, 4.36); *p* < 0.0001; number-needed-to-treat (NNT) = 5.2] and a 2.4-fold increase in likelihood of remission approximately 7 days post-infusion [ketamine—25.0% (*n* = 79/316), control—12.1% (*n* = 33/273); OR (95% CI) = 2.40 (1.51, 3.88); *p* = 0.00023; NNT = 7.8].

### Sequential moderators

Of 37 moderators tested sequentially, three significant “Tier 1” moderators were identified pertaining to study-level design features (two that were robust after adjusting for multiple comparisons), and one exploratory “Tier 2” patient-level moderator was significant.

#### Tier 1 moderators

**Fig. 2:**
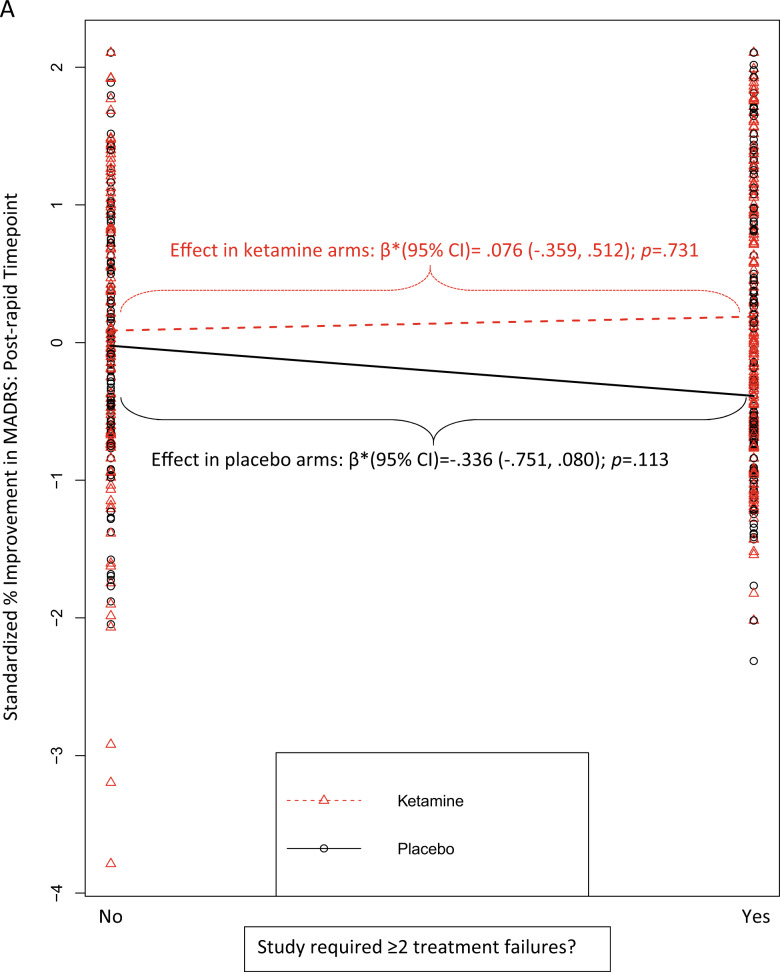

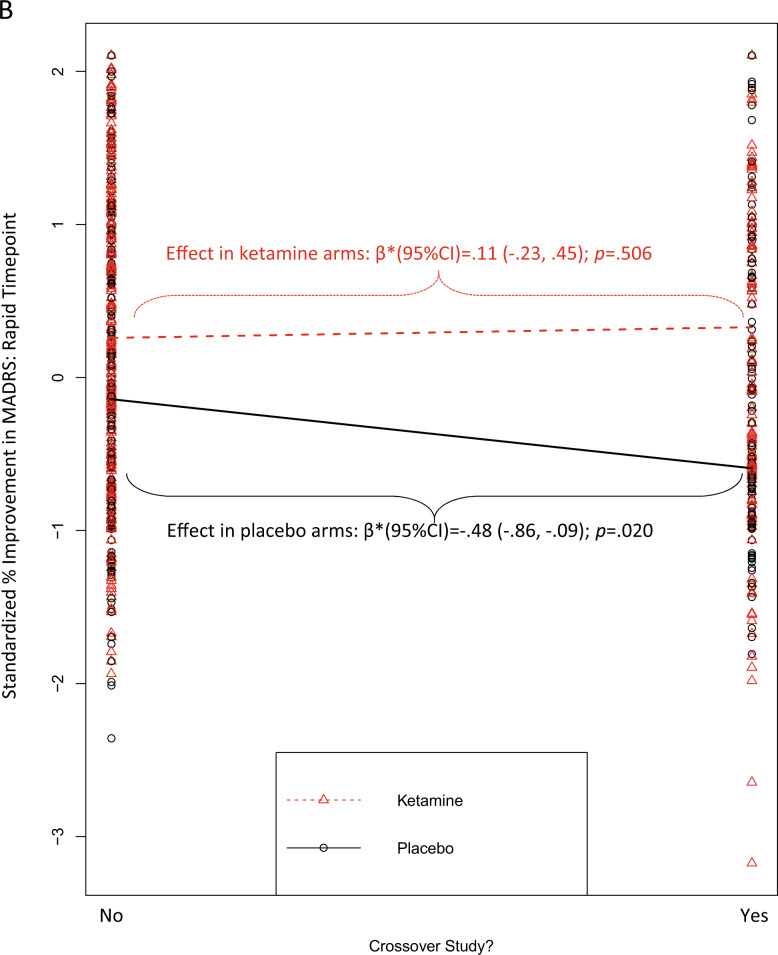

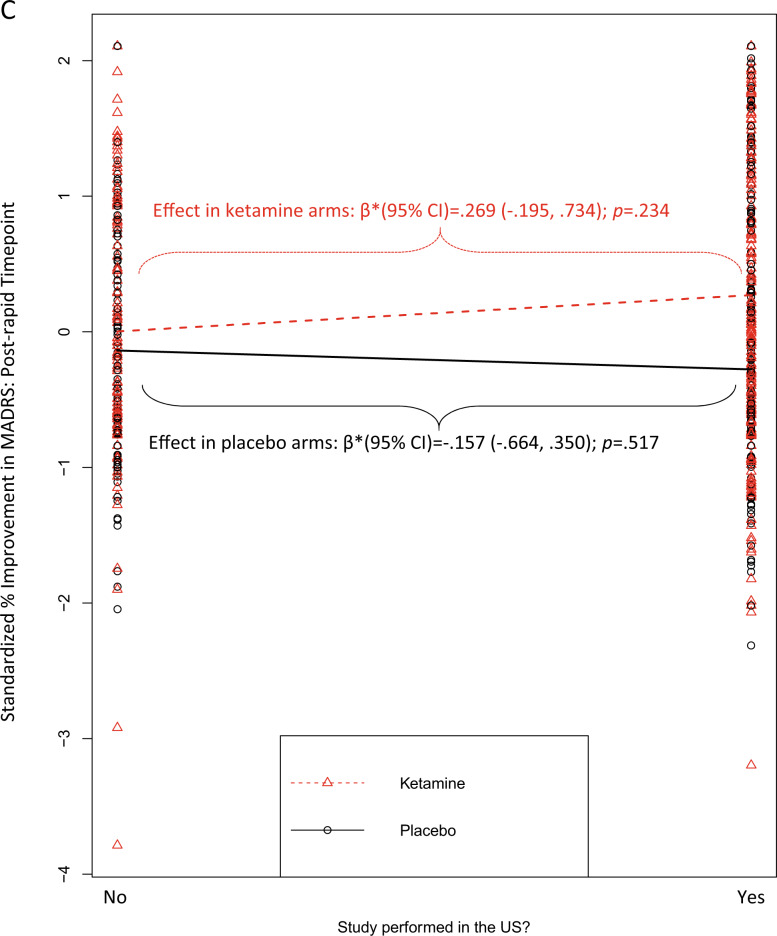
Moderators of the effect of ketamine vs. placebo on standardized % improvement in MADRS scores. In all figures, larger scores on the y-axis = greater improvement from baseline, expressed in standard deviation units relative to the overall sample mean. **A** moderation by study’s eligibility threshold for the number of previous failed, adequate antidepressant medication trials that were required for study enrollment (post-rapid timepoint); **B** moderation by use of a crossover design (rapid timepoint); **C** moderation by study performance in the US (post-rapid timepoint). Regression prediction lines based on models predicting MADRS % improvement from baseline (standardized across the full dataset) at post-infusion (rapid or post-rapid) timepoint with a random effect for study. All individual patient-level datapoints are depicted by red triangles (ketamine-treated patients) or black circles (placebo-treated patients). Statistics overlaid on each figure depict the simple effects of the moderator variable within ketamine-treated patients alone and within placebo-treated patients alone.

The effect of ketamine, relative to placebo, was greater for studies with a higher treatment-resistant depression (TRD) threshold (≥2 failed antidepressant medication [ADM] trials) as a condition of enrollment. The effect for the rapid timepoint outcome [*r* = 0.083; β*(95% CI) = 0.32 (0.04, 0.59);*p*_*unadjusted*_ = 0.023; *p*_*adjusted*_ = 0.207] did not survive multiple comparisons correction, but the effect for the post-rapid timepoint outcome was robust [*r* = 0.108; β*(95% CI) = 0.47 (0.16, 0.77); *p*_*unadjusted*_ = 0.003; *p*_*adjusted*_ = 0.027]. These interaction effects were driven jointly by numerically (but not statistically) larger ketamine responses, combined with numerically (but not statistically) lower placebo responses, in studies enrolling patients with greater treatment resistance (Fig. 2A).

The effect of ketamine relative to placebo was also greater for studies with a crossover design, but only at the rapid timepoint [*r* = 0.132; β*(95% CI) = 0.52 (0.23, 0.81); *p*_*unadjusted*_ = 0.0004; *p*_*adjusted*_ = 0.036; Fig. 2B], and not at the post-rapid timepoint [*r* = 0.041; β*(95% CI) = 0.16 (−0.15, 0.48); *p*_*unadjusted*_ = 0.301; *p*_*adjusted*_ = 1.0]. This interaction effect at the rapid timepoint was driven by a significantly lower placebo response in the trials with a crossover design [within placebo-treated patients: β*(95% CI) = −0.48 (−0.86, −0.09); *p* = 0.020], while the ketamine response in crossover trials was numerically (but not statistically) higher than in parallel-arm studies [within ketamine-treated patients: β* (95% CI) = 0.11 (−0.23, 0.45); *p* = 0.506].

The effect of ketamine, relative to placebo, was also greater for studies completed in the U.S., but only at the post-rapid timepoint, and this did not survive multiple comparisons correction [*r* = 0.089; β*(95% CI) = 0.41 (0.10, 0.72); *p*_*unadjusted*_ = 0.0096; *p*_*adjusted*_ = 0.086]. This pattern was driven jointly by a numerically (but not statistically) lower placebo response and a numerically (but not statistically) higher ketamine response among trials conducted in the U.S. (Fig. 2C).

#### Tier 2 (exploratory) moderators

At the post-rapid timepoint (but not the rapid timepoint), baseline systolic blood pressure moderated response [*r* = 0.106; β*(95% CI) = 0.23 (0.04, 0.42); *p*_*unadjusted*_ = 0.019], such that higher blood pressure at baseline was associated with better post-rapid response to ketamine specifically.

See Supplementary-1 for effect sizes and statistics for all (Tier 1 and Tier 2) individual moderators. Six additional moderators [placebo type (inert vs. psychoactive); marital status; Black race; number of failed trials (coded at the patient level); number of major depressive episodes; BMI] exhibited non-significant trend-level (*p*_*unadjusted*_ < 0.10) moderation effects in at least one analysis.

### Combined moderators

Full findings for all M* analyses are presented in Supplementary-2. Overall, each M* analysis was statistically significant (95% CI did not cross 0), and all M* effect sizes uniformly exceeded the largest effect size observed for any individual moderator above (i.e., *r* = 0.11). However, effect size point estimates (*r;* interpretable as a correlation coefficient) remained small-to-medium (range across all M* analyses: *r* = 0.12-0.29).

M* #2 f provided the maximum differential effect size for both the rapid [*r* (95% CI) = 0.293 (0.175, 0.415)] and post-rapid [*r* (95% CI) = 0.234 (0.118, 0.347)] outcome timepoints. This model utilized data from *n* = 232 patients (7 studies) and included six Tier 1 variables [current MDD diagnosis (present/absent), inpatient (vs. outpatient), age, sex, study done in US, study TRD threshold ≥2] plus BMI, and smoker status (yes/no). For the rapid timepoint (where the effect size was maximal), study-level TRD threshold, MDD diagnosis, country where the study was conducted (US or outside of US), and BMI contributed the largest weights to the combined moderator, such that participants who had greater treatment resistance, had *no* diagnosis of MDD (e.g., had bipolar disorder, PTSD), were enrolled in the US, and had a higher BMI tended to have greater improvement in ketamine relative to placebo. Notably, only one of these variables was significant as an individual moderator, but in combination, the variables provide information regarding participants who may benefit from ketamine, with a small-medium combined effect size.

### Comment

The current analyses were conducted in the largest pooled patient-level dataset of ketamine-treated patients to date, involving patients enrolled in 8 countries (over 4 continents) who were assessed for depression symptoms before and after a single infusion. Results from patient-level data confirmed the robust rapid (app. 1 day post-infusion) and post-rapid (app. 7 days post-infusion) impact of IV ketamine on depression symptoms across a wide range of study designs and patient characteristics. Overall response (peak of 46%) and remission (peak of 27%) rates were comparable to those observed retrospectively in clinical settings [37], but lower than those observed in the earliest published RCTs [38–40], consistent with a waning pattern of effect sizes observed across many disciplines as a field of study matures [41]. Despite variability in patient outcomes, an exhaustive search for moderators of outcome across 37 variables (Table 2) produced very few individual study- or patient-level features that reliably predicted ketamine’s benefit over placebo, suggesting ketamine’s antidepressant impact is highly uniform across heterogeneous patients. Compiling information across multiple variables simultaneously using a validated, data-driven approach [32, 33] yielded several combined moderators, whereby combining study- and patient-level variables enabled the differential impact of ketamine among some patients relative to others to emerge. Nevertheless, effect sizes remained modest (max effect size of *r* = 0.29, a small-medium effect), suggesting limited clinical utility for precision medicine applications.

Despite modest effect sizes, the few significant moderators that were identified have implications for both research design and clinical applications. The observation of stronger effects among studies utilizing a higher threshold of treatment-resistance for study entry (≥2 failed adequate trials of a federal regulatory agency-approved antidepressant medication) suggests that studies will have improved power to detect separation of ketamine from placebo if such eligibility thresholds are used, and further confirms that the current consensus recommendation to conduct a thorough treatment history assessment [4, 28] and consider reserving ketamine treatment for patients who have not responded to previous adequate trials of first-line depression treatments is well warranted—unless an urgent clinical need (e.g., suicidal crisis; marked deterioration in functioning) is present that justifies an initial (and potentially time-limited) course of ketamine. In practice, specialized ketamine clinics may not uniformly uphold this standard, which raises an ethical concern in light of relatively high out-of-pocket expenses to patients [6]. A second study design feature—the use of a crossover design—was also associated with enhanced ketamine efficacy. Of note, the effect of crossover study design cannot be explained by carry-over effects, repeated measurements, or the influence of repeated infusions themselves (e.g., increased functional unblinding), since only data from the first infusion each patient received was included in the present analyses. Patient expectancies, a powerful predictor of response [42], might be differentially impacted in crossover relative to parallel arm studies, given the guarantee of receiving ketamine. Finally, the finding of stronger post-rapid efficacy among U.S. patients, which did not survive multiple comparisons correction, could tentatively be related to cultural features of U.S. patients; features of the U.S. clinical treatment landscape (e.g., private insurance; specific treatment settings and guidelines); and/or study features, including the chronology of data collection [41], with the initial discoveries of ketamine’s antidepressant effects occurring in the U.S. [38–40].

In Tier 1 moderator analyses, which included all patients in the pooled sample, the absence of moderating effects for numerous demographic and clinical features, including age, sex, and unipolar (relative to bipolar) depression, suggests broadly equivalent clinical applicability of ketamine treatment for providing acute relief to heterogeneous adults with depression symptoms. The consistent lack of moderating effects for sex among human patients is important given that such effects have been suggested based on pre-clinical animal models [43, 44]. Likewise, the lack of moderation findings for medication status (presence/absence of concomitant psychiatric medications, as well as number of psychiatric medications) is also notable and relevant in both research and clinical practice.

Similarly, the current analyses did not uphold the reliability of several moderators reported previously in smaller cohorts, such as concurrent benzodiazepine prescriptions [10] and BMI [9]. We leveraged an innovative data-driven “combined moderator” approach to produce optimized weighted combinations of discrete moderator variables, a technique that has been used previously to identify subgroups of patients who will respond beneficially to a treatment, even when each individual moderator, treated in isolation, cannot do so [34–36]. For instance, although BMI moderated outcome only at a trend level in sequential moderator analyses (Supplementary-1), our combined moderator analyses (M* #2 f) for the rapid timepoint suggested that having increased BMI, *in combination* with living in the US, having no diagnosis of MDD (e.g., bipolar disorder, PTSD), and having greater prior treatment resistance, and when simultaneously accounting for information across 6 additional variables (see Supplementary-2, Tier #2 f analyses), did predict differential response to ketamine, to the greatest degree of any of the 8 unique moderator combinations tested within the current analyses. Nevertheless, the maximum effect size remained small by conventional standards (*r* ≤ 0.29), meaning much of the variance in post-ketamine depression was left unexplained. In previous clinical trials where the current combined moderator approach has been applied [34–36], combined moderators have yielded larger effect sizes, reinforcing the conclusion that ketamine’s differential impact on depression was particularly challenging to predict from the current set of moderators—whether tested alone or in combination.

More broadly, the scarcity of moderation findings in the present analyses suggests that information available routinely in clinical settings (i.e., demographic and clinical features) may have limited utility in guiding precision medicine application of ketamine treatment to individual patients. Mechanistic moderators assessing treatment-relevant substrates with more costly and/or invasive methods (e.g., neuroimaging [11–13]; blood tests [15–18]) may be necessary to explain sufficient variance to guide clinical decision-making, but studies of such response markers are few and findings have yet to be replicated. Enhancing the availability and generalizability of such measures in real-world clinical settings may prove an important longer-term goal.

### Limitations

We were constrained by certain aspects of the available published datasets, including predominant use of single infusion designs within randomized trials, which differs from clinical practice in which serial ketamine infusions are the norm [6]; lack of longer-term follow-up data; and a constrained set of moderators available for harmonization across multiple datasets. Several moderators were available only as between-study indicators, which decreases statistical power to detect moderation and fails to fully leverage the pooled patient-level approach. In M* analyses, comparisons of effect sizes across Tiers 2a-g are complicated by the different subsets of patients and studies available for inclusion in each analysis; however, due to small-to-medium overall effect sizes observed consistently across all tiers, the interpretation of moderator findings as having low overall clinical utility is not impacted. Although previous studies suggest that response to a single, first infusion of ketamine is a fairly robust predictor of response to subsequent, serial infusions [45], some [46, 47] (but not all [48]) findings suggest enhanced outcomes can be achieved even among first infusion non-responders through sustained treatment. Our analyses cannot account for this possibility. We did not include trials of the FDA-approved compound intranasal esketamine, given relatively fewer published studies with lower clinical heterogeneity within such studies [49] and relevant proprietary restrictions that impacted the availability of patient-level data when attempting to establish institutional data-sharing agreements. Though this might limit the clinical generalizability of our analyses, off-label IV ketamine use remains widespread, and the need for precision medicine tools is even more pressing in these contexts given that the cost of such treatments predominantly rests with the patient.

At the time of the literature review, no published studies that recruited pediatric/adolescent or geriatric patients could be identified meeting other study eligibility criteria, although positive findings in these age groups have been reported in the interim [50, 51]. Similarly, few studies could be identified in patients with non-primary depressive diagnoses that measured pre- and post-infusion depression with standard outcome measures, and most studies excluded patients with psychiatric, substance, and/or medical comorbidities that are commonly present in real-world clinical patients and urgently require novel treatment approaches, as they confer heightened risk of poor outcomes (e.g., suicidal behaviors; protracted course of illness) [52]. Finally, despite strong international collaboration, the included datasets had high racial and ethnic homogeneity, both within and across studies. Given the transdiagnostic, cross-developmental relevance of depressive symptoms and clinical interest in a broad range of applications for ketamine within psychiatry, recruitment of heterogeneous patient samples with greater real-world representation, diversity, and key comorbidities (e.g., concurrent depression and substance use disorders) is an important goal for future work.

## Conclusions

The efficacy of IV ketamine for both rapid and post-rapid depression reduction was validated in this international pooled patient-level mega-analysis. Although the clinical response to ketamine treatment showed substantial individual differences and room for improvement (46% overall responder rate and 27% remission), the current, comprehensive search for moderators, involving both sequential/univariate and data-driven combined moderator methods, yielded limited capacity to guide clinical decision-making in advance of a first infusion. Given the rapidity of ketamine’s therapeutic onset, a “fast-fail” approach to empirically assess the impact of a time-limited trial of infusions (e.g., between one and three infusions [47]) remains the most accurate method currently available, but in many countries (such as the U.S.), this approach has low accessibility to the vast majority of patients, entailing high out-of-pocket expense and introducing potential concerns regarding risk-to-benefit ratio [5]. Further development of mechanistic measures—particularly those that map onto ketamine’s essential impacts on the brain, yet remain clinically accessible and affordable to perform at pre-infusion baseline—may yield an as-yet unrealized capacity for precision ketamine treatment.

## Supplementary information

Supplement 1

Supplement 2

## Data Availability

Computer code to run all analyses in R (version 3.6.3) is available upon reasonable request made to the corresponding author.
