# Supplementary material for: International pooled patient-level meta-analysis of ketamine infusion for depression: In search of clinical moderators
Source: Mol Psychiatry. 2022 Sep 7;27(12):5096–112. doi: 10.1038/s41380-022-01757-7 (PMC9763119; doi:10.1038/s41380-022-01757-7)
Supplement: Supplementary file 1 — Supplement 1 [file 41380_2022_1757_MOESM1_ESM.docx]

**International Pooled Patient-Level Meta-analysis of Ketamine Infusion for Depression: In Search of Clinical Moderators**

***Supplemental Information #1***

[Supplemental Methods & Materials 2](#_Toc99111163)

[Table S1: Quality Assessment ratings for included studies using Cochrane Collaboration Risk of Bias Tool 2](#_Toc99111164)

[Impact of MADRS estimation applied in subset of studies 3](#_Toc99111165)

[Supplemental Analyses 3](#_Toc99111166)

[Table S2: Regression statistics for all Tier 1 and Tier 2 moderators 3](#_Toc99111167)

[Publication bias assessment 23](#_Toc99111168)

[Supplemental References 24](#_Toc99111169)

# Supplemental Methods & Materials

## Table S1: Quality Assessment ratings for included studies using Cochrane Collaboration Risk of Bias Tool

|  | **Random Sequence Generation** | **Allocation Concealment** | **Blinding of Participants and Staff** | **Blinding of Outcome Assessments** | **Incomplete Outcome Data** |
| --- | --- | --- | --- | --- | --- |
|  | **Low**  n=16/17 (94%) Computerized random generator^L^  n=1/17 (6%) Shuffling cards or envelopes^L^ | **Low**  n=13/17 (77%) Central allocation^L^  n=2/17 (12%) Honest broker provided allocation one-by-one for each patient^L^  n=1/17 (6%) Sequentially numbered drug containers of identical appearance^L^  n=1/17 (6%) Sequentially numbered, opaque, sealed envelopes^L^ | **Low/Moderate**  n=10/17 (59%) Blinding of participants and key study personnel ensured, and unlikely that the blinding could have been broken^L^  n=6/17 (35%) Blinding of participants and key study personnel attempted, but likely that the blinding could have been broken due to functional unblinding (e.g., differing side effect or adverse event profiles)^M^  n=1/17 (6%) Anaesthetist administering ketamine not blinded; everybody else was blinded^M^ | **Low/Moderate**  n=12/17 (71%) Blinding of outcome assessors ensured, and unlikely that the blinding could have been broken^L^  n=4/17 (23%) Blinding of outcome assessors attempted, but likely that the blinding could have been broken due to functional unblinding (e.g., differing side effect profiles)^M^  n=1/17 (6%) Blinding of outcome assessors attempted, but likely that the blinding could have been broken due to accidental access to treatment allocations or other inadvertent information^M^ | **Low**  n=9/17 (53%) No missing outcome data^L^  n=6/17 (35%) Reasons for missing outcome data unlikely to be related to true outcome^L^  n=2/17 (12%) Missing outcome data balanced in numbers across intervention groups, with similar reasons for missing data across groups^L^ |

Note: Assessed via survey responses provided by each contributing study team, tabulated across the 17 studies contributing data.

^L^=low risk per Cochrane Risk of Bias tool; ^M^=potential for moderate risk per Cochrane Risk of Bias tool.

## Impact of MADRS estimation applied in subset of studies

Studies in which participant-level MADRS scores were estimated from HRSD-17 scores (n= 3 studies; n=143 participants) did not differ from those with raw MADRS scores in terms of average MADRS scores at baseline (*p*=.529), rapid (*p*=.677), or post-rapid (*p*=.812) timepoints. Furthermore, there was no significant main or moderation effect for MADRS estimation (as a -, study-level variable) in either the rapid (*p*’s≥.413) or the post-rapid (*p*’s≥.719) models, suggesting outcome data in studies with estimated MADRS scores did not differ systematically from those with raw MADRS scores available.

# Supplemental Analyses

## Table S2: Regression statistics for all Tier 1 and Tier 2 moderators

Note: For prediction of % change (improvement) in MADRS score at both rapid and post-rapid timepoints, “main effects” models display the main effects of treatment and the non-specific prediction effects of the specified variable (across both groups); “model with moderation (interaction) effect” adds the moderator*treatment interaction term to the same regression model.

Green highlighting below marks all moderation (interaction) effects with *p*<.05 (unadjusted); yellow highlighting marks all moderation (interaction) effects with *p*<.10 (unadjusted). AD=Anxiety Disorder.

| **Tier 1 Variables** | **B* estimate** | **lower (95% CI)** | **upper (95% CI)** | **Std.Error** | **DF** | **t-value** | **p-value** |
| --- | --- | --- | --- | --- | --- | --- | --- |
| **Rapid response: main effects model** |  |  |  |  |  |  |  |
| Intercept | -0.5197 | -0.7162 | -0.3232 | 0.1001 | 691 | -5.1924 | 0.0000 |
| Treatment | 0.5807 | 0.4430 | 0.7184 | 0.0701 | 691 | 8.2814 | 0.0000 |
| StudyPlaceboType | 0.3364 | 0.0802 | 0.5927 | 0.1202 | 15 | 2.7983 | 0.0135 |
| **Rapid response: model with moderation (interaction) effect** | | |  |  |  |  |  |
| Intercept | -0.6016 | -0.8201 | -0.3831 | 0.1113 | 690 | -5.4066 | 0.0000 |
| Treatment | 0.7448 | 0.5092 | 0.9804 | 0.1200 | 690 | 6.2075 | 0.0000 |
| StudyPlaceboType | 0.4645 | 0.1613 | 0.7677 | 0.1423 | 15 | 3.2651 | 0.0052 |
| Treatment* StudyPlaceboType | -0.2489 | -0.5390 | 0.0412 | 0.1478 | 690 | -1.6844 | 0.0926 |
| **Post-rapid response: main effects model** | |  |  |  |  |  |  |
| Intercept | -0.3368 | -0.6214 | -0.0522 | 0.1449 | 572 | -2.3246 | 0.0204 |
| Treatment | 0.3822 | 0.2302 | 0.5342 | 0.0774 | 572 | 4.9390 | 0.0000 |

| StudyPlaceboType | 0.2084 | -0.1895 | 0.6063 | 0.1855 | 14 | 1.1232 | 0.2802 |
| --- | --- | --- | --- | --- | --- | --- | --- |
| **Post-rapid response: model with moderation (interaction) effect** | | | |  |  |  |  |
| Intercept | -0.2468 | -0.5487 | 0.0552 | 0.1537 | 571 | -1.6052 | 0.1090 |
| Treatment | 0.1983 | -0.0436 | 0.4402 | 0.1231 | 571 | 1.6104 | 0.1079 |
| StudyPlaceboType | 0.0535 | -0.3846 | 0.4917 | 0.2043 | 14 | 0.2620 | 0.7971 |
| Treatment* StudyPlaceboType | 0.3027 | -0.0077 | 0.6131 | 0.1580 | 571 | 1.9154 | 0.0559 |
| **Rapid response: main effects model** |  |  |  |  |  |  |  |
| Intercept | -0.2404 | -0.4275 | -0.0534 | 0.0953 | 691 | -2.5239 | 0.0118 |
| Treatment | 0.5787 | 0.4408 | 0.7167 | 0.0703 | 691 | 8.2363 | 0.0000 |
| CrossoverStudy | -0.1876 | -0.4819 | 0.1068 | 0.1381 | 15 | -1.3581 | 0.1945 |
| **Rapid response: model with moderation (interaction) effect** | | |  |  |  |  |  |
| Intercept | -0.1415 | -0.3348 | 0.0519 | 0.0985 | 690 | -1.4363 | 0.1514 |
| Treatment | 0.4004 | 0.2317 | 0.5691 | 0.0859 | 690 | 4.6597 | 0.0000 |
| CrossoverStudy | -0.4517 | -0.7838 | -0.1195 | 0.1558 | 15 | -2.8985 | 0.0110 |
| Treatment* CrossoverStudy | 0.5215 | 0.2330 | 0.8099 | 0.1469 | 690 | 3.5495 | 0.0004 |
| **Post-rapid response: main effects model** | |  |  |  |  |  |  |
| Intercept | -0.0044 | -0.2087 | 0.1999 | 0.1040 | 572 | -0.0420 | 0.9665 |
| Treatment | 0.3766 | 0.2248 | 0.5283 | 0.0772 | 572 | 4.8747 | 0.0000 |
| CrossoverStudy | -0.4937 | -0.8049 | -0.1824 | 0.1451 | 14 | -3.4017 | 0.0043 |
| **Post-rapid response: model with moderation (interaction) effect** | | | |  |  |  |  |
| Intercept | 0.0314 | -0.1846 | 0.2474 | 0.1100 | 571 | 0.2854 | 0.7755 |
| Treatment | 0.3127 | 0.1186 | 0.5069 | 0.0988 | 571 | 3.1638 | 0.0016 |
| CrossoverStudy | -0.5768 | -0.9338 | -0.2198 | 0.1664 | 14 | -3.4656 | 0.0038 |
| Treatment* CrossoverStudy | 0.1640 | -0.1471 | 0.4751 | 0.1584 | 571 | 1.0353 | 0.3010 |
| **Rapid response: main effects model** |  |  |  |  |  |  |  |
| Intercept | -0.2521 | -0.5050 | 0.0007 | 0.1288 | 690 | -1.9582 | 0.0506 |
| Treatment | 0.5832 | 0.4452 | 0.7212 | 0.0703 | 690 | 8.2959 | 0.0000 |
| PrimaryDxIsMDD | -0.0832 | -0.3422 | 0.1758 | 0.1319 | 690 | -0.6310 | 0.5282 |
| **Rapid response: model with moderation (interaction) effect** | | |  |  |  |  |  |
| Intercept | -0.2586 | -0.5426 | 0.0255 | 0.1447 | 689 | -1.7873 | 0.0743 |
| Treatment | 0.5969 | 0.2890 | 0.9048 | 0.1568 | 689 | 3.8066 | 0.0002 |
| PrimaryDxIsMDD | -0.0750 | -0.3826 | 0.2326 | 0.1567 | 689 | -0.4788 | 0.6322 |
| Treatment* PrimaryDxIsMDD | -0.0172 | -0.3616 | 0.3273 | 0.1755 | 689 | -0.0978 | 0.9221 |
| **Post-rapid response: main effects model** | |  |  |  |  |  |  |
| Intercept | -0.1973 | -0.5456 | 0.1509 | 0.1773 | 571 | -1.1130 | 0.2662 |
| Treatment | 0.3842 | 0.2321 | 0.5363 | 0.0774 | 571 | 4.9611 | 0.0000 |
| PrimaryDxIsMDD | -0.0284 | -0.3956 | 0.3388 | 0.1870 | 571 | -0.1517 | 0.8795 |
| **Post-rapid response: model with moderation (interaction) effect** | | | |  |  |  |  |
| Intercept | -0.2433 | -0.6188 | 0.1323 | 0.1912 | 570 | -1.2722 | 0.2038 |
| Treatment | 0.4857 | 0.1430 | 0.8284 | 0.1745 | 570 | 2.7835 | 0.0056 |
| PrimaryDxIsMDD | 0.0307 | -0.3784 | 0.4397 | 0.2083 | 570 | 0.1473 | 0.8829 |
| Treatment* PrimaryDxIsMDD | -0.1265 | -0.5090 | 0.2561 | 0.1948 | 570 | -0.6493 | 0.5164 |
| **Rapid response: main effects model** |  |  |  |  |  |  |  |
| Intercept | -0.3033 | -0.4584 | -0.1482 | 0.0790 | 690 | -3.8394 | 0.0001 |
| Treatment | 0.5808 | 0.4428 | 0.7188 | 0.0703 | 690 | 8.2638 | 0.0000 |
| PrimaryDxIsBipolar | -0.1361 | -0.4523 | 0.1800 | 0.1610 | 690 | -0.8454 | 0.3982 |
| **Rapid response: model with moderation (interaction) effect** | | |  |  |  |  |  |
| Intercept | -0.2924 | -0.4494 | -0.1354 | 0.0799 | 689 | -3.6574 | 0.0003 |
| Treatment | 0.5607 | 0.4158 | 0.7056 | 0.0738 | 689 | 7.5967 | 0.0000 |
| PrimaryDxIsBipolar | -0.2377 | -0.6245 | 0.1490 | 0.1970 | 689 | -1.2068 | 0.2279 |
| Treatment* PrimaryDxIsBipolar | 0.2170 | -0.2586 | 0.6925 | 0.2422 | 689 | 0.8958 | 0.3707 |
| **Post-rapid response: main effects model** | |  |  |  |  |  |  |
| Intercept | -0.2096 | -0.4138 | -0.0054 | 0.1040 | 571 | -2.0159 | 0.0443 |
| Treatment | 0.3834 | 0.2313 | 0.5354 | 0.0774 | 571 | 4.9511 | 0.0000 |
| PrimaryDxIsBipolar | -0.1082 | -0.5889 | 0.3724 | 0.2447 | 571 | -0.4423 | 0.6585 |
| **Post-rapid response: model with moderation (interaction) effect** | | | |  |  |  |  |
| Intercept | -0.2033 | -0.4090 | 0.0023 | 0.1047 | 570 | -1.9420 | 0.0526 |
| Treatment | 0.3726 | 0.2146 | 0.5306 | 0.0804 | 570 | 4.6319 | 0.0000 |
| PrimaryDxIsBipolar | -0.1754 | -0.7245 | 0.3738 | 0.2796 | 570 | -0.6272 | 0.5307 |
| Treatment* PrimaryDxIsBipolar | 0.1487 | -0.4395 | 0.7370 | 0.2995 | 570 | 0.4965 | 0.6197 |
| **Rapid response: main effects model** |  |  |  |  |  |  |  |
| Intercept | -0.3775 | -0.6428 | -0.1121 | 0.1352 | 690 | -2.7929 | 0.0054 |
| Treatment | 0.5802 | 0.4421 | 0.7182 | 0.0703 | 690 | 8.2505 | 0.0000 |
| MDD | 0.0717 | -0.1823 | 0.3256 | 0.1294 | 690 | 0.5539 | 0.5798 |
| **Rapid response: model with moderation (interaction) effect** | | |  |  |  |  |  |
| Intercept | -0.4036 | -0.7141 | -0.0932 | 0.1581 | 689 | -2.5528 | 0.0109 |
| Treatment | 0.6379 | 0.2570 | 1.0187 | 0.1940 | 689 | 3.2885 | 0.0011 |
| MDD | 0.1026 | -0.2149 | 0.4200 | 0.1617 | 689 | 0.6345 | 0.5260 |
| Treatment* MDD | -0.0664 | -0.4751 | 0.3423 | 0.2082 | 689 | -0.3192 | 0.7497 |
| **Post-rapid response: main effects model** | |  |  |  |  |  |  |
| Intercept | -0.2213 | -0.5612 | 0.1185 | 0.1730 | 571 | -1.2792 | 0.2013 |
| Treatment | 0.3840 | 0.2318 | 0.5362 | 0.0775 | 571 | 4.9562 | 0.0000 |
| MDD | 0.0024 | -0.3198 | 0.3245 | 0.1640 | 571 | 0.0143 | 0.9886 |
| **Post-rapid response: model with moderation (interaction) effect** | | | |  |  |  |  |
| Intercept | -0.2876 | -0.6757 | 0.1004 | 0.1975 | 570 | -1.4561 | 0.1459 |
| Treatment | 0.5319 | 0.0881 | 0.9756 | 0.2259 | 570 | 2.3542 | 0.0189 |
| MDD | 0.0800 | -0.3096 | 0.4695 | 0.1983 | 570 | 0.4032 | 0.6869 |
| Treatment* MDD | -0.1676 | -0.6401 | 0.3049 | 0.2406 | 570 | -0.6969 | 0.4862 |
| **Rapid response: main effects model** |  |  |  |  |  |  |  |
| Intercept | -0.3160 | -0.4697 | -0.1622 | 0.0783 | 688 | -4.0354 | 0.0001 |
| Treatment | 0.5814 | 0.4430 | 0.7198 | 0.0705 | 688 | 8.2481 | 0.0000 |
| Age | 0.0159 | -0.0591 | 0.0909 | 0.0382 | 688 | 0.4162 | 0.6774 |
| **Rapid response: model with moderation (interaction) effect** | | |  |  |  |  |  |
| Intercept | -0.3167 | -0.4701 | -0.1632 | 0.0782 | 687 | -4.0518 | 0.0001 |
| Treatment | 0.5821 | 0.4436 | 0.7206 | 0.0706 | 687 | 8.2502 | 0.0000 |
| Age | 0.0012 | -0.1066 | 0.1091 | 0.0549 | 687 | 0.0224 | 0.9821 |
| Treatment* Age | 0.0263 | -0.1120 | 0.1646 | 0.0704 | 687 | 0.3733 | 0.7090 |
| **Post-rapid response: main effects model** | |  |  |  |  |  |  |
| Intercept | -0.2147 | -0.4080 | -0.0215 | 0.0984 | 571 | -2.1823 | 0.0295 |
| Treatment | 0.3767 | 0.2251 | 0.5283 | 0.0772 | 571 | 4.8809 | 0.0000 |
| Age | 0.1005 | 0.0169 | 0.1840 | 0.0425 | 571 | 2.3626 | 0.0185 |
| **Post-rapid response: model with moderation (interaction) effect** | | | |  |  |  |  |
| Intercept | -0.2159 | -0.4087 | -0.0232 | 0.0981 | 570 | -2.2006 | 0.0282 |
| Treatment | 0.3758 | 0.2243 | 0.5273 | 0.0771 | 570 | 4.8717 | 0.0000 |
| Age | 0.0418 | -0.0782 | 0.1617 | 0.0611 | 570 | 0.6838 | 0.4944 |
| Treatment* Age | 0.1020 | -0.0475 | 0.2515 | 0.0761 | 570 | 1.3395 | 0.1809 |
| **Rapid response: main effects model** |  |  |  |  |  |  |  |
| Intercept | -0.3543 | -0.5274 | -0.1812 | 0.0882 | 687 | -4.0182 | 0.0001 |
| Treatment | 0.5802 | 0.4417 | 0.7186 | 0.0705 | 687 | 8.2262 | 0.0000 |
| Female | 0.0692 | -0.0722 | 0.2106 | 0.0720 | 687 | 0.9607 | 0.3371 |
| **Rapid response: model with moderation (interaction) effect** | | |  |  |  |  |  |
| Intercept | -0.3324 | -0.5215 | -0.1432 | 0.0964 | 686 | -3.4491 | 0.0006 |
| Treatment | 0.5378 | 0.3339 | 0.7418 | 0.1039 | 686 | 5.1774 | 0.0000 |
| Female | 0.0274 | -0.1767 | 0.2314 | 0.1039 | 686 | 0.2635 | 0.7923 |
| Treatment* Female | 0.0789 | -0.1995 | 0.3572 | 0.1418 | 686 | 0.5564 | 0.5781 |
| **Post-rapid response: main effects model** | |  |  |  |  |  |  |
| Intercept | -0.2604 | -0.4780 | -0.0428 | 0.1108 | 570 | -2.3508 | 0.0191 |
| Treatment | 0.3750 | 0.2232 | 0.5268 | 0.0773 | 570 | 4.8520 | 0.0000 |
| Female | 0.0779 | -0.0778 | 0.2337 | 0.0793 | 570 | 0.9832 | 0.3259 |
| **Post-rapid response: model with moderation (interaction) effect** | | | |  |  |  |  |
| Intercept | -0.3033 | -0.5359 | -0.0707 | 0.1184 | 569 | -2.5614 | 0.0107 |
| Treatment | 0.4585 | 0.2376 | 0.6793 | 0.1124 | 569 | 4.0772 | 0.0001 |
| Female | 0.1613 | -0.0622 | 0.3847 | 0.1138 | 569 | 1.4177 | 0.1568 |
| Treatment* Female | -0.1576 | -0.4606 | 0.1454 | 0.1543 | 569 | -1.0217 | 0.3073 |
| **Rapid response: main effects model** |  |  |  |  |  |  |  |
| Intercept | -0.4383 | -0.6570 | -0.2197 | 0.1113 | 691 | -3.9366 | 0.0001 |
| Treatment | 0.5810 | 0.4431 | 0.7189 | 0.0702 | 691 | 8.2735 | 0.0000 |
| USStudy | 0.2028 | -0.0867 | 0.4923 | 0.1358 | 15 | 1.4933 | 0.1561 |
| **Rapid response: model with moderation (interaction) effect** | | |  |  |  |  |  |
| Intercept | -0.3828 | -0.6220 | -0.1437 | 0.1218 | 690 | -3.1426 | 0.0017 |
| Treatment | 0.4716 | 0.2358 | 0.7075 | 0.1201 | 690 | 3.9261 | 0.0001 |
| USStudy | 0.1168 | -0.2156 | 0.4492 | 0.1560 | 15 | 0.7489 | 0.4655 |
| Treatment* USStudy | 0.1661 | -0.1246 | 0.4568 | 0.1480 | 690 | 1.1221 | 0.2622 |
| **Post-rapid response: main effects model** | |  |  |  |  |  |  |
| Intercept | -0.2586 | -0.5525 | 0.0352 | 0.1496 | 572 | -1.7286 | 0.0844 |
| Treatment | 0.3833 | 0.2312 | 0.5353 | 0.0774 | 572 | 4.9518 | 0.0000 |
| USStudy | 0.0699 | -0.3420 | 0.4818 | 0.1920 | 14 | 0.3641 | 0.7212 |
| **Post-rapid response: model with moderation (interaction) effect** | | | |  |  |  |  |
| Intercept | -0.1381 | -0.4474 | 0.1712 | 0.1575 | 571 | -0.8771 | 0.3808 |
| Treatment | 0.1408 | -0.0967 | 0.3784 | 0.1210 | 571 | 1.1644 | 0.2448 |
| USStudy | -0.1399 | -0.5893 | 0.3094 | 0.2095 | 14 | -0.6679 | 0.5150 |
| Treatment* USStudy | 0.4074 | 0.0993 | 0.7155 | 0.1569 | 571 | 2.5973 | 0.0096 |
| **Rapid response: main effects model** |  |  |  |  |  |  |  |
| Intercept | -0.2057 | -0.3738 | -0.0377 | 0.0856 | 690 | -2.4037 | 0.0165 |
| Treatment | 0.5802 | 0.4425 | 0.7179 | 0.0701 | 690 | 8.2712 | 0.0000 |
| Inpatient | -0.3017 | -0.5471 | -0.0563 | 0.1250 | 690 | -2.4143 | 0.0160 |
| **Rapid response: model with moderation (interaction) effect** | | |  |  |  |  |  |
| Intercept | -0.1814 | -0.3591 | -0.0038 | 0.0905 | 689 | -2.0054 | 0.0453 |
| Treatment | 0.5364 | 0.3634 | 0.7094 | 0.0881 | 689 | 6.0879 | 0.0000 |
| Inpatient | -0.3630 | -0.6486 | -0.0774 | 0.1454 | 689 | -2.4959 | 0.0128 |
| Treatment* Inpatient | 0.1199 | -0.1667 | 0.4065 | 0.1460 | 689 | 0.8214 | 0.4117 |
| **Post-rapid response: main effects model** | |  |  |  |  |  |  |
| Intercept | -0.1766 | -0.4043 | 0.0511 | 0.1159 | 571 | -1.5230 | 0.1283 |
| Treatment | 0.3838 | 0.2318 | 0.5358 | 0.0774 | 571 | 4.9579 | 0.0000 |
| Inpatient | -0.1329 | -0.4860 | 0.2201 | 0.1798 | 571 | -0.7396 | 0.4599 |
| **Post-rapid response: model with moderation (interaction) effect** | | | |  |  |  |  |
| Intercept | -0.1752 | -0.4086 | 0.0582 | 0.1188 | 570 | -1.4742 | 0.1410 |
| Treatment | 0.3814 | 0.2058 | 0.5571 | 0.0894 | 570 | 4.2653 | 0.0000 |
| Inpatient | -0.1378 | -0.5335 | 0.2579 | 0.2015 | 570 | -0.6839 | 0.4943 |
| Treatment* Inpatient | 0.0095 | -0.3439 | 0.3629 | 0.1799 | 570 | 0.0527 | 0.9580 |
| **Rapid response: main effects model** |  |  |  |  |  |  |  |
| Intercept | -0.3567 | -0.5593 | -0.1540 | 0.1032 | 690 | -3.4559 | 0.0006 |
| Treatment | 0.5801 | 0.4422 | 0.7181 | 0.0703 | 690 | 8.2552 | 0.0000 |
| StudyTRDThreshOver2 | 0.0849 | -0.1878 | 0.3577 | 0.1389 | 690 | 0.6113 | 0.5412 |
| **Rapid response: model with moderation (interaction) effect** | | |  |  |  |  |  |
| Intercept | -0.2771 | -0.4918 | -0.0624 | 0.1093 | 689 | -2.5345 | 0.0115 |
| Treatment | 0.4201 | 0.2253 | 0.6149 | 0.0992 | 689 | 4.2338 | 0.0000 |
| StudyTRDThreshOver2 | -0.0829 | -0.3929 | 0.2270 | 0.1579 | 689 | -0.5253 | 0.5995 |
| Treatment* StudyTRDThreshOver2 | 0.3189 | 0.0438 | 0.5940 | 0.1401 | 689 | 2.2758 | 0.0232 |
| **Post-rapid response: main effects model** | |  |  |  |  |  |  |
| Intercept | -0.1563 | -0.4282 | 0.1156 | 0.1384 | 571 | -1.1290 | 0.2594 |
| Treatment | 0.3854 | 0.2334 | 0.5374 | 0.0774 | 571 | 4.9789 | 0.0000 |
| StudyTRDThreshOver2 | -0.1249 | -0.4857 | 0.2358 | 0.1837 | 571 | -0.6802 | 0.4967 |
| **Post-rapid response: model with moderation (interaction) effect** | | | |  |  |  |  |
| Intercept | -0.0234 | -0.3115 | 0.2647 | 0.1467 | 570 | -0.1593 | 0.8735 |
| Treatment | 0.1106 | -0.1251 | 0.3463 | 0.1200 | 570 | 0.9217 | 0.3571 |
| StudyTRDThreshOver2 | -0.3647 | -0.7619 | 0.0325 | 0.2022 | 570 | -1.8036 | 0.0718 |
| Treatment* StudyTRDThreshOver2 | 0.4656 | 0.1586 | 0.7725 | 0.1563 | 570 | 2.9793 | 0.0030 |
|  |  |  |  |  |  |  |  |
| **Tier 2 Variables** | **B* estimate** | **lower (95% CI)** | **upper (95% CI)** | **Std.Error** | **DF** | **t-value** | **p-value** |
| **Rapid response: main effects model** |  |  |  |  |  |  |  |
| Intercept | -0.2971 | -0.4813 | -0.1130 | 0.0937 | 500 | -3.1699 | 0.0016 |
| Treatment | 0.5638 | 0.4052 | 0.7224 | 0.0807 | 500 | 6.9854 | 0.0000 |
| PTSD | 0.1667 | -0.0747 | 0.4081 | 0.1229 | 500 | 1.3569 | 0.1754 |
| **Rapid response: model with moderation (interaction) effect** | | |  |  |  |  |  |
| Intercept | -0.3311 | -0.5210 | -0.1412 | 0.0967 | 499 | -3.4256 | 0.0007 |
| Treatment | 0.6273 | 0.4469 | 0.8078 | 0.0918 | 499 | 6.8302 | 0.0000 |
| PTSD | 0.3064 | -0.0009 | 0.6136 | 0.1564 | 499 | 1.9591 | 0.0507 |
| Treatment* PTSD | -0.2760 | -0.6513 | 0.0994 | 0.1910 | 499 | -1.4446 | 0.1492 |
| **Post-rapid response: main effects model** | |  |  |  |  |  |  |
| Intercept | -0.2389 | -0.5120 | 0.0342 | 0.1389 | 393 | -1.7197 | 0.0863 |
| Treatment | 0.4783 | 0.3025 | 0.6542 | 0.0894 | 393 | 5.3478 | 0.0000 |
| PTSD | 0.0467 | -0.2654 | 0.3588 | 0.1587 | 393 | 0.2941 | 0.7688 |
| **Post-rapid response: model with moderation (interaction) effect** | | | |  |  |  |  |
| Intercept | -0.2353 | -0.5136 | 0.0430 | 0.1416 | 392 | -1.6620 | 0.0973 |
| Treatment | 0.4716 | 0.2696 | 0.6735 | 0.1027 | 392 | 4.5909 | 0.0000 |
| PTSD | 0.0330 | -0.3377 | 0.4037 | 0.1886 | 392 | 0.1752 | 0.8610 |
| Treatment* PTSD | 0.0279 | -0.3804 | 0.4363 | 0.2077 | 392 | 0.1345 | 0.8931 |
| **Rapid response: main effects model** |  |  |  |  |  |  |  |
| Intercept | -0.3422 | -0.5565 | -0.1278 | 0.1091 | 499 | -3.1357 | 0.0018 |
| Treatment | 0.5589 | 0.4010 | 0.7169 | 0.0804 | 499 | 6.9540 | 0.0000 |
| AnyAD | 0.1481 | -0.0276 | 0.3239 | 0.0894 | 499 | 1.6563 | 0.0983 |
| **Rapid response: model with moderation (interaction) effect** | | |  |  |  |  |  |
| Intercept | -0.3285 | -0.5640 | -0.0930 | 0.1199 | 498 | -2.7405 | 0.0064 |
| Treatment | 0.5343 | 0.2981 | 0.7705 | 0.1202 | 498 | 4.4442 | 0.0000 |
| AnyAD | 0.1244 | -0.1195 | 0.3683 | 0.1241 | 498 | 1.0023 | 0.3167 |
| Treatment* AnyAD | 0.0445 | -0.2723 | 0.3613 | 0.1613 | 498 | 0.2760 | 0.7827 |
| **Post-rapid response: main effects model** | |  |  |  |  |  |  |
| Intercept | -0.2573 | -0.5459 | 0.0312 | 0.1468 | 392 | -1.7532 | 0.0804 |
| Treatment | 0.4818 | 0.3055 | 0.6581 | 0.0897 | 392 | 5.3733 | 0.0000 |
| AnyAD | 0.0509 | -0.1535 | 0.2552 | 0.1040 | 392 | 0.4893 | 0.6249 |
| **Post-rapid response: model with moderation (interaction) effect** | | | |  |  |  |  |
| Intercept | -0.2834 | -0.5947 | 0.0279 | 0.1583 | 391 | -1.7901 | 0.0742 |
| Treatment | 0.5272 | 0.2594 | 0.7950 | 0.1362 | 391 | 3.8705 | 0.0001 |
| AnyAD | 0.0951 | -0.1881 | 0.3783 | 0.1440 | 391 | 0.6600 | 0.5096 |
| Treatment* AnyAD | -0.0800 | -0.4346 | 0.2746 | 0.1804 | 391 | -0.4435 | 0.6577 |
| **Rapid response: main effects model** |  |  |  |  |  |  |  |
| Intercept | -0.3414 | -0.5350 | -0.1478 | 0.0985 | 449 | -3.4662 | 0.0006 |
| Treatment | 0.6263 | 0.4620 | 0.7905 | 0.0836 | 449 | 7.4950 | 0.0000 |
| GAD | 0.0308 | -0.1821 | 0.2437 | 0.1083 | 449 | 0.2842 | 0.7764 |
| **Rapid response: model with moderation (interaction) effect** | | |  |  |  |  |  |
| Intercept | -0.3426 | -0.5419 | -0.1434 | 0.1014 | 448 | -3.3793 | 0.0008 |
| Treatment | 0.6285 | 0.4431 | 0.8138 | 0.0943 | 448 | 6.6637 | 0.0000 |
| GAD | 0.0358 | -0.2539 | 0.3256 | 0.1474 | 448 | 0.2430 | 0.8081 |
| Treatment* GAD | -0.0102 | -0.4081 | 0.3877 | 0.2025 | 448 | -0.0505 | 0.9597 |
| **Post-rapid response: main effects model** | |  |  |  |  |  |  |
| Intercept | -0.3270 | -0.5646 | -0.0893 | 0.1208 | 341 | -2.7062 | 0.0071 |
| Treatment | 0.4508 | 0.2639 | 0.6377 | 0.0950 | 341 | 4.7448 | 0.0000 |
| GAD | 0.0388 | -0.1880 | 0.2656 | 0.1153 | 341 | 0.3365 | 0.7367 |
| **Post-rapid response: model with moderation (interaction) effect** | | | |  |  |  |  |
| Intercept | -0.3509 | -0.5964 | -0.1054 | 0.1248 | 340 | -2.8112 | 0.0052 |
| Treatment | 0.4938 | 0.2774 | 0.7102 | 0.1100 | 340 | 4.4883 | 0.0000 |
| GAD | 0.1247 | -0.1896 | 0.4389 | 0.1598 | 340 | 0.7802 | 0.4358 |
| Treatment* GAD | -0.1676 | -0.5921 | 0.2569 | 0.2158 | 340 | -0.7766 | 0.4379 |
| **Rapid response: main effects model** |  |  |  |  |  |  |  |
| Intercept | -0.3400 | -0.5558 | -0.1241 | 0.1098 | 403 | -3.0964 | 0.0021 |
| Treatment | 0.6108 | 0.4366 | 0.7850 | 0.0886 | 403 | 6.8935 | 0.0000 |
| SAD | 0.0634 | -0.1499 | 0.2767 | 0.1085 | 403 | 0.5845 | 0.5592 |
| **Rapid response: model with moderation (interaction) effect** | | |  |  |  |  |  |
| Intercept | -0.3276 | -0.5499 | -0.1053 | 0.1131 | 402 | -2.8974 | 0.0040 |
| Treatment | 0.5888 | 0.3895 | 0.7880 | 0.1014 | 402 | 5.8084 | 0.0000 |
| SAD | 0.0123 | -0.2961 | 0.3208 | 0.1569 | 402 | 0.0785 | 0.9375 |
| Treatment* SAD | 0.0938 | -0.3155 | 0.5031 | 0.2082 | 402 | 0.4506 | 0.6525 |
| **Post-rapid response: main effects model** | |  |  |  |  |  |  |
| Intercept | -0.3004 | -0.5636 | -0.0371 | 0.1338 | 295 | -2.2456 | 0.0255 |
| Treatment | 0.4559 | 0.2476 | 0.6642 | 0.1058 | 295 | 4.3069 | 0.0000 |
| SAD | 0.0126 | -0.2382 | 0.2634 | 0.1274 | 295 | 0.0987 | 0.9214 |
| **Post-rapid response: model with moderation (interaction) effect** | | | |  |  |  |  |
| Intercept | -0.3434 | -0.6163 | -0.0704 | 0.1387 | 294 | -2.4760 | 0.0138 |
| Treatment | 0.5318 | 0.2921 | 0.7714 | 0.1218 | 294 | 4.3668 | 0.0000 |
| SAD | 0.1819 | -0.1822 | 0.5460 | 0.1850 | 294 | 0.9831 | 0.3264 |
| Treatment* SAD | -0.3046 | -0.7808 | 0.1716 | 0.2420 | 294 | -1.2588 | 0.2091 |
| **Rapid response: main effects model** |  |  |  |  |  |  |  |
| Intercept | -0.3271 | -0.5345 | -0.1196 | 0.1055 | 402 | -3.0996 | 0.0021 |
| Treatment | 0.6232 | 0.4471 | 0.7993 | 0.0896 | 402 | 6.9555 | 0.0000 |
| OCD | 0.0475 | -0.3852 | 0.4802 | 0.2201 | 402 | 0.2159 | 0.8292 |
| **Rapid response: model with moderation (interaction) effect** | | |  |  |  |  |  |
| Intercept | -0.3251 | -0.5330 | -0.1172 | 0.1057 | 401 | -3.0745 | 0.0023 |
| Treatment | 0.6192 | 0.4394 | 0.7991 | 0.0915 | 401 | 6.7685 | 0.0000 |
| OCD | 0.0121 | -0.5205 | 0.5448 | 0.2710 | 401 | 0.0448 | 0.9643 |
| Treatment* OCD | 0.1032 | -0.8061 | 1.0125 | 0.4625 | 401 | 0.2232 | 0.8235 |
| **Post-rapid response: main effects model** | |  |  |  |  |  |  |
| Intercept | -0.2822 | -0.5388 | -0.0257 | 0.1304 | 294 | -2.1648 | 0.0312 |
| Treatment | 0.4507 | 0.2425 | 0.6589 | 0.1058 | 294 | 4.2604 | 0.0000 |
| OCD | -0.3114 | -0.8097 | 0.1869 | 0.2532 | 294 | -1.2299 | 0.2197 |
| **Post-rapid response: model with moderation (interaction) effect** | | | |  |  |  |  |
| Intercept | -0.2875 | -0.5447 | -0.0303 | 0.1307 | 293 | -2.1997 | 0.0286 |
| Treatment | 0.4601 | 0.2467 | 0.6736 | 0.1085 | 293 | 4.2427 | 0.0000 |
| OCD | -0.2225 | -0.8841 | 0.4391 | 0.3361 | 293 | -0.6619 | 0.5086 |
| Treatment* OCD | -0.2038 | -1.1989 | 0.7913 | 0.5056 | 293 | -0.4031 | 0.6872 |
| **Rapid response: main effects model** |  |  |  |  |  |  |  |
| Intercept | -0.3324 | -0.5411 | -0.1237 | 0.1062 | 401 | -3.1312 | 0.0019 |
| Treatment | 0.6173 | 0.4412 | 0.7933 | 0.0896 | 401 | 6.8921 | 0.0000 |
| Panic Disorder | 0.0806 | -0.1876 | 0.3487 | 0.1364 | 401 | 0.5906 | 0.5551 |
| **Rapid response: model with moderation (interaction) effect** | | |  |  |  |  |  |
| Intercept | -0.3467 | -0.5596 | -0.1339 | 0.1083 | 400 | -3.2021 | 0.0015 |
| Treatment | 0.6469 | 0.4594 | 0.8345 | 0.0954 | 400 | 6.7809 | 0.0000 |
| Panic Disorder | 0.2249 | -0.1866 | 0.6364 | 0.2093 | 400 | 1.0744 | 0.2833 |
| Treatment* Panic Disorder | -0.2496 | -0.7903 | 0.2912 | 0.2751 | 400 | -0.9073 | 0.3648 |
| **Post-rapid response: main effects model** | |  |  |  |  |  |  |
| Intercept | -0.3172 | -0.5715 | -0.0630 | 0.1292 | 294 | -2.4553 | 0.0147 |
| Treatment | 0.4484 | 0.2397 | 0.6571 | 0.1061 | 294 | 4.2276 | 0.0000 |
| Panic Disorder | 0.1740 | -0.1534 | 0.5014 | 0.1664 | 294 | 1.0457 | 0.2966 |
| **Post-rapid response: model with moderation (interaction) effect** | | | |  |  |  |  |
| Intercept | -0.3223 | -0.5800 | -0.0646 | 0.1309 | 293 | -2.4617 | 0.0144 |
| Treatment | 0.4583 | 0.2375 | 0.6791 | 0.1122 | 293 | 4.0846 | 0.0001 |
| Panic Disorder | 0.2344 | -0.3097 | 0.7786 | 0.2765 | 293 | 0.8480 | 0.3971 |
| Treatment* Panic Disorder | -0.0949 | -0.7751 | 0.5854 | 0.3456 | 293 | -0.2744 | 0.7839 |
| **Rapid response: main effects model** |  |  |  |  |  |  |  |
| Intercept | -0.3130 | -0.5182 | -0.1079 | 0.1043 | 401 | -3.0002 | 0.0029 |
| Treatment | 0.6246 | 0.4484 | 0.8007 | 0.0896 | 401 | 6.9698 | 0.0000 |
| Agoraphobia | -0.1504 | -0.4691 | 0.1684 | 0.1621 | 401 | -0.9274 | 0.3543 |
| **Rapid response: model with moderation (interaction) effect** | | |  |  |  |  |  |
| Intercept | -0.2997 | -0.5051 | -0.0943 | 0.1045 | 400 | -2.8680 | 0.0043 |
| Treatment | 0.5989 | 0.4151 | 0.7828 | 0.0935 | 400 | 6.4039 | 0.0000 |
| Agoraphobia | -0.3522 | -0.8712 | 0.1668 | 0.2640 | 400 | -1.3342 | 0.1829 |
| Treatment* Agoraphobia | 0.3182 | -0.3287 | 0.9651 | 0.3291 | 400 | 0.9669 | 0.3342 |
| **Post-rapid response: main effects model** | |  |  |  |  |  |  |
| Intercept | -0.3072 | -0.5657 | -0.0488 | 0.1313 | 294 | -2.3398 | 0.0200 |
| Treatment | 0.4533 | 0.2446 | 0.6621 | 0.1061 | 294 | 4.2746 | 0.0000 |
| Agoraphobia | 0.0776 | -0.2642 | 0.4194 | 0.1737 | 294 | 0.4467 | 0.6554 |
| **Post-rapid response: model with moderation (interaction) effect** | | | |  |  |  |  |
| Intercept | -0.3188 | -0.5801 | -0.0575 | 0.1328 | 293 | -2.4008 | 0.0170 |
| Treatment | 0.4746 | 0.2547 | 0.6944 | 0.1117 | 293 | 4.2482 | 0.0000 |
| Agoraphobia | 0.2119 | -0.3400 | 0.7638 | 0.2804 | 293 | 0.7557 | 0.4504 |
| Treatment* Agoraphobia | -0.2176 | -0.9190 | 0.4838 | 0.3564 | 293 | -0.6106 | 0.5420 |
| **Rapid response: main effects model** |  |  |  |  |  |  |  |
| Intercept | -0.2960 | -0.4787 | -0.1132 | 0.0929 | 299 | -3.1875 | 0.0016 |
| Treatment | 0.3848 | 0.1870 | 0.5827 | 0.1005 | 299 | 3.8277 | 0.0002 |
| Substance Use Disorder (lifetime) | 0.0499 | -0.3155 | 0.4152 | 0.1856 | 299 | 0.2686 | 0.7885 |
| **Rapid response: model with moderation (interaction) effect** | | |  |  |  |  |  |
| Intercept | -0.2824 | -0.4692 | -0.0956 | 0.0949 | 298 | -2.9759 | 0.0032 |
| Treatment | 0.3594 | 0.1525 | 0.5664 | 0.1052 | 298 | 3.4180 | 0.0007 |
| Substance Use Disorder (lifetime) | -0.1113 | -0.6402 | 0.4176 | 0.2687 | 298 | -0.4141 | 0.6791 |
| Treatment* Substance Use Disorder (lifetime) | 0.3014 | -0.4158 | 1.0186 | 0.3644 | 298 | 0.8271 | 0.4088 |
| **Post-rapid response: main effects model** | |  |  |  |  |  |  |
| Intercept | -0.2790 | -0.5331 | -0.0250 | 0.1288 | 200 | -2.1658 | 0.0315 |
| Treatment | 0.2196 | -0.0195 | 0.4588 | 0.1213 | 200 | 1.8112 | 0.0716 |
| Substance Use Disorder (lifetime) | 0.0979 | -0.4865 | 0.6823 | 0.2964 | 200 | 0.3303 | 0.7415 |
| **Post-rapid response: model with moderation (interaction) effect** | | | |  |  |  |  |
| Intercept | -0.2686 | -0.5281 | -0.0091 | 0.1316 | 199 | -2.0410 | 0.0426 |
| Treatment | 0.2020 | -0.0434 | 0.4474 | 0.1244 | 199 | 1.6233 | 0.1061 |
| Substance Use Disorder (lifetime) | -0.0925 | -0.9088 | 0.7238 | 0.4139 | 199 | -0.2234 | 0.8234 |
| Treatment* Substance Use Disorder (lifetime) | 0.3767 | -0.7640 | 1.5173 | 0.5784 | 199 | 0.6512 | 0.5157 |
| **Rapid response: main effects model** |  |  |  |  |  |  |  |
| Intercept | -0.1150 | -0.3554 | 0.1255 | 0.1221 | 277 | -0.9412 | 0.3474 |
| Treatment | 0.2608 | 0.0428 | 0.4788 | 0.1108 | 277 | 2.3549 | 0.0192 |
| Alcohol Use Disorder (lifetime) | 0.0064 | -0.2761 | 0.2890 | 0.1435 | 277 | 0.0449 | 0.9642 |
| **Rapid response: model with moderation (interaction) effect** | | |  |  |  |  |  |
| Intercept | -0.1015 | -0.3508 | 0.1477 | 0.1266 | 276 | -0.8021 | 0.4232 |
| Treatment | 0.2353 | -0.0135 | 0.4840 | 0.1264 | 276 | 1.8618 | 0.0637 |
| Alcohol Use Disorder (lifetime) | -0.0532 | -0.4488 | 0.3425 | 0.2010 | 276 | -0.2646 | 0.7915 |
| Treatment* Alcohol Use Disorder (lifetime) | 0.1121 | -0.4113 | 0.6356 | 0.2659 | 276 | 0.4217 | 0.6736 |
| **Post-rapid response: main effects model** | |  |  |  |  |  |  |
| Intercept | -0.0235 | -0.4313 | 0.3844 | 0.2067 | 179 | -0.1136 | 0.9097 |
| Treatment | 0.2302 | -0.0390 | 0.4994 | 0.1364 | 179 | 1.6877 | 0.0932 |
| Alcohol Use Disorder (lifetime) | 0.0271 | -0.3461 | 0.4003 | 0.1891 | 179 | 0.1432 | 0.8863 |
| **Post-rapid response: model with moderation (interaction) effect** | | | |  |  |  |  |
| Intercept | 0.0171 | -0.3971 | 0.4314 | 0.2099 | 178 | 0.0815 | 0.9351 |
| Treatment | 0.1496 | -0.1608 | 0.4600 | 0.1573 | 178 | 0.9511 | 0.3429 |
| Alcohol Use Disorder (lifetime) | -0.1324 | -0.6150 | 0.3502 | 0.2446 | 178 | -0.5414 | 0.5889 |
| Treatment* Alcohol Use Disorder (lifetime) | 0.3269 | -0.3000 | 0.9538 | 0.3177 | 178 | 1.0290 | 0.3049 |
| **Rapid response: main effects model** |  |  |  |  |  |  |  |
| Intercept | -0.2961 | -0.4789 | -0.1133 | 0.0930 | 513 | -3.1820 | 0.0016 |
| Treatment | 0.5704 | 0.4111 | 0.7297 | 0.0811 | 513 | 7.0354 | 0.0000 |
| NumYearsEducation | 0.0003 | -0.0842 | 0.0848 | 0.0430 | 513 | 0.0069 | 0.9945 |
| **Rapid response: model with moderation (interaction) effect** | | |  |  |  |  |  |
| Intercept | -0.2963 | -0.4792 | -0.1135 | 0.0931 | 512 | -3.1844 | 0.0015 |
| Treatment | 0.5705 | 0.4111 | 0.7299 | 0.0812 | 512 | 7.0298 | 0.0000 |
| NumYearsEducation | -0.0063 | -0.1321 | 0.1196 | 0.0640 | 512 | -0.0976 | 0.9223 |
| Treatment* NumYearsEducation | 0.0113 | -0.1493 | 0.1719 | 0.0817 | 512 | 0.1383 | 0.8901 |
| **Post-rapid response: main effects model** | |  |  |  |  |  |  |
| Intercept | -0.2512 | -0.4913 | -0.0110 | 0.1221 | 399 | -2.0563 | 0.0404 |
| Treatment | 0.5231 | 0.3503 | 0.6959 | 0.0879 | 399 | 5.9526 | 0.0000 |
| NumYearsEducation | -0.0167 | -0.1097 | 0.0763 | 0.0473 | 399 | -0.3535 | 0.7239 |
| **Post-rapid response: model with moderation (interaction) effect** | | | |  |  |  |  |
| Intercept | -0.2532 | -0.4940 | -0.0124 | 0.1225 | 398 | -2.0668 | 0.0394 |
| Treatment | 0.5258 | 0.3518 | 0.6998 | 0.0885 | 398 | 5.9402 | 0.0000 |
| NumYearsEducation | -0.0311 | -0.1690 | 0.1069 | 0.0702 | 398 | -0.4427 | 0.6582 |
| Treatment* NumYearsEducation | 0.0249 | -0.1517 | 0.2014 | 0.0898 | 398 | 0.2769 | 0.7820 |
| **Rapid response: main effects model** |  |  |  |  |  |  |  |
| Intercept | -0.0747 | -0.3116 | 0.1621 | 0.1204 | 351 | -0.6207 | 0.5352 |
| Treatment | 0.3789 | 0.1795 | 0.5784 | 0.1014 | 351 | 3.7365 | 0.0002 |
| Married | 0.0950 | -0.1196 | 0.3097 | 0.1091 | 351 | 0.8707 | 0.3845 |
| **Rapid response: model with moderation (interaction) effect** | | |  |  |  |  |  |
| Intercept | -0.1099 | -0.3586 | 0.1388 | 0.1265 | 350 | -0.8691 | 0.3854 |
| Treatment | 0.4503 | 0.2047 | 0.6960 | 0.1249 | 350 | 3.6061 | 0.0004 |
| Married | 0.2090 | -0.1046 | 0.5225 | 0.1594 | 350 | 1.3109 | 0.1908 |
| Treatment* Married | -0.2091 | -0.6283 | 0.2101 | 0.2131 | 350 | -0.9811 | 0.3272 |
| **Post-rapid response: main effects model** | |  |  |  |  |  |  |
| Intercept | -0.2097 | -0.5444 | 0.1249 | 0.1699 | 246 | -1.2345 | 0.2182 |
| Treatment | 0.5029 | 0.2624 | 0.7433 | 0.1221 | 246 | 4.1191 | 0.0001 |
| Married | 0.1544 | -0.0948 | 0.4036 | 0.1265 | 246 | 1.2207 | 0.2234 |
| **Post-rapid response: model with moderation (interaction) effect** | | | |  |  |  |  |
| Intercept | -0.2986 | -0.6509 | 0.0538 | 0.1789 | 245 | -1.6691 | 0.0964 |
| Treatment | 0.6819 | 0.3759 | 0.9879 | 0.1553 | 245 | 4.3898 | 0.0000 |
| Married | 0.4094 | 0.0408 | 0.7779 | 0.1871 | 245 | 2.1879 | 0.0296 |
| Treatment* Married | -0.4566 | -0.9436 | 0.0304 | 0.2473 | 245 | -1.8466 | 0.0660 |
| **Rapid response: main effects model** |  |  |  |  |  |  |  |
| Intercept | -0.1990 | -0.4212 | 0.0233 | 0.1132 | 616 | -1.7582 | 0.0792 |
| Treatment | 0.5412 | 0.3904 | 0.6919 | 0.0768 | 616 | 7.0484 | 0.0000 |
| White | -0.0970 | -0.2980 | 0.1040 | 0.1024 | 616 | -0.9476 | 0.3437 |
| **Rapid response: model with moderation (interaction) effect** | | |  |  |  |  |  |
| Intercept | -0.1792 | -0.4511 | 0.0926 | 0.1384 | 615 | -1.2946 | 0.1959 |
| Treatment | 0.5101 | 0.2189 | 0.8012 | 0.1483 | 615 | 3.4402 | 0.0006 |
| White | -0.1233 | -0.4127 | 0.1662 | 0.1474 | 615 | -0.8363 | 0.4033 |
| Treatment* White | 0.0426 | -0.2977 | 0.3829 | 0.1733 | 615 | 0.2458 | 0.8059 |
| **Post-rapid response: main effects model** | |  |  |  |  |  |  |
| Intercept | -0.1316 | -0.3993 | 0.1361 | 0.1362 | 499 | -0.9658 | 0.3346 |
| Treatment | 0.3608 | 0.1912 | 0.5304 | 0.0863 | 499 | 4.1789 | 0.0000 |
| White | -0.0232 | -0.2491 | 0.2027 | 0.1150 | 499 | -0.2019 | 0.8401 |
| **Post-rapid response: model with moderation (interaction) effect** | | | |  |  |  |  |
| Intercept | -0.2410 | -0.5557 | 0.0737 | 0.1602 | 498 | -1.5046 | 0.1331 |
| Treatment | 0.5318 | 0.2249 | 0.8386 | 0.1562 | 498 | 3.4052 | 0.0007 |
| White | 0.1283 | -0.1923 | 0.4489 | 0.1632 | 498 | 0.7861 | 0.4322 |
| Treatment* White | -0.2458 | -0.6131 | 0.1215 | 0.1869 | 498 | -1.3146 | 0.1892 |
| **Rapid response: main effects model** |  |  |  |  |  |  |  |
| Intercept | -0.2929 | -0.4521 | -0.1337 | 0.0811 | 616 | -3.6139 | 0.0003 |
| Treatment | 0.5389 | 0.3885 | 0.6893 | 0.0766 | 616 | 7.0383 | 0.0000 |
| Black | 0.2454 | -0.0293 | 0.5202 | 0.1399 | 616 | 1.7544 | 0.0799 |
| **Rapid response: model with moderation (interaction) effect** | | |  |  |  |  |  |
| Intercept | -0.2953 | -0.4564 | -0.1342 | 0.0820 | 615 | -3.5993 | 0.0003 |
| Treatment | 0.5431 | 0.3854 | 0.7007 | 0.0803 | 615 | 6.7647 | 0.0000 |
| Black | 0.2759 | -0.1639 | 0.7157 | 0.2239 | 615 | 1.2319 | 0.2185 |
| Treatment* Black | -0.0463 | -0.5748 | 0.4822 | 0.2691 | 615 | -0.1722 | 0.8633 |
| **Post-rapid response: main effects model** | |  |  |  |  |  |  |
| Intercept | -0.1642 | -0.3778 | 0.0494 | 0.1087 | 499 | -1.5101 | 0.1316 |
| Treatment | 0.3536 | 0.1846 | 0.5226 | 0.0860 | 499 | 4.1104 | 0.0000 |
| Black | 0.1784 | -0.1152 | 0.4720 | 0.1494 | 499 | 1.1936 | 0.2332 |
| **Post-rapid response: model with moderation (interaction) effect** | | | |  |  |  |  |
| Intercept | -0.1349 | -0.3511 | 0.0813 | 0.1100 | 498 | -1.2260 | 0.2208 |
| Treatment | 0.3009 | 0.1229 | 0.4788 | 0.0906 | 498 | 3.3220 | 0.0010 |
| Black | -0.1638 | -0.6359 | 0.3082 | 0.2402 | 498 | -0.6820 | 0.4956 |
| Treatment* Black | 0.5167 | -0.0414 | 1.0747 | 0.2840 | 498 | 1.8191 | 0.0695 |
| **Rapid response: main effects model** |  |  |  |  |  |  |  |
| Intercept | -0.2480 | -0.4181 | -0.0780 | 0.0866 | 616 | -2.8647 | 0.0043 |
| Treatment | 0.5484 | 0.3985 | 0.6984 | 0.0764 | 616 | 7.1821 | 0.0000 |
| Asian | -0.2521 | -0.6115 | 0.1072 | 0.1830 | 616 | -1.3780 | 0.1687 |
| **Rapid response: model with moderation (interaction) effect** | | |  |  |  |  |  |
| Intercept | -0.2432 | -0.4163 | -0.0702 | 0.0881 | 615 | -2.7610 | 0.0059 |
| Treatment | 0.5402 | 0.3814 | 0.6989 | 0.0808 | 615 | 6.6812 | 0.0000 |
| Asian | -0.2964 | -0.7508 | 0.1580 | 0.2314 | 615 | -1.2810 | 0.2007 |
| Treatment* Asian | 0.0774 | -0.4104 | 0.5651 | 0.2484 | 615 | 0.3115 | 0.7555 |
| **Post-rapid response: main effects model** | |  |  |  |  |  |  |
| Intercept | -0.0980 | -0.3080 | 0.1119 | 0.1069 | 499 | -0.9173 | 0.3594 |
| Treatment | 0.3612 | 0.1933 | 0.5292 | 0.0855 | 499 | 4.2258 | 0.0000 |
| Asian | -0.5135 | -0.9682 | -0.0588 | 0.2314 | 499 | -2.2189 | 0.0269 |
| **Post-rapid response: model with moderation (interaction) effect** | | | |  |  |  |  |
| Intercept | -0.0895 | -0.3024 | 0.1235 | 0.1084 | 498 | -0.8251 | 0.4097 |
| Treatment | 0.3457 | 0.1668 | 0.5246 | 0.0911 | 498 | 3.7962 | 0.0002 |
| Asian | -0.5838 | -1.1169 | -0.0507 | 0.2713 | 498 | -2.1517 | 0.0319 |
| Treatment* Asian | 0.1312 | -0.3874 | 0.6497 | 0.2639 | 498 | 0.4970 | 0.6194 |
| **Rapid response: main effects model** |  |  |  |  |  |  |  |
| Intercept | -0.2791 | -0.4429 | -0.1153 | 0.0834 | 616 | -3.3467 | 0.0009 |
| Treatment | 0.5454 | 0.3951 | 0.6957 | 0.0765 | 616 | 7.1247 | 0.0000 |
| OtherRace | 0.1012 | -0.1898 | 0.3922 | 0.1482 | 616 | 0.6831 | 0.4948 |
| **Rapid response: model with moderation (interaction) effect** | | |  |  |  |  |  |
| Intercept | -0.2893 | -0.4541 | -0.1244 | 0.0839 | 615 | -3.4459 | 0.0006 |
| Treatment | 0.5635 | 0.4076 | 0.7195 | 0.0794 | 615 | 7.0948 | 0.0000 |
| OtherRace | 0.2582 | -0.2045 | 0.7208 | 0.2356 | 615 | 1.0959 | 0.2735 |
| Treatment* OtherRace | -0.2522 | -0.8317 | 0.3273 | 0.2951 | 615 | -0.8546 | 0.3931 |
| **Post-rapid response: main effects model** | |  |  |  |  |  |  |
| Intercept | -0.1546 | -0.3628 | 0.0536 | 0.1060 | 499 | -1.4590 | 0.1452 |
| Treatment | 0.3602 | 0.1912 | 0.5292 | 0.0860 | 499 | 4.1875 | 0.0000 |
| OtherRace | 0.0791 | -0.2259 | 0.3841 | 0.1552 | 499 | 0.5094 | 0.6107 |
| **Post-rapid response: model with moderation (interaction) effect** | | | |  |  |  |  |
| Intercept | -0.1656 | -0.3751 | 0.0439 | 0.1066 | 498 | -1.5530 | 0.1211 |
| Treatment | 0.3791 | 0.2030 | 0.5553 | 0.0897 | 498 | 4.2290 | 0.0000 |
| OtherRace | 0.2287 | -0.2676 | 0.7251 | 0.2526 | 498 | 0.9054 | 0.3657 |
| Treatment* OtherRace | -0.2348 | -0.8488 | 0.3791 | 0.3125 | 498 | -0.7515 | 0.4527 |
| **Rapid response: main effects model** |  |  |  |  |  |  |  |
| Intercept | -0.2071 | -0.4165 | 0.0022 | 0.1066 | 495 | -1.9437 | 0.0525 |
| Treatment | 0.5623 | 0.4009 | 0.7238 | 0.0822 | 495 | 6.8444 | 0.0000 |
| Hispanic | 0.2791 | -0.0767 | 0.6349 | 0.1811 | 495 | 1.5412 | 0.1239 |
| **Rapid response: model with moderation (interaction) effect** | | |  |  |  |  |  |
| Intercept | -0.2028 | -0.4117 | 0.0062 | 0.1063 | 494 | -1.9069 | 0.0571 |
| Treatment | 0.5539 | 0.3880 | 0.7198 | 0.0844 | 494 | 6.5593 | 0.0000 |
| Hispanic | 0.1741 | -0.4140 | 0.7622 | 0.2993 | 494 | 0.5817 | 0.5611 |
| Treatment* Hispanic | 0.1639 | -0.5618 | 0.8897 | 0.3694 | 494 | 0.4437 | 0.6574 |
| **Post-rapid response: main effects model** | |  |  |  |  |  |  |
| Intercept | -0.2260 | -0.4807 | 0.0287 | 0.1295 | 379 | -1.7444 | 0.0819 |
| Treatment | 0.5034 | 0.3136 | 0.6932 | 0.0965 | 379 | 5.2146 | 0.0000 |
| Hispanic | 0.4235 | 0.0442 | 0.8028 | 0.1929 | 379 | 2.1956 | 0.0287 |
| **Post-rapid response: model with moderation (interaction) effect** | | | |  |  |  |  |
| Intercept | -0.2225 | -0.4787 | 0.0336 | 0.1303 | 378 | -1.7081 | 0.0884 |
| Treatment | 0.4970 | 0.3012 | 0.6928 | 0.0996 | 378 | 4.9912 | 0.0000 |
| Hispanic | 0.3481 | -0.3255 | 1.0217 | 0.3426 | 378 | 1.0161 | 0.3102 |
| Treatment* Hispanic | 0.1090 | -0.6957 | 0.9138 | 0.4093 | 378 | 0.2664 | 0.7901 |
| **Rapid response: main effects model** |  |  |  |  |  |  |  |
| Intercept | -0.3505 | -0.5443 | -0.1567 | 0.0986 | 485 | -3.5530 | 0.0004 |
| Treatment | 0.6865 | 0.5249 | 0.8481 | 0.0822 | 485 | 8.3473 | 0.0000 |
| MADRS Suicidal Ideation item (pre) | -0.1422 | -0.2364 | -0.0481 | 0.0479 | 485 | -2.9688 | 0.0031 |
| **Rapid response: model with moderation (interaction) effect** | | |  |  |  |  |  |
| Intercept | -0.3504 | -0.5444 | -0.1565 | 0.0987 | 484 | -3.5501 | 0.0004 |
| Treatment | 0.6865 | 0.5247 | 0.8483 | 0.0823 | 484 | 8.3382 | 0.0000 |
| MADRS Suicidal Ideation item (pre) | -0.1413 | -0.2745 | -0.0081 | 0.0678 | 484 | -2.0840 | 0.0377 |
| Treatment* MADRS Suicidal Ideation item (pre) | -0.0016 | -0.1633 | 0.1601 | 0.0823 | 484 | -0.0194 | 0.9845 |
| **Post-rapid response: main effects model** | |  |  |  |  |  |  |
| Intercept | -0.3168 | -0.5448 | -0.0888 | 0.1160 | 468 | -2.7307 | 0.0066 |
| Treatment | 0.5090 | 0.3458 | 0.6722 | 0.0830 | 468 | 6.1295 | 0.0000 |
| MADRS Suicidal Ideation item (pre) | -0.0765 | -0.1726 | 0.0197 | 0.0489 | 468 | -1.5620 | 0.1190 |
| **Post-rapid response: model with moderation (interaction) effect** | | | |  |  |  |  |
| Intercept | -0.3161 | -0.5443 | -0.0880 | 0.1161 | 467 | -2.7230 | 0.0067 |
| Treatment | 0.5093 | 0.3459 | 0.6726 | 0.0831 | 467 | 6.1268 | 0.0000 |
| MADRS Suicidal Ideation item (pre) | -0.0567 | -0.1916 | 0.0782 | 0.0687 | 467 | -0.8260 | 0.4092 |
| Treatment* MADRS Suicidal Ideation item (pre) | -0.0338 | -0.1958 | 0.1281 | 0.0824 | 467 | -0.4104 | 0.6817 |
| **Rapid response: main effects model** |  |  |  |  |  |  |  |
| Intercept | -0.3199 | -0.5169 | -0.1229 | 0.1002 | 421 | -3.1920 | 0.0015 |
| Treatment | 0.5053 | 0.3342 | 0.6765 | 0.0871 | 421 | 5.8029 | 0.0000 |
| DurationofCurrentMDEmonths(natLog) | -0.0408 | -0.1370 | 0.0554 | 0.0489 | 421 | -0.8341 | 0.4047 |
| **Rapid response: model with moderation (interaction) effect** | | |  |  |  |  |  |
| Intercept | -0.3212 | -0.5185 | -0.1239 | 0.1004 | 420 | -3.1999 | 0.0015 |
| Treatment | 0.5059 | 0.3345 | 0.6773 | 0.0872 | 420 | 5.8027 | 0.0000 |
| DurationofCurrentMDEmonths(natLog) | -0.0562 | -0.1844 | 0.0721 | 0.0653 | 420 | -0.8607 | 0.3899 |
| Treatment* DurationofCurrentMDEmonths(natLog) | 0.0312 | -0.1413 | 0.2038 | 0.0878 | 420 | 0.3558 | 0.7221 |
| **Post-rapid response: main effects model** | |  |  |  |  |  |  |
| Intercept | -0.1804 | -0.5028 | 0.1420 | 0.1639 | 322 | -1.1009 | 0.2718 |
| Treatment | 0.4347 | 0.2461 | 0.6232 | 0.0959 | 322 | 4.5347 | 0.0000 |
| DurationofCurrentMDEmonths(natLog) | -0.0285 | -0.1461 | 0.0892 | 0.0598 | 322 | -0.4762 | 0.6343 |
| **Post-rapid response: model with moderation (interaction) effect** | | | |  |  |  |  |
| Intercept | -0.1788 | -0.5011 | 0.1436 | 0.1639 | 321 | -1.0911 | 0.2761 |
| Treatment | 0.4361 | 0.2472 | 0.6250 | 0.0960 | 321 | 4.5411 | 0.0000 |
| DurationofCurrentMDEmonths(natLog) | -0.0036 | -0.1662 | 0.1590 | 0.0827 | 321 | -0.0437 | 0.9651 |
| Treatment* DurationofCurrentMDEmonths(natLog) | -0.0439 | -0.2417 | 0.1539 | 0.1006 | 321 | -0.4365 | 0.6628 |
| **Rapid response: main effects model** |  |  |  |  |  |  |  |
| Intercept | -0.2533 | -0.5359 | 0.0293 | 0.1437 | 377 | -1.7627 | 0.0788 |
| Treatment | 0.4483 | 0.2639 | 0.6326 | 0.0938 | 377 | 4.7808 | 0.0000 |
| RecurrentMDD | -0.0045 | -0.2340 | 0.2250 | 0.1167 | 377 | -0.0386 | 0.9692 |
| **Rapid response: model with moderation (interaction) effect** | | |  |  |  |  |  |
| Intercept | -0.1459 | -0.4849 | 0.1930 | 0.1724 | 376 | -0.8465 | 0.3978 |
| Treatment | 0.2619 | -0.1140 | 0.6379 | 0.1912 | 376 | 1.3700 | 0.1715 |
| RecurrentMDD | -0.1434 | -0.4784 | 0.1916 | 0.1704 | 376 | -0.8416 | 0.4006 |
| Treatment* RecurrentMDD | 0.2452 | -0.1857 | 0.6760 | 0.2191 | 376 | 1.1189 | 0.2639 |
| **Post-rapid response: main effects model** | |  |  |  |  |  |  |
| Intercept | -0.3109 | -0.7063 | 0.0845 | 0.2009 | 279 | -1.5477 | 0.1228 |
| Treatment | 0.3990 | 0.1909 | 0.6071 | 0.1057 | 279 | 3.7748 | 0.0002 |
| RecurrentMDD | 0.1473 | -0.1270 | 0.4216 | 0.1394 | 279 | 1.0569 | 0.2914 |
| **Post-rapid response: model with moderation (interaction) effect** | | | |  |  |  |  |
| Intercept | -0.1578 | -0.6211 | 0.3055 | 0.2354 | 278 | -0.6706 | 0.5030 |
| Treatment | 0.1570 | -0.2759 | 0.5899 | 0.2199 | 278 | 0.7141 | 0.4757 |
| RecurrentMDD | -0.0471 | -0.4570 | 0.3628 | 0.2082 | 278 | -0.2262 | 0.8212 |
| Treatment* RecurrentMDD | 0.3146 | -0.1792 | 0.8085 | 0.2509 | 278 | 1.2541 | 0.2109 |
| **Rapid response: main effects model** |  |  |  |  |  |  |  |
| Intercept | -0.2191 | -0.4519 | 0.0138 | 0.1184 | 359 | -1.8502 | 0.0651 |
| Treatment | 0.4157 | 0.2257 | 0.6057 | 0.0966 | 359 | 4.3022 | 0.0000 |
| NumDepEpisodes(natLog) | 0.0301 | -0.0765 | 0.1366 | 0.0542 | 359 | 0.5551 | 0.5792 |
| **Rapid response: model with moderation (interaction) effect** | | |  |  |  |  |  |
| Intercept | -0.2103 | -0.4419 | 0.0213 | 0.1178 | 358 | -1.7857 | 0.0750 |
| Treatment | 0.4140 | 0.2244 | 0.6037 | 0.0964 | 358 | 4.2931 | 0.0000 |
| NumDepEpisodes(natLog) | -0.0520 | -0.1983 | 0.0943 | 0.0744 | 358 | -0.6993 | 0.4848 |
| Treatment* NumDepEpisodes(natLog) | 0.1548 | -0.0351 | 0.3447 | 0.0966 | 358 | 1.6029 | 0.1098 |
| **Post-rapid response: main effects model** | |  |  |  |  |  |  |
| Intercept | -0.2545 | -0.5771 | 0.0681 | 0.1638 | 261 | -1.5535 | 0.1215 |
| Treatment | 0.4165 | 0.1973 | 0.6357 | 0.1113 | 261 | 3.7412 | 0.0002 |
| NumDepEpisodes(natLog) | 0.0726 | -0.0696 | 0.2147 | 0.0722 | 261 | 1.0051 | 0.3158 |
| **Post-rapid response: model with moderation (interaction) effect** | | | |  |  |  |  |
| Intercept | -0.2431 | -0.5623 | 0.0762 | 0.1621 | 260 | -1.4991 | 0.1350 |
| Treatment | 0.4299 | 0.2109 | 0.6488 | 0.1112 | 260 | 3.8662 | 0.0001 |
| NumDepEpisodes(natLog) | -0.0273 | -0.2078 | 0.1533 | 0.0917 | 260 | -0.2973 | 0.7664 |
| Treatment* NumDepEpisodes(natLog) | 0.2064 | -0.0254 | 0.4382 | 0.1177 | 260 | 1.7535 | 0.0807 |
| **Rapid response: main effects model** | |  |  |  |  |  |  |
| Intercept | -0.0171 | -0.2533 | 0.2192 | 0.1201 | 336 | -0.1423 | 0.8869 |
| Treatment | 0.3654 | 0.1634 | 0.5674 | 0.1027 | 336 | 3.5579 | 0.0004 |
| AgeofIllnessOnset | 0.0365 | -0.0736 | 0.1466 | 0.0560 | 336 | 0.6516 | 0.5151 |
| **Rapid response: model with moderation (interaction) effect** | | | |  |  |  |  |
| Intercept | -0.0197 | -0.2556 | 0.2163 | 0.1199 | 335 | -0.1641 | 0.8697 |
| Treatment | 0.3660 | 0.1640 | 0.5680 | 0.1027 | 335 | 3.5636 | 0.0004 |
| Ageofillnessonset | 0.0855 | -0.0596 | 0.2306 | 0.0738 | 335 | 1.1591 | 0.2472 |
| Treatment* Ageofillnessonset | -0.1035 | -0.3031 | 0.0960 | 0.1015 | 335 | -1.0205 | 0.3082 |
| **Post-rapid response: main effects model** | |  |  |  |  |  |  |
| Intercept | -0.1293 | -0.4633 | 0.2047 | 0.1695 | 237 | -0.7625 | 0.4465 |
| Treatment | 0.5037 | 0.2651 | 0.7422 | 0.1211 | 237 | 4.1595 | 0.0000 |
| AgeofIllnessOnset | -0.0194 | -0.1490 | 0.1103 | 0.0658 | 237 | -0.2941 | 0.7689 |
| **Post-rapid response: model with moderation (interaction) effect** | | | |  |  |  |  |
| Intercept | -0.1180 | -0.4518 | 0.2159 | 0.1695 | 236 | -0.6962 | 0.4870 |
| Treatment | 0.4870 | 0.2441 | 0.7299 | 0.1233 | 236 | 3.9504 | 0.0001 |
| Ageofillnessonset | -0.0627 | -0.2364 | 0.1110 | 0.0882 | 236 | -0.7113 | 0.4776 |
| Treatment* Ageofillnessonset | 0.0877 | -0.1466 | 0.3220 | 0.1189 | 236 | 0.7377 | 0.4614 |
| **Rapid response: main effects model** |  |  |  |  |  |  |  |
| Intercept | -0.2488 | -0.4884 | -0.0092 | 0.1217 | 293 | -2.0439 | 0.0419 |
| Treatment | 0.6280 | 0.4220 | 0.8340 | 0.1047 | 293 | 5.9992 | 0.0000 |
| Number of failed adequate trials | -0.0917 | -0.2162 | 0.0328 | 0.0633 | 293 | -1.4492 | 0.1484 |
| **Rapid response: model with moderation (interaction) effect** | | |  |  |  |  |  |
| Intercept | -0.2539 | -0.4894 | -0.0185 | 0.1196 | 292 | -2.1225 | 0.0346 |
| Treatment | 0.6316 | 0.4257 | 0.8375 | 0.1046 | 292 | 6.0367 | 0.0000 |
| Number of failed adequate trials | -0.1677 | -0.3386 | 0.0033 | 0.0869 | 292 | -1.9304 | 0.0545 |
| Treatment* Number of failed adequate trials | 0.1317 | -0.0736 | 0.3370 | 0.1043 | 292 | 1.2625 | 0.2078 |
| **Post-rapid response: main effects model** | |  |  |  |  |  |  |
| Intercept | -0.2829 | -0.6428 | 0.0770 | 0.1829 | 288 | -1.5471 | 0.1229 |
| Treatment | 0.5477 | 0.3384 | 0.7571 | 0.1064 | 288 | 5.1496 | 0.0000 |
| Number of failed adequate trials | -0.1172 | -0.2511 | 0.0168 | 0.0680 | 288 | -1.7219 | 0.0862 |
| **Post-rapid response: model with moderation (interaction) effect** | | | |  |  |  |  |
| Intercept | -0.2742 | -0.6397 | 0.0913 | 0.1857 | 287 | -1.4766 | 0.1409 |
| Treatment | 0.5403 | 0.3313 | 0.7493 | 0.1062 | 287 | 5.0882 | 0.0000 |
| Number of failed adequate trials | -0.0203 | -0.2014 | 0.1608 | 0.0920 | 287 | -0.2207 | 0.8255 |
| Treatment* Number of failed adequate trials | -0.1651 | -0.3743 | 0.0441 | 0.1063 | 287 | -1.5530 | 0.1215 |
| **Rapid response: main effects model** |  |  |  |  |  |  |  |
| Intercept | -0.3129 | -0.5289 | -0.0970 | 0.1099 | 523 | -2.8463 | 0.0046 |
| Treatment | 0.5929 | 0.4324 | 0.7533 | 0.0817 | 523 | 7.2577 | 0.0000 |
| Presence of concurrent psych meds | 0.0838 | -0.1629 | 0.3304 | 0.1256 | 523 | 0.6673 | 0.5049 |
| **Rapid response: model with moderation (interaction) effect** | | |  |  |  |  |  |
| Intercept | -0.3213 | -0.5542 | -0.0884 | 0.1185 | 522 | -2.7102 | 0.0069 |
| Treatment | 0.6077 | 0.3851 | 0.8302 | 0.1133 | 522 | 5.3643 | 0.0000 |
| Presence of concurrent psych meds | 0.1003 | -0.2004 | 0.4010 | 0.1530 | 522 | 0.6554 | 0.5125 |
| Treatment* Presence of concurrent psych meds | -0.0310 | -0.3532 | 0.2912 | 0.1640 | 522 | -0.1891 | 0.8501 |
| **Post-rapid response: main effects model** | |  |  |  |  |  |  |
| Intercept | -0.2677 | -0.5325 | -0.0029 | 0.1348 | 498 | -1.9865 | 0.0475 |
| Treatment | 0.3746 | 0.2063 | 0.5429 | 0.0857 | 498 | 4.3725 | 0.0000 |
| Presence of concurrent psych meds | 0.1606 | -0.1201 | 0.4414 | 0.1429 | 498 | 1.1242 | 0.2615 |
| **Post-rapid response: model with moderation (interaction) effect** | | | |  |  |  |  |
| Intercept | -0.2379 | -0.5177 | 0.0419 | 0.1424 | 497 | -1.6705 | 0.0954 |
| Treatment | 0.3208 | 0.0873 | 0.5542 | 0.1188 | 497 | 2.6999 | 0.0072 |
| Presence of concurrent psych meds | 0.1026 | -0.2281 | 0.4334 | 0.1684 | 497 | 0.6096 | 0.5424 |
| Treatment* Presence of concurrent psych meds | 0.1125 | -0.2258 | 0.4508 | 0.1722 | 497 | 0.6535 | 0.5137 |
| **Rapid response: main effects model** |  |  |  |  |  |  |  |
| Intercept | -0.3345 | -0.5182 | -0.1508 | 0.0935 | 448 | -3.5792 | 0.0004 |
| Treatment | 0.6411 | 0.4701 | 0.8121 | 0.0870 | 448 | 7.3671 | 0.0000 |
| Concurrent benzodiazepine | -0.0705 | -0.4033 | 0.2623 | 0.1693 | 448 | -0.4164 | 0.6774 |
| **Rapid response: model with moderation (interaction) effect** | | |  |  |  |  |  |
| Intercept | -0.3443 | -0.5310 | -0.1576 | 0.0950 | 447 | -3.6243 | 0.0003 |
| Treatment | 0.6581 | 0.4794 | 0.8368 | 0.0909 | 447 | 7.2378 | 0.0000 |
| Concurrent benzodiazepine | 0.0394 | -0.4305 | 0.5094 | 0.2391 | 447 | 0.1650 | 0.8691 |
| Treatment* Concurrent benzodiazepine | -0.2026 | -0.8125 | 0.4073 | 0.3103 | 447 | -0.6529 | 0.5142 |
| **Post-rapid response: main effects model** | |  |  |  |  |  |  |
| Intercept | -0.2475 | -0.4657 | -0.0293 | 0.1110 | 423 | -2.2298 | 0.0263 |
| Treatment | 0.3205 | 0.1387 | 0.5022 | 0.0925 | 423 | 3.4661 | 0.0006 |
| Concurrent benzodiazepine | 0.1162 | -0.2338 | 0.4663 | 0.1781 | 423 | 0.6527 | 0.5143 |
| **Post-rapid response: model with moderation (interaction) effect** | | | |  |  |  |  |
| Intercept | -0.2462 | -0.4668 | -0.0256 | 0.1122 | 422 | -2.1935 | 0.0288 |
| Treatment | 0.3182 | 0.1279 | 0.5086 | 0.0968 | 422 | 3.2870 | 0.0011 |
| Concurrent benzodiazepine | 0.1023 | -0.3880 | 0.5927 | 0.2495 | 422 | 0.4102 | 0.6818 |
| Treatment* Concurrent benzodiazepine | 0.0256 | -0.6059 | 0.6572 | 0.3213 | 422 | 0.0798 | 0.9365 |
| **Rapid response: main effects model** |  |  |  |  |  |  |  |
| Intercept | -0.2889 | -0.5094 | -0.0684 | 0.1121 | 349 | -2.5773 | 0.0104 |
| Treatment | 0.5998 | 0.3977 | 0.8019 | 0.1028 | 349 | 5.8366 | 0.0000 |
| Number of concurrent psych meds | 0.0810 | -0.0699 | 0.2319 | 0.0767 | 349 | 1.0561 | 0.2917 |
| **Rapid response: model with moderation (interaction) effect** | | |  |  |  |  |  |
| Intercept | -0.2922 | -0.5132 | -0.0712 | 0.1124 | 348 | -2.6001 | 0.0097 |
| Treatment | 0.5998 | 0.3976 | 0.8020 | 0.1028 | 348 | 5.8335 | 0.0000 |
| Number of concurrent psych meds | 0.1184 | -0.0626 | 0.2994 | 0.0920 | 348 | 1.2862 | 0.1992 |
| Treatment* Number of concurrent psych meds | -0.0766 | -0.2813 | 0.1281 | 0.1041 | 348 | -0.7359 | 0.4623 |
| **Post-rapid response: main effects model** | |  |  |  |  |  |  |
| Intercept | -0.1855 | -0.4491 | 0.0781 | 0.1340 | 330 | -1.3842 | 0.1672 |
| Treatment | 0.3613 | 0.1452 | 0.5774 | 0.1099 | 330 | 3.2887 | 0.0011 |
| Number of concurrent psych meds | 0.1063 | -0.0583 | 0.2710 | 0.0837 | 330 | 1.2701 | 0.2050 |
| **Post-rapid response: model with moderation (interaction) effect** | | | |  |  |  |  |
| Intercept | -0.1848 | -0.4487 | 0.0792 | 0.1342 | 329 | -1.3772 | 0.1694 |
| Treatment | 0.3613 | 0.1449 | 0.5778 | 0.1100 | 329 | 3.2841 | 0.0011 |
| Number of concurrent psych meds | 0.0998 | -0.0948 | 0.2944 | 0.0989 | 329 | 1.0086 | 0.3139 |
| Treatment* Number of concurrent psych meds | 0.0138 | -0.2024 | 0.2301 | 0.1099 | 329 | 0.1257 | 0.9001 |
| **Rapid response: main effects model** |  |  |  |  |  |  |  |
| Intercept | -0.2384 | -0.4543 | -0.0224 | 0.1098 | 366 | -2.1706 | 0.0306 |
| Treatment | 0.5354 | 0.3473 | 0.7235 | 0.0956 | 366 | 5.5980 | 0.0000 |
| Smoker | -0.0061 | -0.2373 | 0.2251 | 0.1176 | 366 | -0.0518 | 0.9587 |
| **Rapid response: model with moderation (interaction) effect** | | |  |  |  |  |  |
| Intercept | -0.2661 | -0.4911 | -0.0412 | 0.1144 | 365 | -2.3264 | 0.0205 |
| Treatment | 0.5871 | 0.3642 | 0.8099 | 0.1133 | 365 | 5.1802 | 0.0000 |
| Smoker | 0.0864 | -0.2284 | 0.4013 | 0.1601 | 365 | 0.5400 | 0.5895 |
| Treatment* Smoker | -0.1812 | -0.6006 | 0.2382 | 0.2133 | 365 | -0.8496 | 0.3961 |
| **Post-rapid response: main effects model** | |  |  |  |  |  |  |
| Intercept | -0.3343 | -0.6348 | -0.0337 | 0.1526 | 261 | -2.1900 | 0.0294 |
| Treatment | 0.6097 | 0.3828 | 0.8367 | 0.1153 | 261 | 5.2898 | 0.0000 |
| Smoker | 0.1319 | -0.1406 | 0.4045 | 0.1384 | 261 | 0.9533 | 0.3413 |
| **Post-rapid response: model with moderation (interaction) effect** | | | |  |  |  |  |
| Intercept | -0.3567 | -0.6715 | -0.0420 | 0.1599 | 260 | -2.2316 | 0.0265 |
| Treatment | 0.6520 | 0.3719 | 0.9322 | 0.1423 | 260 | 4.5829 | 0.0000 |
| Smoker | 0.1910 | -0.1671 | 0.5491 | 0.1819 | 260 | 1.0502 | 0.2946 |
| Treatment* Smoker | -0.1244 | -0.6057 | 0.3569 | 0.2444 | 260 | -0.5089 | 0.6113 |
| **Rapid response: main effects model** |  |  |  |  |  |  |  |
| Intercept | -0.3844 | -0.5836 | -0.1852 | 0.1013 | 420 | -3.7934 | 0.0002 |
| Treatment | 0.7550 | 0.5793 | 0.9307 | 0.0894 | 420 | 8.4487 | 0.0000 |
| Dosingweight(natLog) | 0.1026 | 0.0125 | 0.1928 | 0.0459 | 420 | 2.2388 | 0.0257 |
| **Rapid response: model with moderation (interaction) effect** | | |  |  |  |  |  |
| Intercept | -0.3821 | -0.5805 | -0.1836 | 0.1009 | 419 | -3.7847 | 0.0002 |
| Treatment | 0.7539 | 0.5785 | 0.9292 | 0.0892 | 419 | 8.4496 | 0.0000 |
| Dosingweight(natLog) | 0.0208 | -0.1163 | 0.1578 | 0.0697 | 419 | 0.2981 | 0.7657 |
| Treatment* Dosingweight(natLog) | 0.1376 | -0.0362 | 0.3114 | 0.0884 | 419 | 1.5559 | 0.1205 |
| **Post-rapid response: main effects model** | |  |  |  |  |  |  |
| Intercept | -0.3295 | -0.5932 | -0.0658 | 0.1342 | 396 | -2.4562 | 0.0145 |
| Treatment | 0.4776 | 0.3034 | 0.6519 | 0.0886 | 396 | 5.3885 | 0.0000 |
| Dosingweight(natLog) | 0.0684 | -0.0212 | 0.1580 | 0.0456 | 396 | 1.5013 | 0.1341 |
| **Post-rapid response: model with moderation (interaction) effect** | | | |  |  |  |  |
| Intercept | -0.3287 | -0.5951 | -0.0623 | 0.1355 | 395 | -2.4259 | 0.0157 |
| Treatment | 0.4789 | 0.3049 | 0.6529 | 0.0885 | 395 | 5.4104 | 0.0000 |
| Dosingweight(natLog) | -0.0051 | -0.1399 | 0.1296 | 0.0685 | 395 | -0.0747 | 0.9405 |
| Treatment* Dosingweight(natLog) | 0.1248 | -0.0458 | 0.2954 | 0.0868 | 395 | 1.4383 | 0.1511 |
| **Rapid response: main effects model** |  |  |  |  |  |  |  |
| Intercept | -0.4047 | -0.5924 | -0.2169 | 0.0955 | 384 | -4.2375 | 0.0000 |
| Treatment | 0.7241 | 0.5557 | 0.8925 | 0.0857 | 384 | 8.4528 | 0.0000 |
| BMI(natLog) | 0.1803 | 0.0935 | 0.2671 | 0.0441 | 384 | 4.0851 | 0.0001 |
| **Rapid response: model with moderation (interaction) effect** | | |  |  |  |  |  |
| Intercept | -0.4062 | -0.5938 | -0.2186 | 0.0954 | 383 | -4.2575 | 0.0000 |
| Treatment | 0.7256 | 0.5575 | 0.8936 | 0.0855 | 383 | 8.4892 | 0.0000 |
| BMI(natLog) | 0.1005 | -0.0273 | 0.2283 | 0.0650 | 383 | 1.5461 | 0.1229 |
| Treatment* BMI(natLog) | 0.1435 | -0.0253 | 0.3122 | 0.0858 | 383 | 1.6715 | 0.0954 |
| **Post-rapid response: main effects model** | |  |  |  |  |  |  |
| Intercept | -0.4153 | -0.6196 | -0.2109 | 0.1039 | 364 | -3.9952 | 0.0001 |
| Treatment | 0.5098 | 0.3332 | 0.6864 | 0.0898 | 364 | 5.6758 | 0.0000 |
| BMI(natLog) | 0.1289 | 0.0392 | 0.2185 | 0.0456 | 364 | 2.8277 | 0.0049 |
| **Post-rapid response: model with moderation (interaction) effect** | | | |  |  |  |  |
| Intercept | -0.4176 | -0.6233 | -0.2118 | 0.1046 | 363 | -3.9910 | 0.0001 |
| Treatment | 0.5126 | 0.3361 | 0.6891 | 0.0897 | 363 | 5.7124 | 0.0000 |
| BMI(natLog) | 0.0637 | -0.0677 | 0.1951 | 0.0668 | 363 | 0.9540 | 0.3407 |
| Treatment* BMI(natLog) | 0.1180 | -0.0559 | 0.2918 | 0.0884 | 363 | 1.3339 | 0.1831 |
| **Rapid response: main effects model** |  |  |  |  |  |  |  |
| Intercept | -0.2752 | -0.4795 | -0.0709 | 0.1039 | 373 | -2.6487 | 0.0084 |
| Treatment | 0.6095 | 0.4175 | 0.8015 | 0.0976 | 373 | 6.2422 | 0.0000 |
| SystolicBloodPressure | 0.0794 | -0.0146 | 0.1735 | 0.0478 | 373 | 1.6605 | 0.0977 |
| **Rapid response: model with moderation (interaction) effect** | | |  |  |  |  |  |
| Intercept | -0.2900 | -0.4984 | -0.0815 | 0.1060 | 372 | -2.7350 | 0.0065 |
| Treatment | 0.6172 | 0.4252 | 0.8092 | 0.0976 | 372 | 6.3214 | 0.0000 |
| SystolicBloodPressure | -0.0061 | -0.1511 | 0.1390 | 0.0738 | 372 | -0.0824 | 0.9344 |
| Treatment* SystolicBloodPressure | 0.1462 | -0.0430 | 0.3354 | 0.0962 | 372 | 1.5191 | 0.1296 |
| **Post-rapid response: main effects model** | |  |  |  |  |  |  |
| Intercept | -0.0896 | -0.3682 | 0.1891 | 0.1417 | 355 | -0.6321 | 0.5277 |
| Treatment | 0.3648 | 0.1712 | 0.5585 | 0.0985 | 355 | 3.7046 | 0.0002 |
| SystolicBloodPressure | 0.1100 | 0.0157 | 0.2043 | 0.0479 | 355 | 2.2945 | 0.0223 |
| **Post-rapid response: model with moderation (interaction) effect** | | | |  |  |  |  |
| Intercept | -0.1150 | -0.3923 | 0.1623 | 0.1410 | 354 | -0.8156 | 0.4153 |
| Treatment | 0.3832 | 0.1901 | 0.5763 | 0.0982 | 354 | 3.9033 | 0.0001 |
| SystolicBloodPressure | -0.0244 | -0.1702 | 0.1214 | 0.0741 | 354 | -0.3293 | 0.7422 |
| Treatment* SystolicBloodPressure | 0.2286 | 0.0387 | 0.4185 | 0.0966 | 354 | 2.3673 | 0.0185 |
| **Rapid response: main effects model** |  |  |  |  |  |  |  |
| Intercept | -0.2776 | -0.4839 | -0.0714 | 0.1049 | 373 | -2.6475 | 0.0085 |
| Treatment | 0.6157 | 0.4229 | 0.8086 | 0.0981 | 373 | 6.2783 | 0.0000 |
| DiastolicBloodPressure | 0.0397 | -0.0544 | 0.1337 | 0.0478 | 373 | 0.8290 | 0.4076 |
| **Rapid response: model with moderation (interaction) effect** | | |  |  |  |  |  |
| Intercept | -0.2887 | -0.4988 | -0.0787 | 0.1068 | 372 | -2.7026 | 0.0072 |
| Treatment | 0.6203 | 0.4273 | 0.8134 | 0.0982 | 372 | 6.3180 | 0.0000 |
| DiastolicBloodPressure | -0.0206 | -0.1678 | 0.1266 | 0.0749 | 372 | -0.2757 | 0.7829 |
| Treatment* DiastolicBloodPressure | 0.1015 | -0.0889 | 0.2918 | 0.0968 | 372 | 1.0481 | 0.2953 |
| **Post-rapid response: main effects model** | |  |  |  |  |  |  |
| Intercept | -0.0934 | -0.3777 | 0.1910 | 0.1446 | 355 | -0.6457 | 0.5189 |
| Treatment | 0.3713 | 0.1760 | 0.5666 | 0.0993 | 355 | 3.7396 | 0.0002 |
| DiastolicBloodPressure | 0.0493 | -0.0451 | 0.1438 | 0.0480 | 355 | 1.0275 | 0.3049 |
| **Post-rapid response: model with moderation (interaction) effect** | | | |  |  |  |  |
| Intercept | -0.1009 | -0.3867 | 0.1848 | 0.1453 | 354 | -0.6948 | 0.4877 |
| Treatment | 0.3755 | 0.1797 | 0.5713 | 0.0996 | 354 | 3.7712 | 0.0002 |
| DiastolicBloodPressure | 0.0091 | -0.1408 | 0.1589 | 0.0762 | 354 | 0.1189 | 0.9054 |
| Treatment* DiastolicBloodPressure | 0.0667 | -0.1260 | 0.2594 | 0.0980 | 354 | 0.6812 | 0.4962 |
| **Rapid response: main effects model** |  |  |  |  |  |  |  |
| Intercept | -0.3031 | -0.5149 | -0.0912 | 0.1077 | 361 | -2.8136 | 0.0052 |
| Treatment | 0.6285 | 0.4338 | 0.8231 | 0.0990 | 361 | 6.3499 | 0.0000 |
| BaselinePulse(natLog) | 0.0055 | -0.0894 | 0.1004 | 0.0483 | 361 | 0.1133 | 0.9098 |
| **Rapid response: model with moderation (interaction) effect** | | |  |  |  |  |  |
| Intercept | -0.3026 | -0.5136 | -0.0917 | 0.1073 | 360 | -2.8217 | 0.0050 |
| Treatment | 0.6276 | 0.4331 | 0.8221 | 0.0989 | 360 | 6.3470 | 0.0000 |
| BaselinePulse(natLog) | 0.0660 | -0.0663 | 0.1983 | 0.0673 | 360 | 0.9814 | 0.3271 |
| Treatment* BaselinePulse(natLog) | -0.1230 | -0.3102 | 0.0643 | 0.0952 | 360 | -1.2916 | 0.1973 |
| **Post-rapid response: main effects model** | |  |  |  |  |  |  |
| Intercept | -0.1036 | -0.3991 | 0.1918 | 0.1502 | 344 | -0.6900 | 0.4907 |
| Treatment | 0.3728 | 0.1771 | 0.5685 | 0.0995 | 344 | 3.7473 | 0.0002 |
| BaselinePulse(natLog) | 0.0349 | -0.0603 | 0.1301 | 0.0484 | 344 | 0.7211 | 0.4714 |
| **Post-rapid response: model with moderation (interaction) effect** | | | |  |  |  |  |
| Intercept | -0.1039 | -0.3967 | 0.1888 | 0.1489 | 343 | -0.6983 | 0.4855 |
| Treatment | 0.3686 | 0.1732 | 0.5639 | 0.0993 | 343 | 3.7113 | 0.0002 |
| BaselinePulse(natLog) | 0.1116 | -0.0217 | 0.2449 | 0.0678 | 343 | 1.6467 | 0.1005 |
| Treatment* BaselinePulse(natLog) | -0.1530 | -0.3396 | 0.0337 | 0.0949 | 343 | -1.6123 | 0.1078 |

| **Ketamine dose (mg): prediction in ketamine-treated sample only** | | | | | | | |
| --- | --- | --- | --- | --- | --- | --- | --- |
|  | **B* estimate** | **lower (95% CI)** | **upper (95% CI)** | **Std.Error** | **DF** | **t-value** | **p-value** |
| **Rapid response** |  |  |  |  |  |  |  |
| Intercept | 0.1017 | -0.2722 | 0.4756 | 0.1898 | 243 | 0.5358 | 0.5926 |
| KetamineDose | 0.0069 | -0.0014 | 0.0151 | 0.0042 | 243 | 1.6433 | 0.1016 |
| **Post-rapid response** |  |  |  |  |  |  |  |
| Intercept | -0.0347 | -0.4841 | 0.4146 | 0.2280 | 228 | -0.1523 | 0.8791 |
| KetamineDose | 0.0044 | -0.0045 | 0.0133 | 0.0045 | 228 | 0.9654 | 0.3354 |

## Publication bias assessment

Funnel plots were generated for the two main outcomes discussed in the main text (rapid and post-rapid % change from baseline), by plotting standardized beta weights for the difference between ketamine vs. placebo against standard error. The trim-and-fill method([1](#_ENREF_1)) indicated there were no studies missing from either analysis, suggesting no evidence of publication bias within the 17 studies compiled for present analyses.

# Supplemental References

1. Duval S, Tweedie R: Trim and fill: A simple funnel-plot-based method of testing and adjusting for publication bias in meta-analysis. Biometrics 2000;56(2):455-63
