# Supplementary material for: International pooled patient-level meta-analysis of ketamine infusion for depression: In search of clinical moderators
Source: Mol Psychiatry. 2022 Sep 7;27(12):5096–112. doi: 10.1038/s41380-022-01757-7 (PMC9763119; doi:10.1038/s41380-022-01757-7)

International Pooled Patient-Level Metaanalysis of Ketamine Infusion for Depression: In Search of Clinical Moderators

Supplemental Information #2

Information provided in each table below

The following tables provide the moderator effect sizes, confidence intervals, hypothesis test results, and moderator weights. These tables are presented separately for each M* model (Tier 1, Tiers 2a-2g), first for the rapid (~24-hour effect) outcome and then for the post-rapid (~7-day) outcome. In table headings, "Moderator effect size" denotes the effect size (r-value) of each variable operating as the sole moderator. To construct 95% confidence intervals, we used bootstrap confidence intervals with 200 resamples; the bootstrap was used as it's robust to nested data, allowing for valid inference when comparing across different studies. The significance column denotes whether the effect size is significantly different from 0. The weight column displays the weight each variable (moderator) is given in generating the combined moderator. Lastly, all tables include a variable called "Overall" which corresponds to the combined moderator (M*) for that specific Tier/outcome.

Information provided in each figure below

Figures below display the estimated outcome as a function of combined moderator (M*) scores and treatment. Additionally, the points correspond to each patients' combined moderator score and their observed outcome measurement. Figures can be used to help identify the differing behavior of the outcome as a function of the combined moderator score for each treatment group. They display how the combined moderator interacts with treatment group when estimating the outcome.

Rapid effects

Tier 1

Variable	Moderator effect size (r)	Confidence interval	Statistically significant (per 95% CI)?	Weight
Study–level TRD Threshold >= 2	0.083	(0.020, 0.162)	Yes	0.118
Inpatient vs. Outpatient	0.040	(–0.040, 0.106)	No	–0.037
Major Depressive Disorder (MDD)	–0.012	(–0.080, 0.051)	No	–0.145
Study Performed in the US (y/n)	0.041	(–0.032, 0.115)	No	–0.092
Age	0.028	(–0.049, 0.094)	No	–0.006
Female or Male	0.035	(–0.029, 0.116)	No	0.197
Overall	0.125	(0.045, 0.196)	Yes	

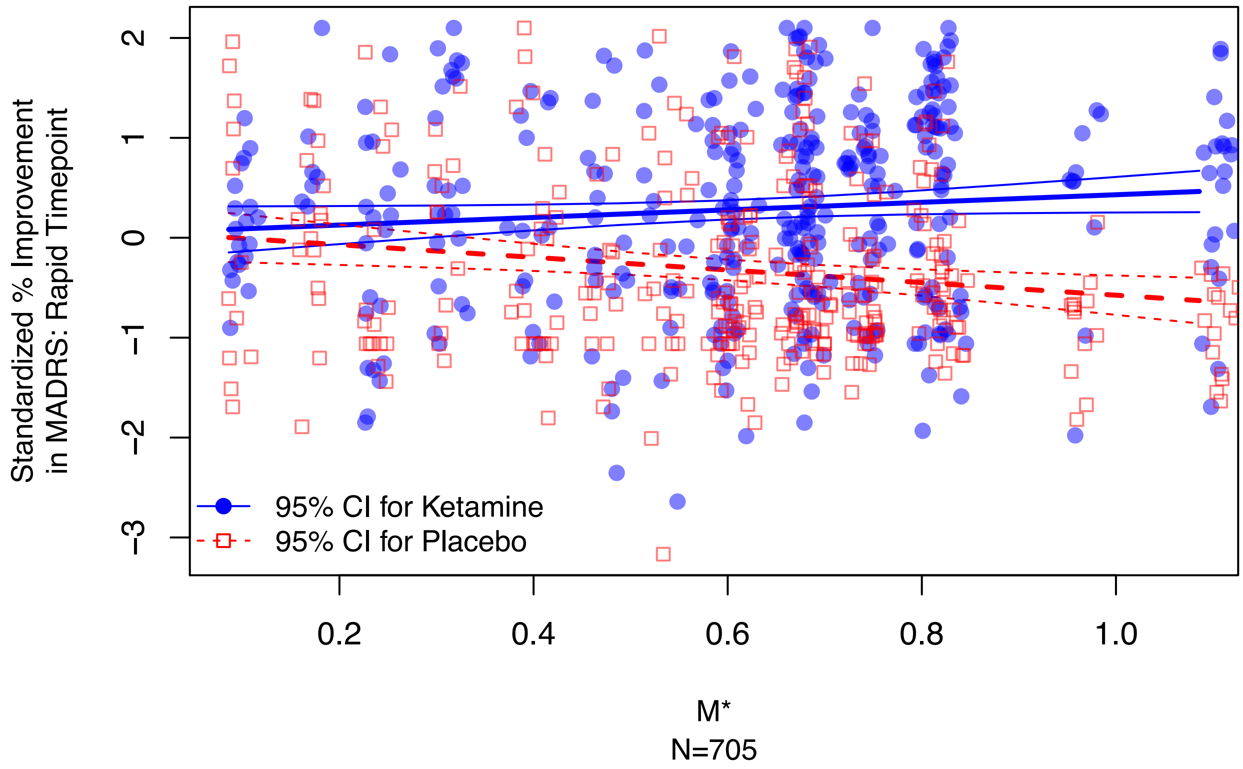

Tier2a

Variable	Moderator effect size (r)	Confidence interval	Statistically significant (per 95% CI)?	Weight
Study–level TRD Threshold >= 2	0.071	(–0.008, 0.139)	No	0.3
Inpatient vs. Outpatient	0.059	(–0.018, 0.139)	No	0.021
Major Depressive Disorder (MDD)	–0.022	(–0.099, 0.047)	No	–0.039
Study Performed in the US (y/n)	0.074	(0.007, 0.139)	Yes	0.459
Age	0.037	(–0.035, 0.113)	No	–0.142
Female or Male	0.022	(–0.043, 0.096)	No	0.318
Race is White (y/n)	0.017	(–0.065, 0.099)	No	0.174
Race is Black (y/n)	0.004	(–0.090, 0.112)	No	0
Race is Asian (y/n)	–0.002	(–0.071, 0.058)	No	–0.186
Race is Other (y/n)	–0.031	(–0.105, 0.060)	No	–0.531
Overall	0.124	(0.060, 0.198)	Yes	

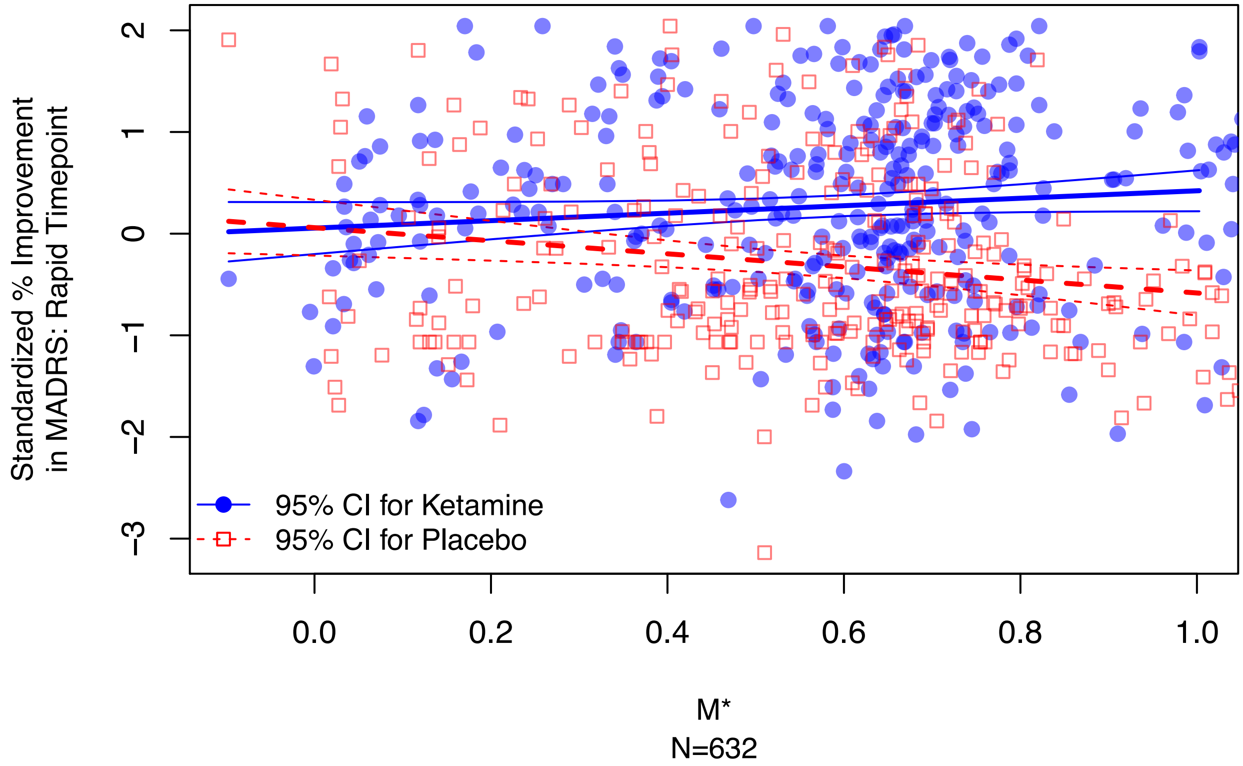

Tier2b

Variable	Moderator effect size (r)	Confidence interval	Statistically significant (per 95% CI)?	Weight
Study–level TRD Threshold >= 2	0.139	(0.057, 0.229)	Yes	0.864
Inpatient vs. Outpatient	0.062	(–0.033, 0.131)	No	–0.053
Major Depressive Disorder (MDD)	–0.020	(–0.102, 0.068)	No	–0.748
Study Performed in the US (y/n)	0.116	(0.024, 0.195)	Yes	0.095
Age	0.069	(–0.008, 0.146)	No	0.041
Female or Male	0.060	(–0.029, 0.135)	No	0.009
Any Concurrent Psych Med (y/n)	0.030	(–0.055, 0.115)	No	–0.352
Any Benzodiazepine (y/n)	–0.018	(–0.104, 0.061)	No	0.115
Overall	0.196	(0.120, 0.278)	Yes	

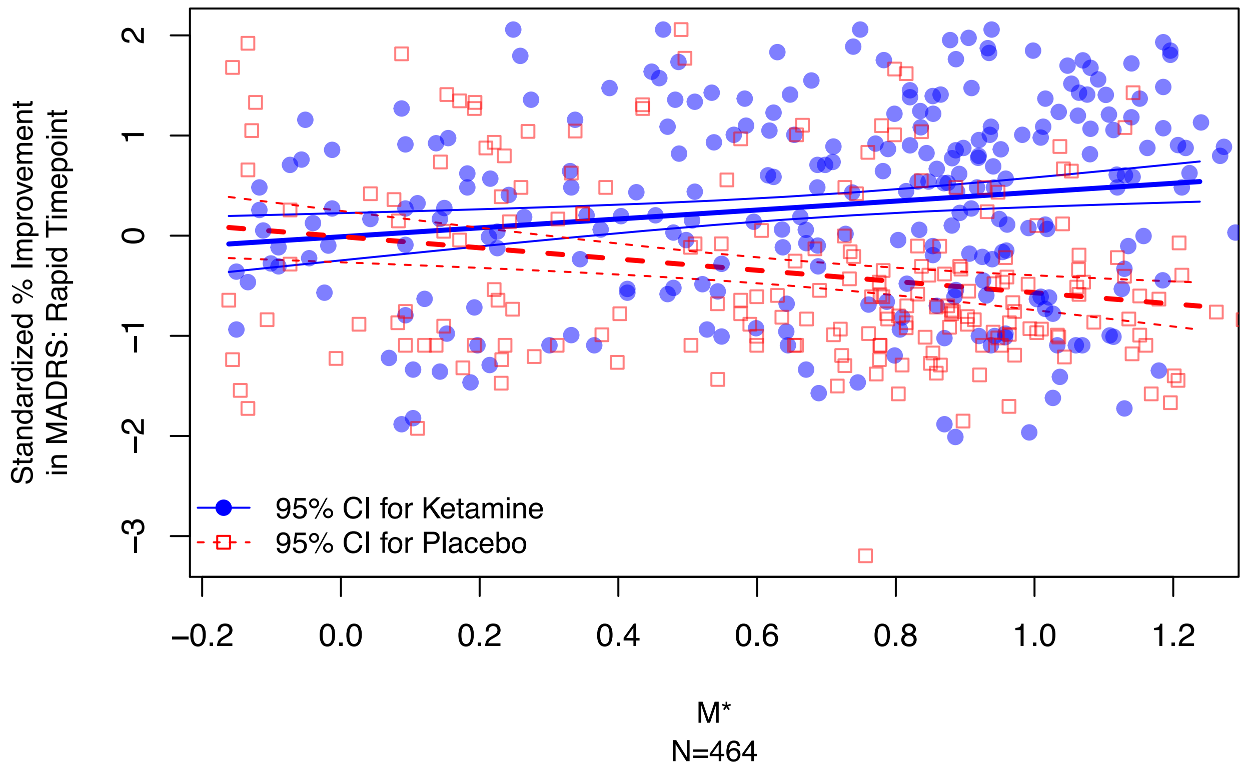

Tier2c

Variable	Moderator effect size (r)	Confidence interval	Statistically significant (per 95% CI)?	Weight
Study–level TRD Threshold >= 2	0.099	(–0.009, 0.201)	No	0.506
Inpatient vs. Outpatient	–0.030	(–0.140, 0.089)	No	0.184
Major Depressive Disorder (MDD)	–0.074	(–0.167, 0.034)	No	–0.673
Study Performed in the US (y/n)	0.049	(–0.045, 0.132)	No	0.563
Age	0.052	(–0.040, 0.153)	No	0.215
Female or Male	0.030	(–0.079, 0.133)	No	–0.033
Duration of Current MDE (months)	0.051	(–0.050, 0.155)	No	0.188
Recurrent MDD (y/n)	0.035	(–0.065, 0.134)	No	0.051
Number of Depressive Episodes	0.062	(–0.022, 0.161)	No	–0.053
Overall	0.202	(0.102, 0.280)	Yes	

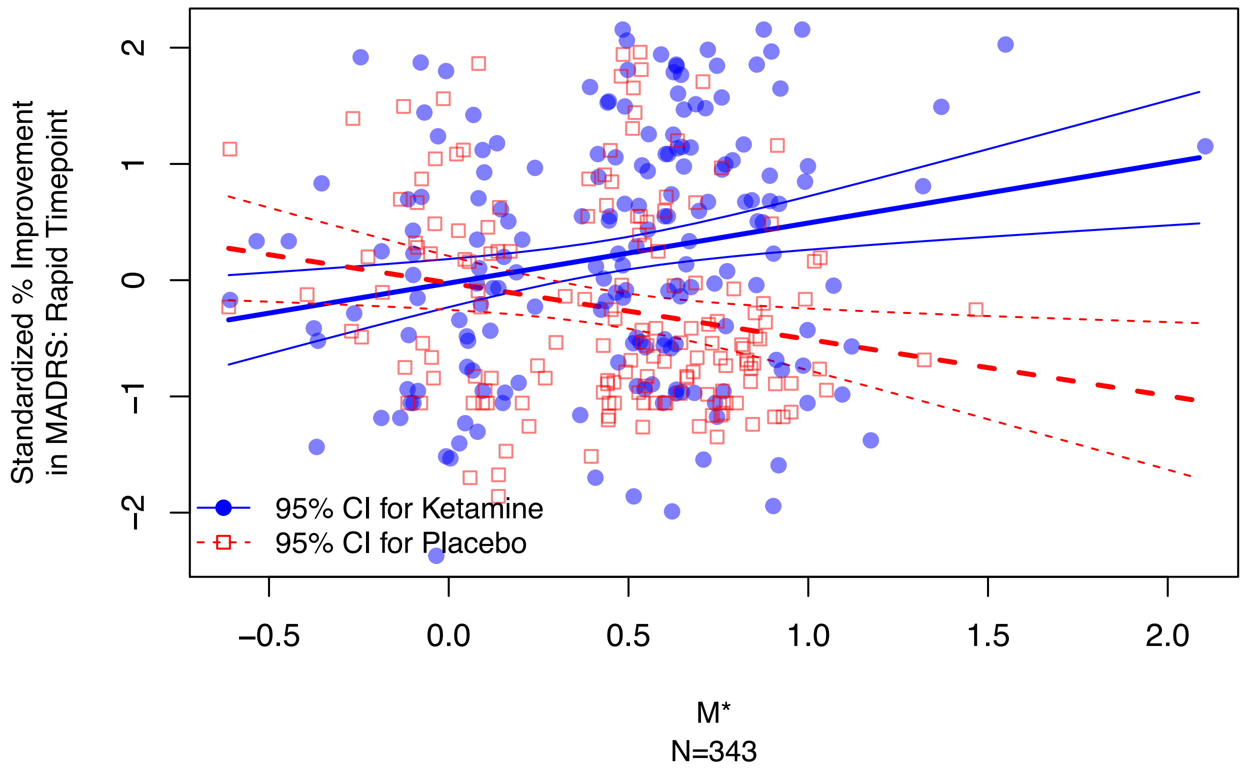

Tier2d

Variable	Moderator effect size (r)	Confidence interval	Statistically significant (per 95% CI)?	Weight
Study–level TRD Threshold >= 2	0.140	(0.047, 0.224)	Yes	1.06
Inpatient vs. Outpatient	–0.023	(–0.102, 0.071)	No	0.577
Major Depressive Disorder (MDD)	0.006	(–0.094, 0.082)	No	–0.461
Study Performed in the US (y/n)	0.045	(–0.035, 0.120)	No	0.063
Age	0.040	(–0.044, 0.116)	No	0.065
Female or Male	0.067	(–0.030, 0.163)	No	0.472
Generalized Anxiety Disorder	0.017	(–0.077, 0.094)	No	–0.259
Any Anxiety Disorder	–0.015	(–0.103, 0.063)	No	0.212
Overall	0.191	(0.098, 0.272)	Yes	

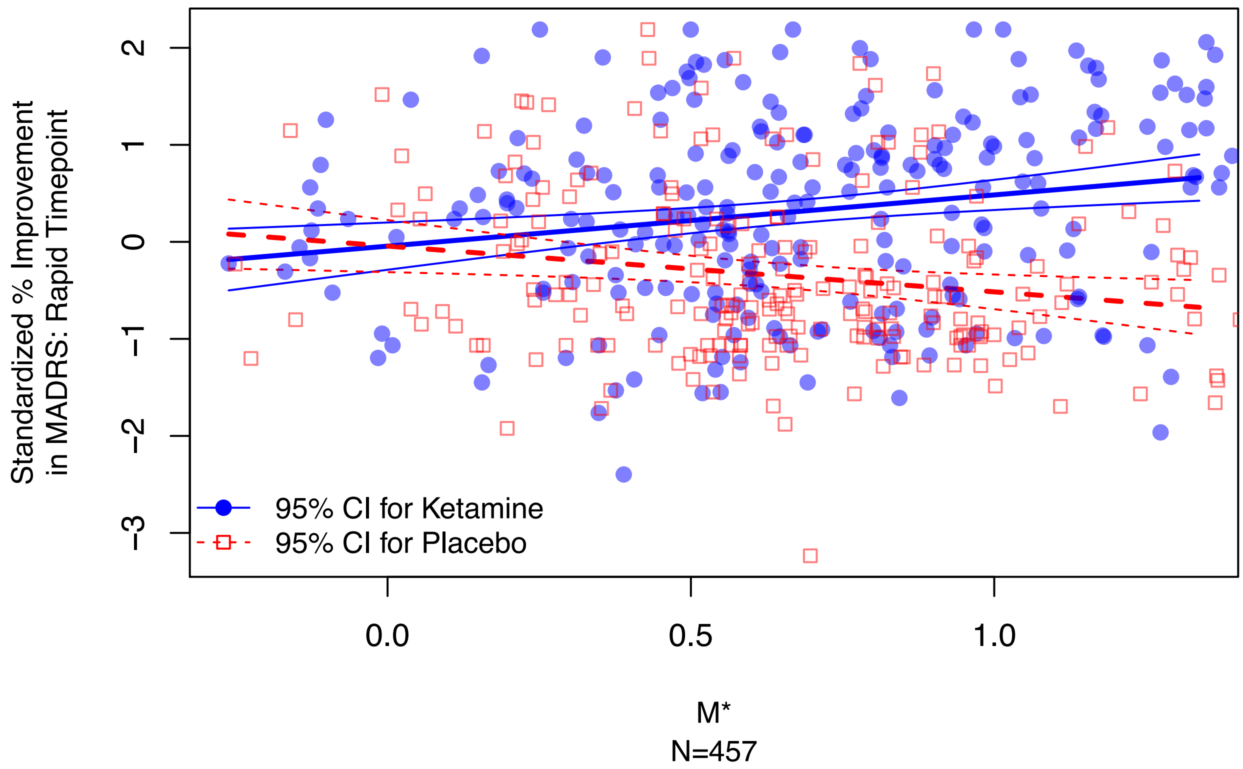

Tier2e

Variable	Moderator effect size (r)	Confidence interval	Statistically significant (per 95% CI)?	Weight
Study–level TRD Threshold >= 2	0.126	(0.021, 0.225)	Yes	0.418
Inpatient vs. Outpatient	–0.124	(–0.213, –0.010)	No	–0.859
Major Depressive Disorder (MDD)	0.016	(–0.088, 0.148)	No	–0.458
Study Performed in the US (y/n)	0.030	(–0.053, 0.126)	No	–0.228
Age	0.032	(–0.039, 0.117)	No	0.208
Female or Male	0.052	(–0.034, 0.162)	No	0.288
Systolic Blood Pressure	0.059	(–0.048, 0.159)	No	0.132
Diastolic Blood Pressure	0.042	(–0.061, 0.142)	No	–0.126
Overall	0.165	(0.078, 0.243)	Yes	

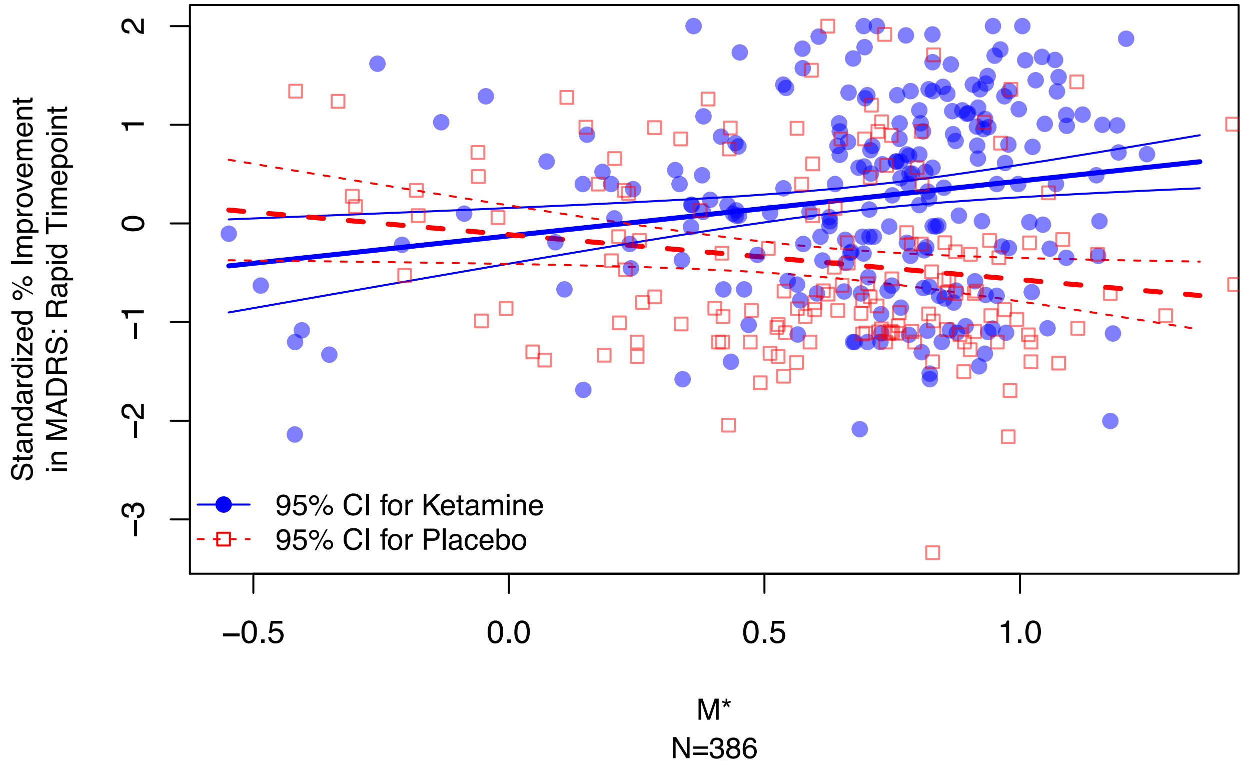

Tier2f

Variable	Moderator effect size (r)	Confidence interval	Statistically significant (per 95% CI)?	Weight
Study–level TRD Threshold >= 2	0.151	(0.037, 0.263)	Yes	1.091
Inpatient vs. Outpatient	0.016	(–0.107, 0.125)	No	0.108
Major Depressive Disorder (MDD)	–0.062	(–0.175, 0.039)	No	–0.677
Study Performed in the US (y/n)	0.167	(0.062, 0.286)	Yes	0.619
Age	0.049	(–0.058, 0.136)	No	–0.125
Female or Male	–0.024	(–0.155, 0.094)	No	–0.153
Smoker (Dummy–Coded)	–0.028	(–0.160, 0.077)	No	–0.167
BMI	0.111	(–0.035, 0.231)	No	0.313
Overall	0.293	(0.175, 0.415)	Yes	

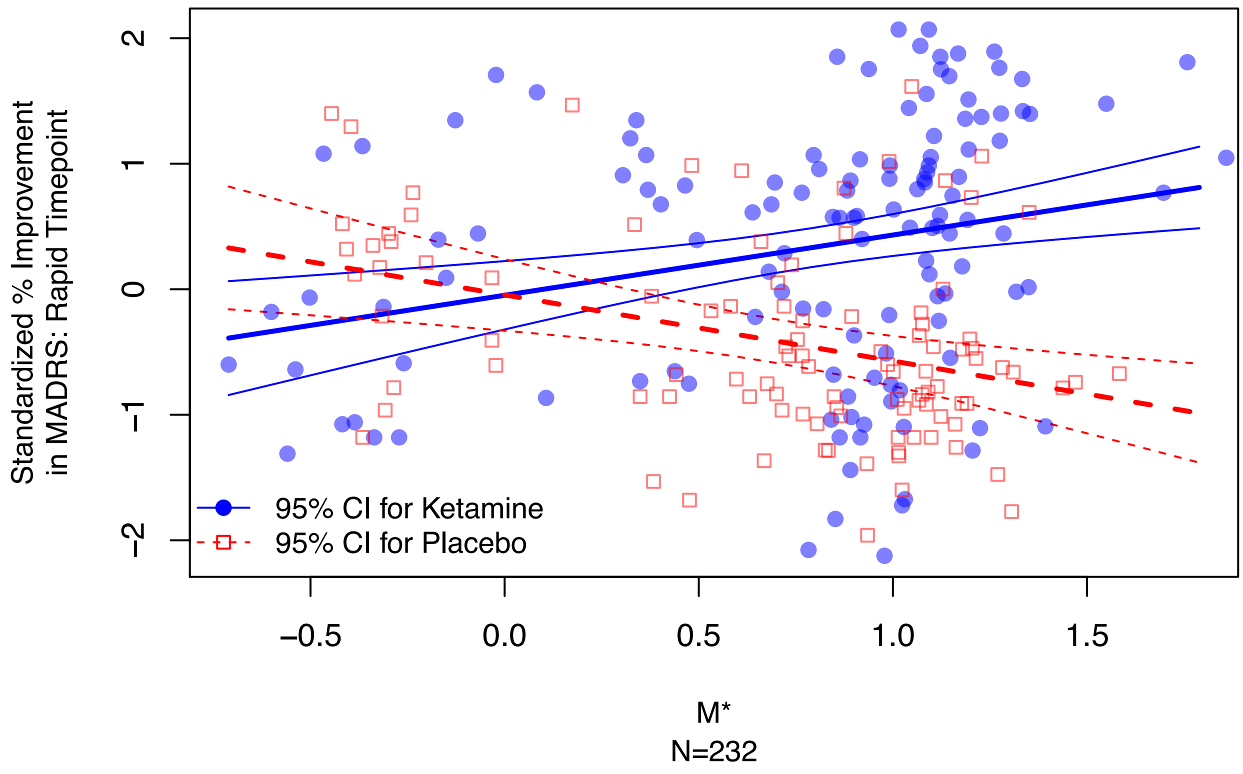

Tier2g

Variable	Moderator effect size (r)	Confidence interval	Statistically significant (per 95% CI)?	Weight
Study−level TRD Threshold >= 2	0.058	(−0.040, 0.147)	No	0.615
Inpatient vs. Outpatient	−0.047	(−0.150, 0.057)	No	0.239
Major Depressive Disorder (MDD)	0.019	(−0.091, 0.115)	No	−0.543
Study Performed in the US (y/n)	0.036	(−0.047, 0.120)	No	0.014
Age	0.034	(−0.044, 0.123)	No	−0.014
Female or Male	0.055	(−0.037, 0.137)	No	0.313
Years of Education	0.012	(−0.076, 0.104)	No	−0.011
Marital Status	−0.043	(−0.138, 0.042)	No	0.046
Overall	0.118	(0.024, 0.199)	Yes	

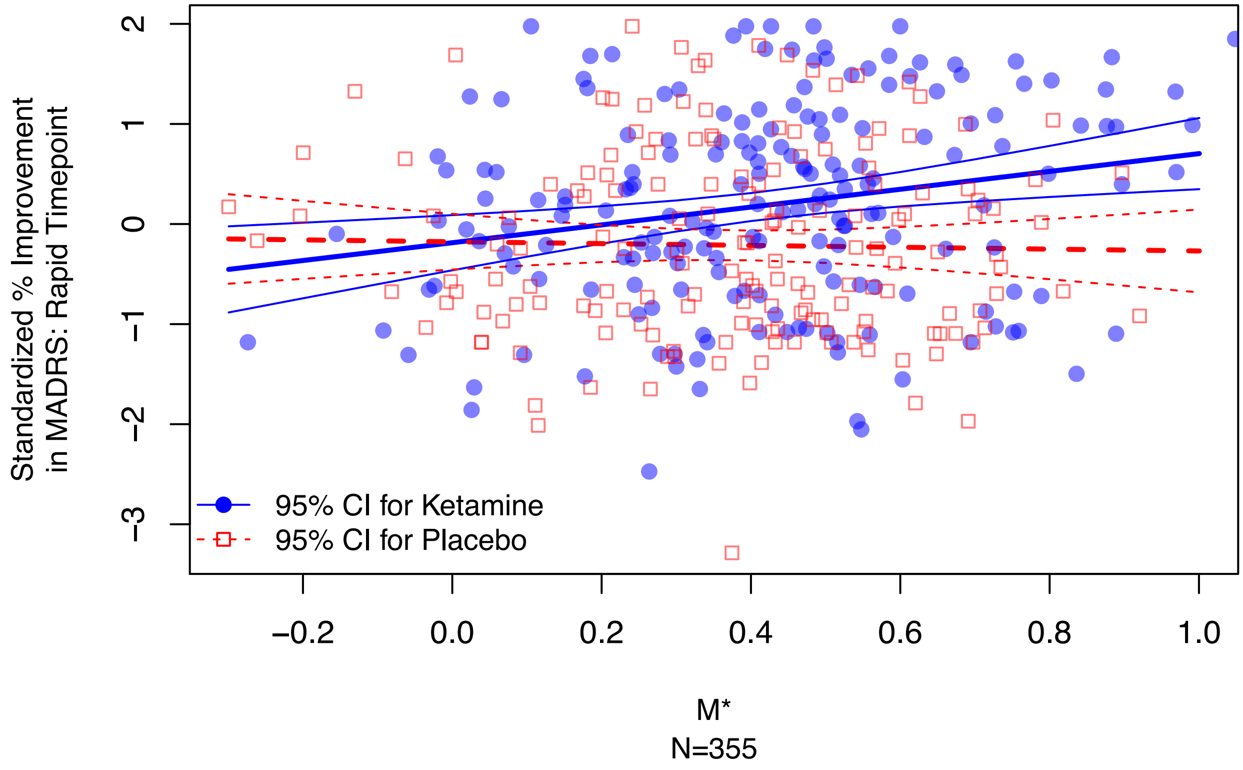

Post-rapid effects

Tier 1

Variable	Moderator effect size (r)	Confidence interval	Statistically significant (per 95% CI)?	Weight
Study–level TRD Threshold >= 2	0.108	(0.038, 0.174)	Yes	0.657
Inpatient vs. Outpatient	0.006	(–0.056, 0.073)	No	–0.112
Major Depressive Disorder (MDD)	–0.027	(–0.107, 0.042)	No	–0.463
Study Performed in the US (y/n)	0.089	(0.013, 0.160)	Yes	–0.132
Age	0.061	(–0.013, 0.129)	No	0.114
Female or Male	–0.031	(–0.096, 0.052)	No	–0.155
Overall	0.144	(0.074, 0.220)	Yes	

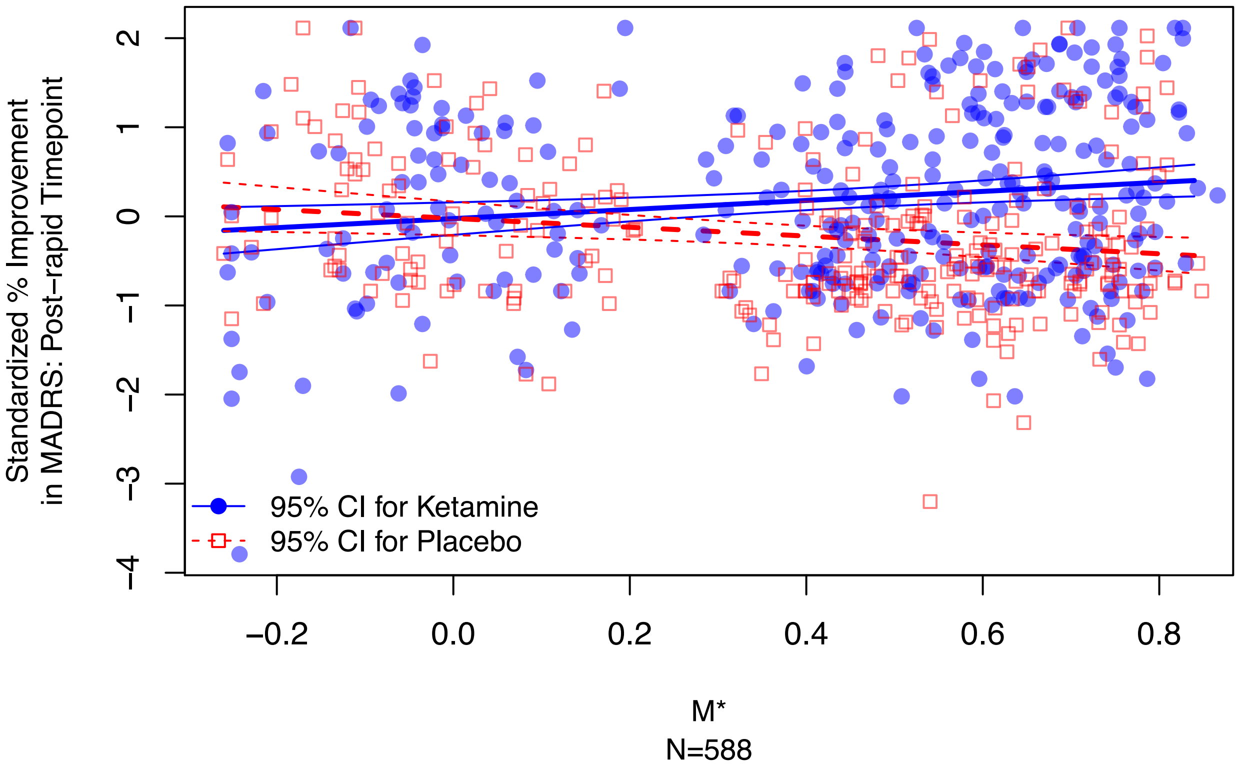

Tier2a

Variable	Moderator effect size (r)	Confidence interval	Statistically significant (per 95% CI)?	Weight
Study−level TRD Threshold >= 2	0.104	(0.027, 0.199)	Yes	0.608
Inpatient vs. Outpatient	0.021	(−0.052, 0.093)	No	0.577
Major Depressive Disorder (MDD)	−0.039	(−0.116, 0.051)	No	−0.083
Study Performed in the US (y/n)	0.116	(0.027, 0.195)	Yes	0.55
Age	0.079	(0.003, 0.154)	Yes	0.073
Female or Male	−0.063	(−0.143, 0.013)	No	−0.406
Race is White (y/n)	−0.044	(−0.127, 0.048)	No	−0.151
Race is Black (y/n)	0.074	(0.002, 0.159)	Yes	0.124
Race is Asian (y/n)	0.019	(−0.051, 0.095)	No	0.553
Race is Other (y/n)	−0.028	(−0.126, 0.043)	No	0
Overall	0.169	(0.076, 0.239)	Yes	

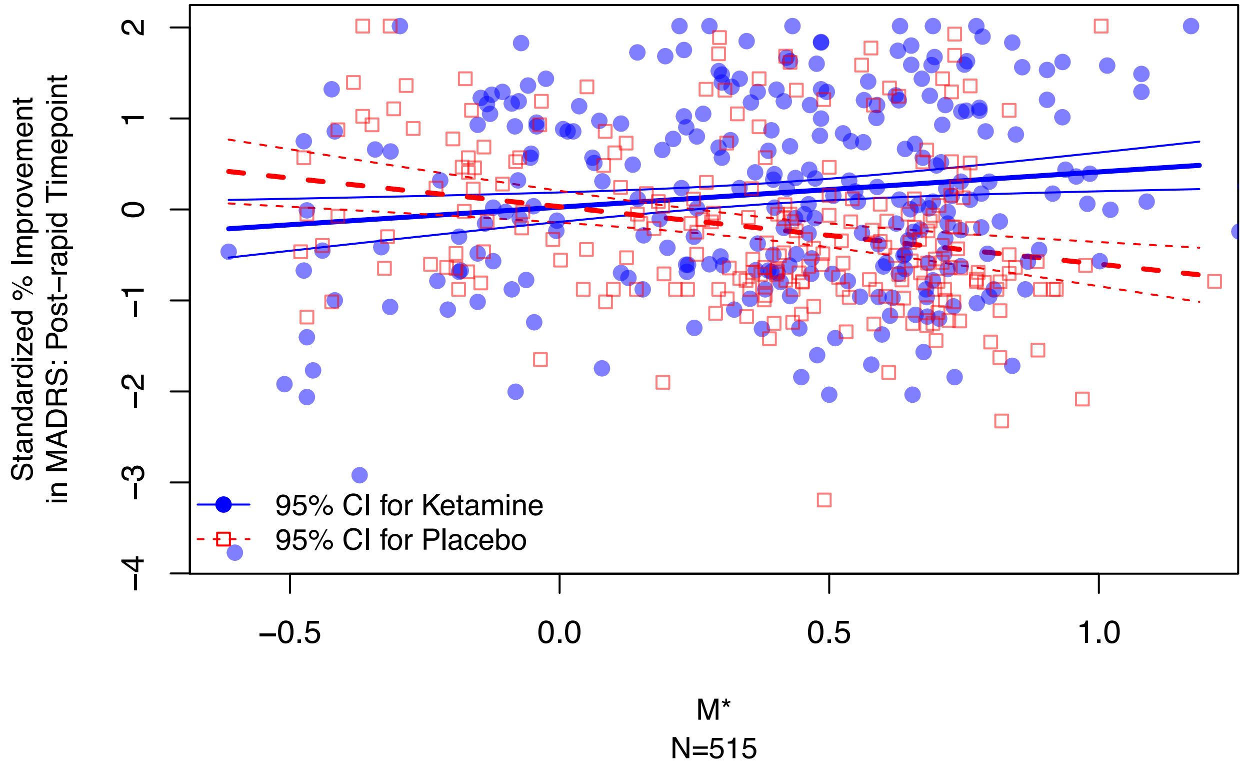

Tier2b

Variable	Moderator effect size (r)	Confidence interval	Statistically significant (per 95% CI)?	Weight
Study–level TRD Threshold >= 2	0.158	(0.074, 0.239)	Yes	0.925
Inpatient vs. Outpatient	0.020	(–0.050, 0.093)	No	0
Major Depressive Disorder (MDD)	–0.020	(–0.096, 0.063)	No	–0.536
Study Performed in the US (y/n)	0.119	(0.019, 0.205)	Yes	0.219
Age	0.066	(–0.018, 0.156)	No	0.038
Female or Male	–0.011	(–0.101, 0.077)	No	0.292
Any Concurrent Psych Med (y/n)	0.002	(–0.084, 0.087)	No	–0.264
Any Benzodiazepine (y/n)	0.015	(–0.048, 0.104)	No	–0.109
Overall	0.199	(0.121, 0.283)	Yes	

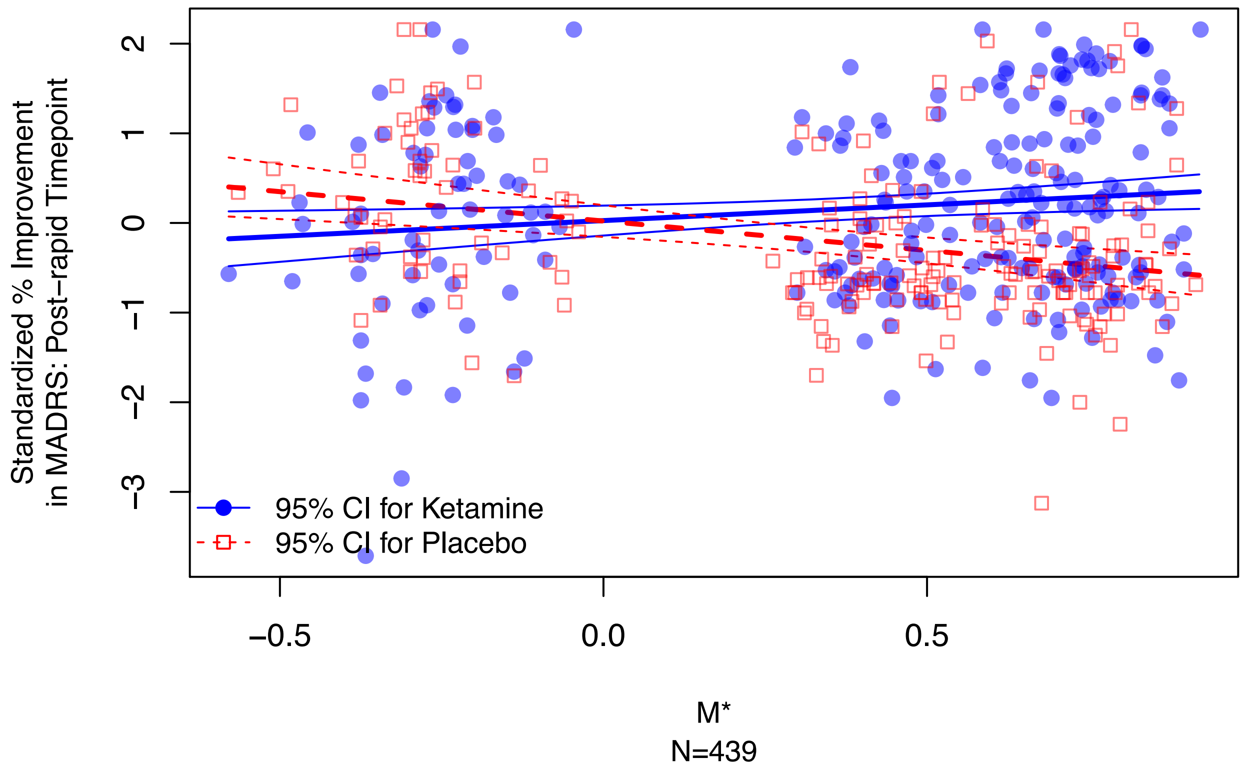

Tier2c

Variable	Moderator effect size (r)	Confidence interval	Statistically significant (per 95% CI)?	Weight
Study–level TRD Threshold >= 2	0.093	(–0.006, 0.207)	No	0.332
Inpatient vs. Outpatient	–0.053	(–0.178, 0.047)	No	–0.312
Major Depressive Disorder (MDD)	–0.019	(–0.111, 0.075)	No	–0.437
Study Performed in the US (y/n)	0.113	(0.004, 0.219)	Yes	0.449
Age	0.056	(–0.047, 0.176)	No	0.308
Female or Male	0.089	(–0.042, 0.208)	No	0.629
Duration of Current MDE (months)	–0.065	(–0.186, 0.030)	No	–0.273
Recurrent MDD (y/n)	0.055	(–0.053, 0.181)	No	–0.749
Number of Depressive Episodes	0.096	(–0.003, 0.205)	No	0.17
Overall	0.210	(0.107, 0.313)	Yes	

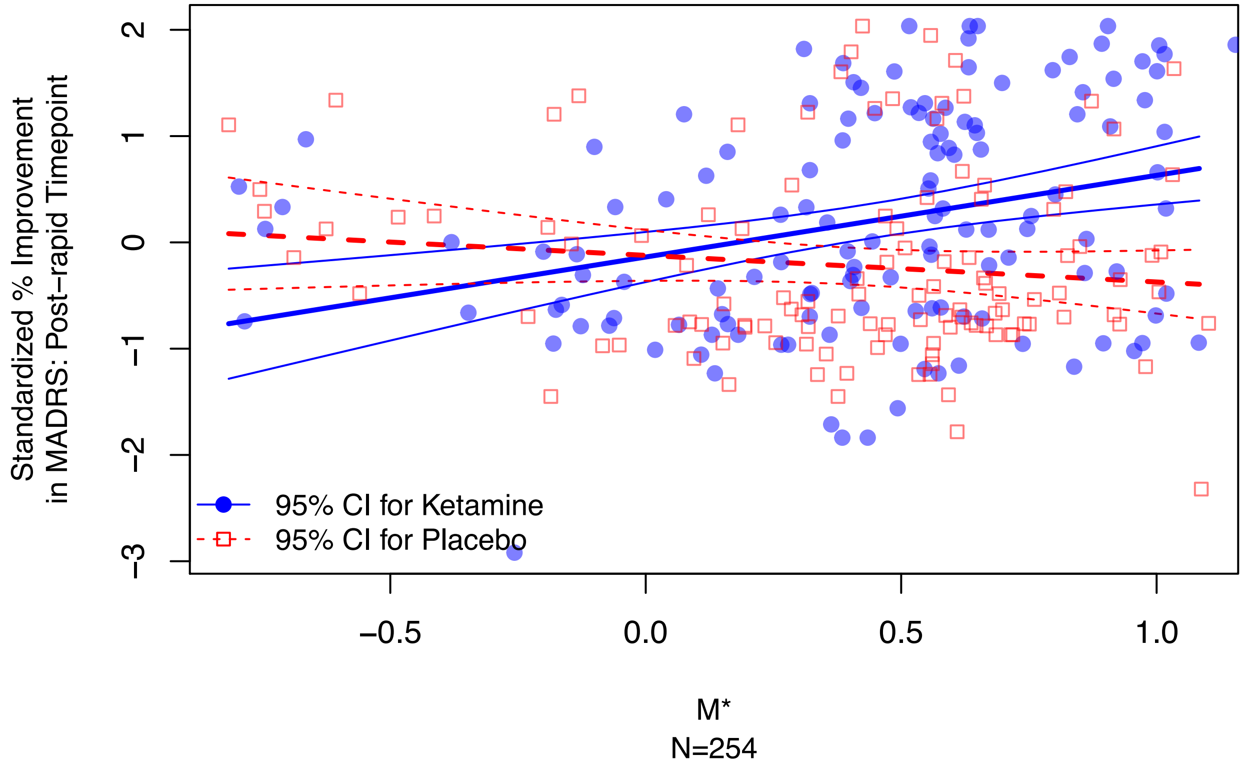

Tier2d

Variable	Moderator effect size (r)	Confidence interval	Statistically significant (per 95% CI)?	Weight
Study–level TRD Threshold >= 2	0.090	(0.007, 0.195)	Yes	0.372
Inpatient vs. Outpatient	–0.027	(–0.123, 0.052)	No	–0.525
Major Depressive Disorder (MDD)	0.001	(–0.085, 0.095)	No	–0.514
Study Performed in the US (y/n)	0.069	(–0.022, 0.164)	No	0.438
Age	–0.040	(–0.143, 0.060)	No	–0.128
Female or Male	0.037	(–0.053, 0.150)	No	0.34
Generalized Anxiety Disorder	–0.026	(–0.146, 0.089)	No	–0.009
Any Anxiety Disorder	–0.003	(–0.106, 0.111)	No	–0.161
Overall	0.156	(0.042, 0.247)	Yes	

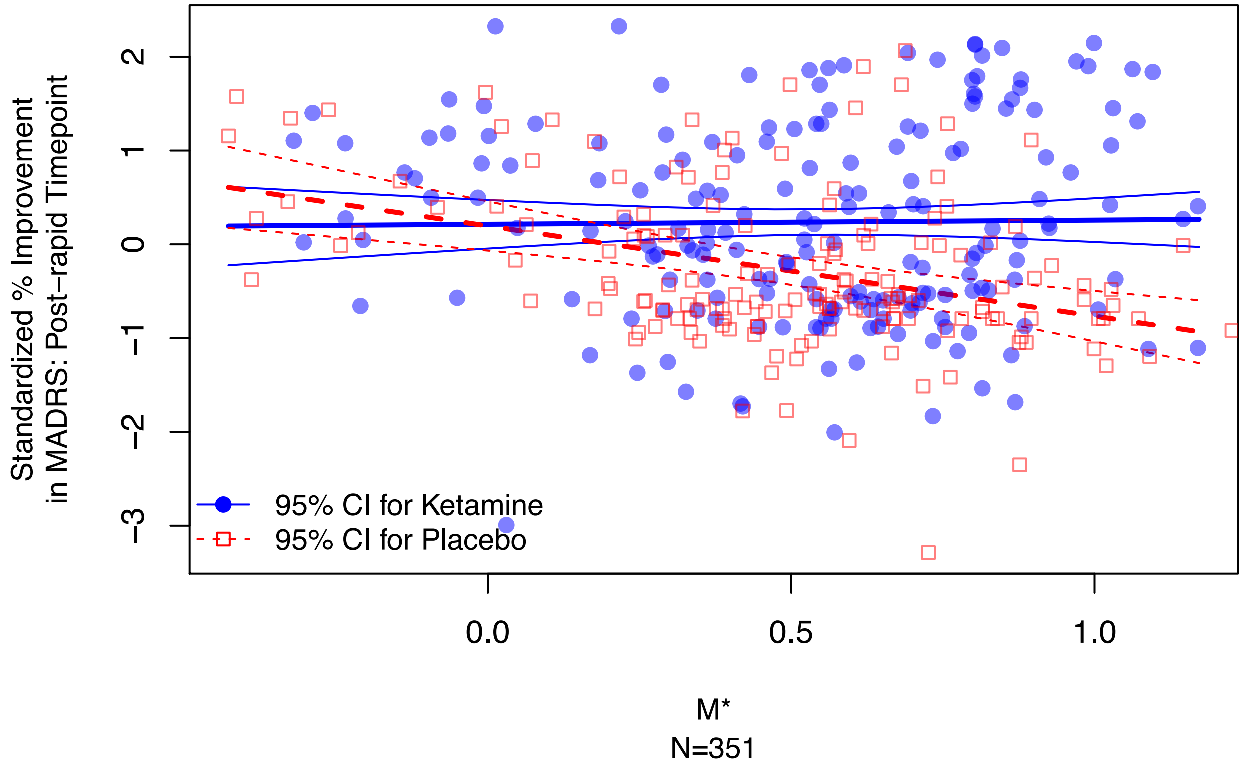

Tier2e

Variable	Moderator effect size (r)	Confidence interval	Statistically significant (per 95% CI)?	Weight
Study–level TRD Threshold >= 2	0.158	(0.040, 0.246)	Yes	0.937
Inpatient vs. Outpatient	–0.105	(–0.195, –0.019)	No	–0.709
Major Depressive Disorder (MDD)	–0.009	(–0.111, 0.085)	No	–1.223
Study Performed in the US (y/n)	0.070	(–0.029, 0.171)	No	–0.124
Age	0.059	(–0.047, 0.152)	No	0.108
Female or Male	–0.063	(–0.176, 0.036)	No	–0.111
Systolic Blood Pressure	0.106	(–0.006, 0.207)	No	0.168
Diastolic Blood Pressure	0.032	(–0.073, 0.112)	No	0.065
Overall	0.228	(0.139, 0.322)	Yes	

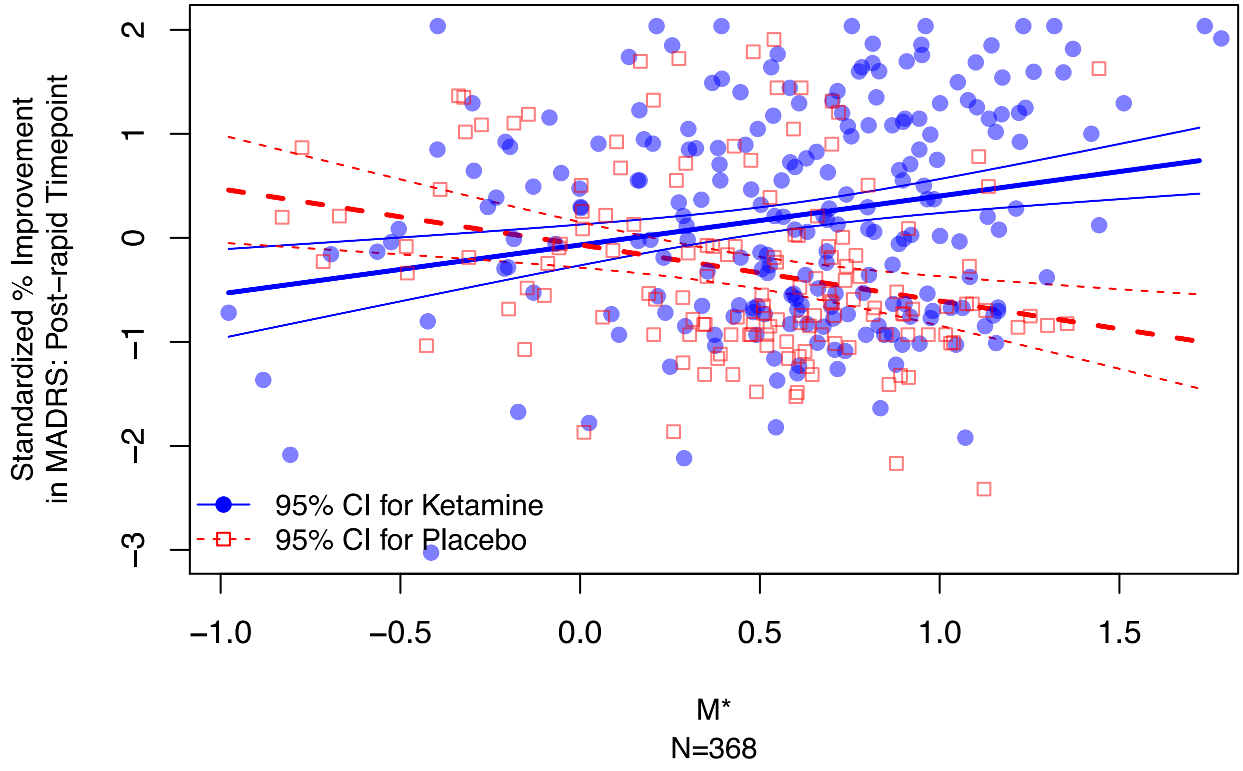

Tier2f

Variable	Moderator effect size (r)	Confidence interval	Statistically significant (per 95% CI)?	Weight
Study–level TRD Threshold >= 2	0.103	(0.005, 0.214)	Yes	0.256
Inpatient vs. Outpatient	−0.093	(−0.209, 0.019)	No	−0.286
Major Depressive Disorder (MDD)	0.020	(−0.086, 0.124)	No	0.1
Study Performed in the US (y/n)	0.068	(−0.034, 0.165)	No	0.448
Age	−0.098	(−0.212, 0.038)	No	−0.328
Female or Male	0.082	(−0.026, 0.224)	No	0.039
Smoker (Dummy–Coded)	−0.035	(−0.156, 0.077)	No	0.471
BMI	0.112	(0.026, 0.249)	Yes	0.106
Overall	0.234	(0.118, 0.347)	Yes	

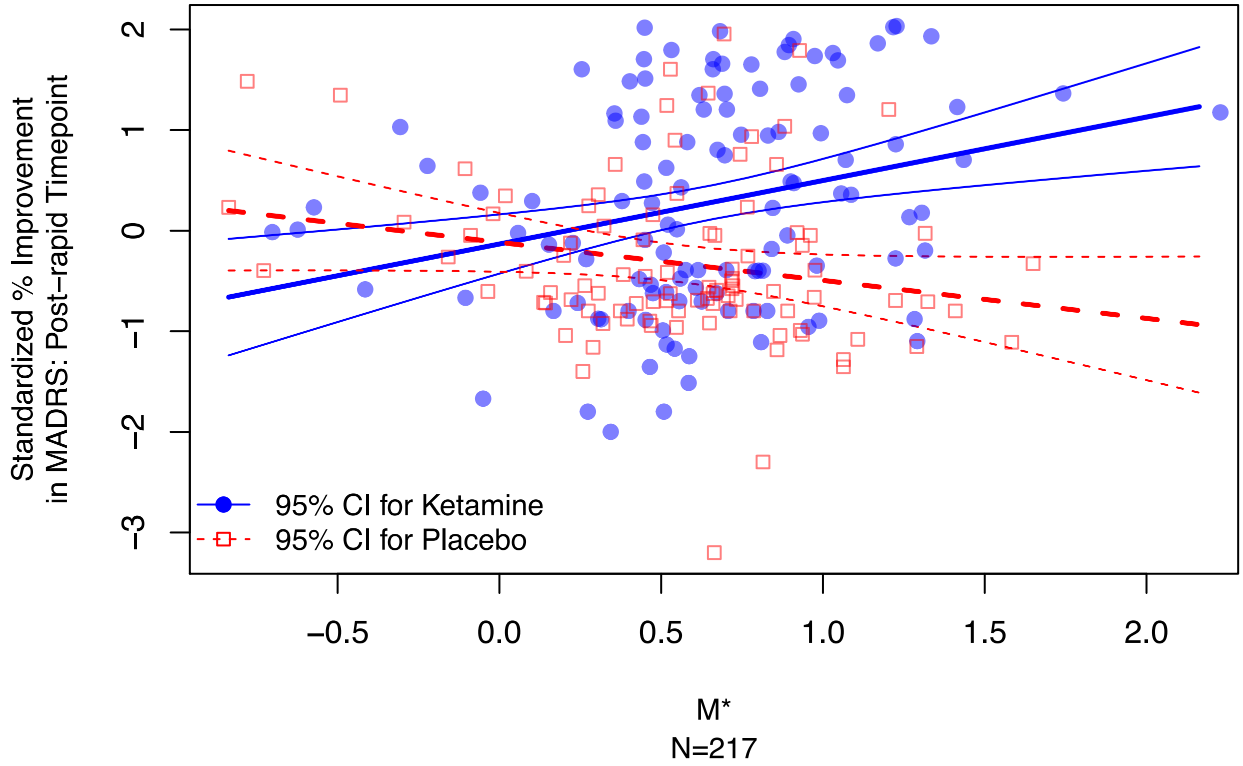

Tier2g

Variable	Moderator effect size (r)	Confidence interval	Statistically significant (per 95% CI)?	Weight
Study–level TRD Threshold >= 2	0.053	(–0.070, 0.189)	No	0.106
Inpatient vs. Outpatient	–0.101	(–0.182, –0.023)	No	–0.587
Major Depressive Disorder (MDD)	–0.041	(–0.149, 0.086)	No	–0.282
Study Performed in the US (y/n)	0.070	(–0.022, 0.181)	No	0.165
Age	0.021	(–0.106, 0.137)	No	–0.089
Female or Male	–0.045	(–0.166, 0.041)	No	–0.201
Years of Education	0.003	(–0.118, 0.118)	No	–0.013
Marital Status	–0.077	(–0.202, 0.048)	No	–0.058
Overall	0.151	(0.015, 0.275)	Yes	

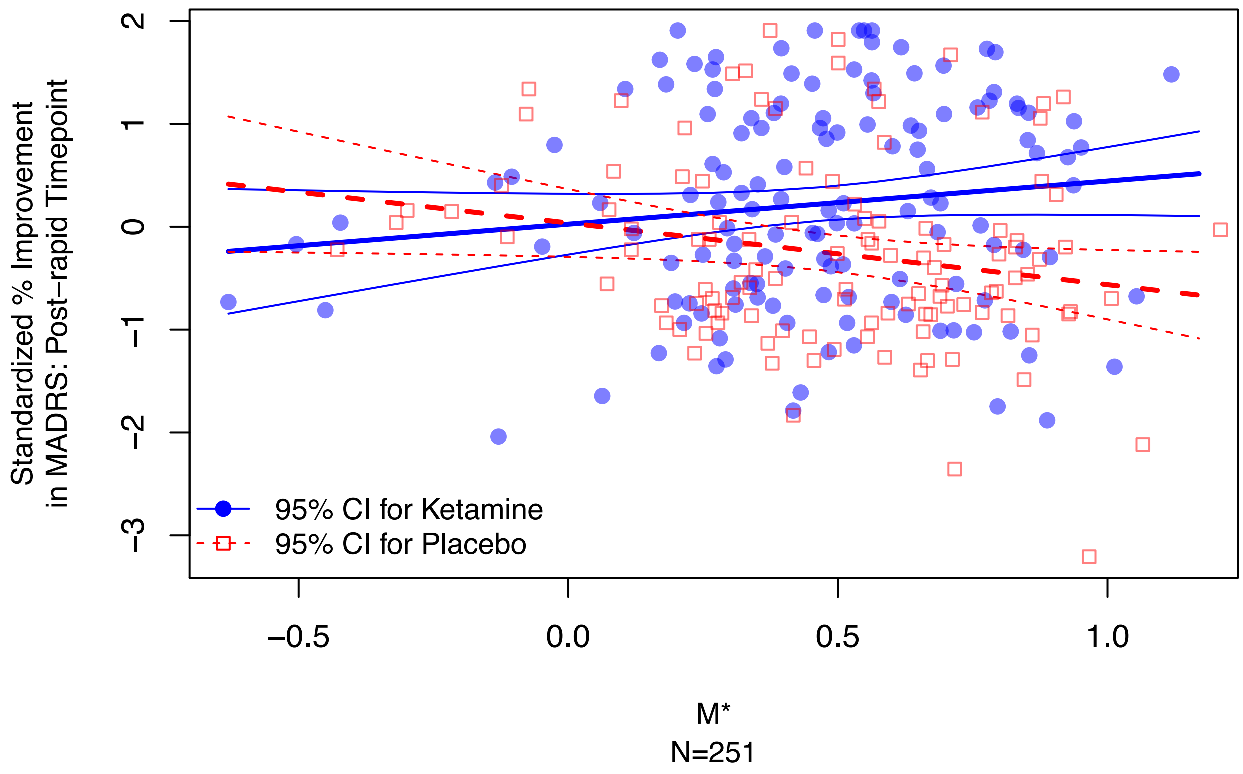

Supplement: Supplementary file 2 — Supplement 2 [file 41380_2022_1757_MOESM2_ESM.pdf]
